# Supplementary material for: Disease‐specific phenotypes in iPSC‐derived neural stem cells with POLG mutations
Source: EMBO Mol Med. 2020 Aug 25;12(10):e12146. doi: 10.15252/emmm.202012146 (PMC7539330; doi:10.15252/emmm.202012146)
Supplement: Supplementary file 1 — Appendix [file EMMM-12-e12146-s001.pdf]

List of Contents

Appendix Table S1	Summary of the iPSC or ESC clones used in the study
Appendix Figure S1	Representative histograms from flow cytometric analysis of the positive cell population stained with pluripotency markers POU5F1 and SSEA4 in ESCs and iPSCs derived from Detroit 551 control, WS5A and CP2A fibroblasts.
Appendix Figure S2	Flow cytometric analysis of the correlation between MTG and TMRE expression under different titrations (from 5 nM to 1 μM) of TMRE using TMRE and 150 nM MTG dual staining of control iPSCs.
Appendix Figure S3 .	Representative histograms of the positive staining with NSC marker NESTIN in NSCs derived from ESCs, Detroit 551 control, WS5A and CP2A iPSCs using flow cytometric analysis.
Appendix Figure S4	Representative images of immunostaining for astrocyte marker S100β from astrocytes derived from iPSC-NSCs.
Appendix Figure S5	Representative images of immunostaining for astrocyte markers EAAT1 and Glutamine Synthetase from astrocytes derived from iPSC-NSCs.
Appendix Figure S6	Representative images of immunostaining for OLIG2 and MBP from oligodendrocytes derived from iPSC-NSCs.
Appendix Figure S7	Colorimetric analysis of measurements of the NAD^+/NADH ratio in Detroit 551 control, WS5A and CP2A NSCs.

Appendix Table S1：**Summary of the ESC lines and iPSC clones used in the study.**

Cell type	Clone	Application	Source
ESC 1	-	Differentiation	hESC line 360
ESC 2	-	Differentiation	H1
Control iPSC 1	Clone 1	Differentiation and disease modelling	Detroit 551 fibroblasts
	Clone 2	Differentiation and disease modelling	Detroit 551 fibroblasts
	Clone 3	Differentiation and disease modelling	Detroit 551 fibroblasts
	Clone 4	Differentiation and disease modelling	Detroit 551 fibroblasts
Control iPSC 2	Clone 1	Differentiation and disease modelling	CCD-1079Sk fibroblasts
Control iPSC 3	Clone 1	Differentiation and disease modelling	AG05836 fibroblasts
WS5A iPSC	Clone 1	Differentiation and disease modelling	WS5A patient fibroblasts
	Clone 2	Differentiation and disease modelling	WS5A patient fibroblasts
	Clone 3	Differentiation and disease modelling	WS5A patient fibroblasts
CP2A iPSC	Clone 1	Differentiation and disease modelling	CP2A patient fibroblasts
	Clone 2	Differentiation and disease modelling	CP2A patient fibroblast

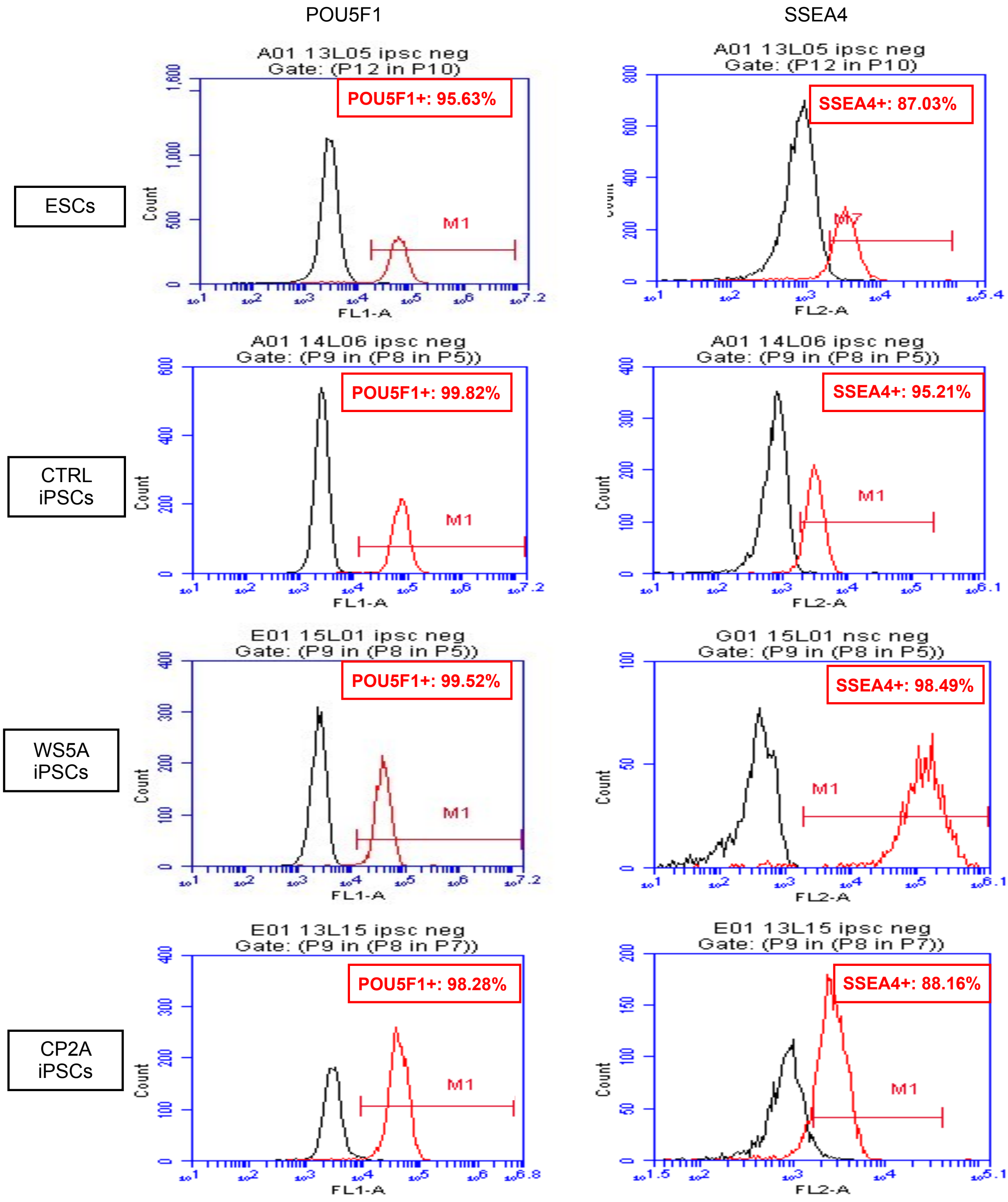

Appendix Figure S1：

Representative histograms from flow cytometric analysis of the positive cell population stained with pluripotency markers POU5F1 and SSEA4 in ESCs and iPSCs derived from Detroit 551 control, WS5A and CP2A fibroblasts.

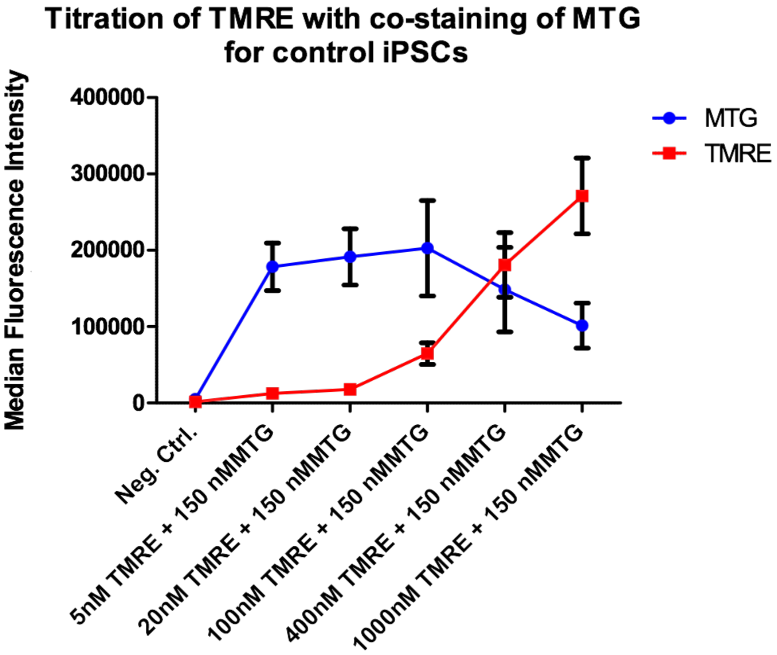

Appendix Figure S2:

Flow cytometric analysis of the correlation between MTG and TMRE expression under different titrations (from 5 nM to 1 µM) of TMRE using TMRE and 150 nM MTG dual staining of control iPSCs.

Data information: The data points represent iPSCs generated from one clone from Detroit 551 control. Data are presented as mean ± SEM for the number of samples (n=3, technical replicates).

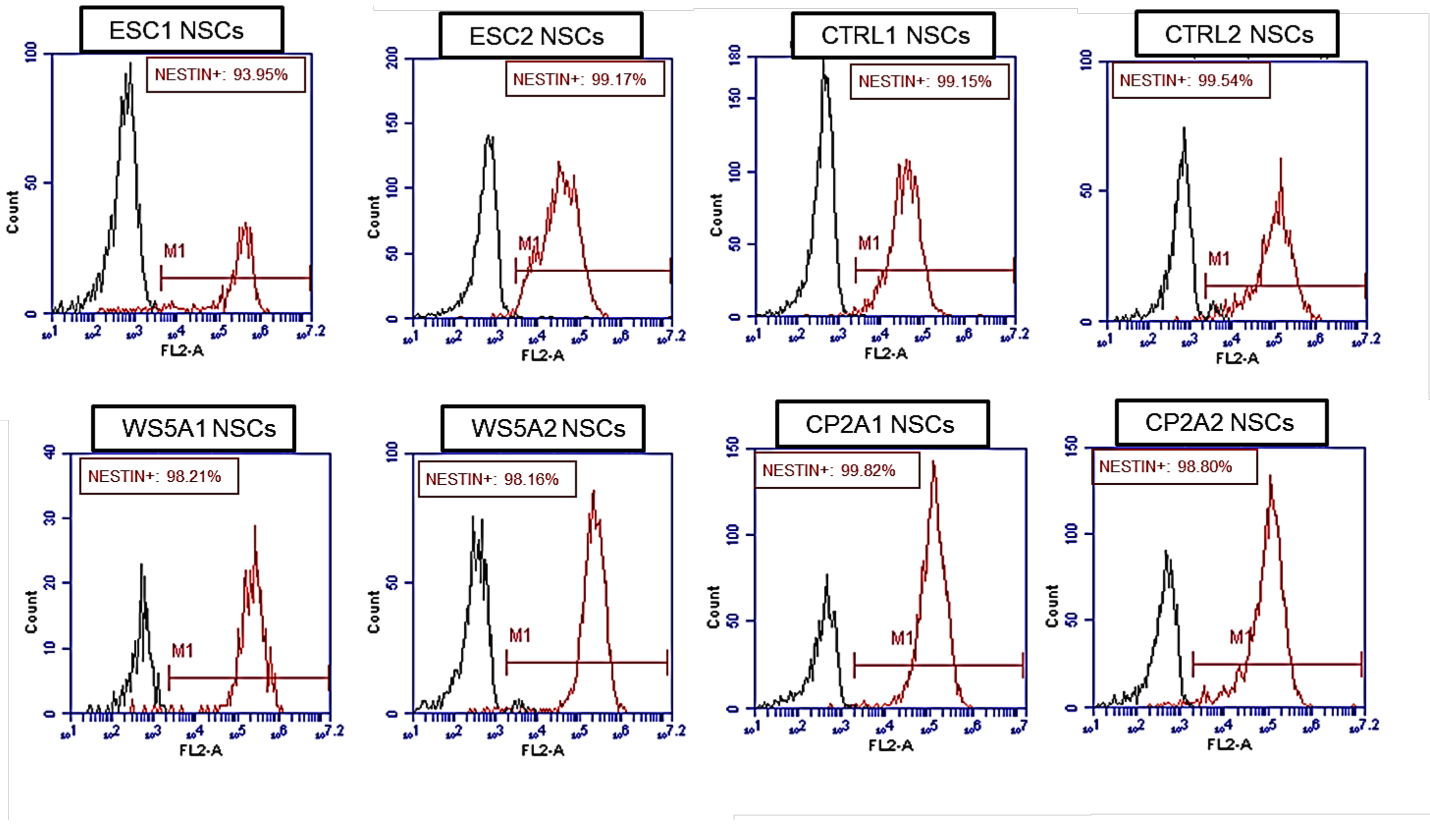

Appendix Figure S3:

Representative histograms of the positive staining with NSC marker NESTIN in NSCs derived from ESCs, Detroit 551 control, WS5A and CP2A iPSCs using flow cytometric analysis.

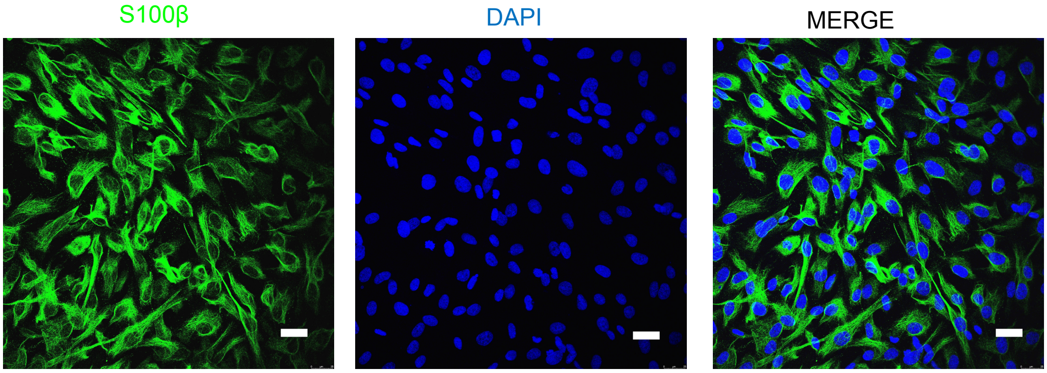

Appendix Figure S4：
Representative images of immunostaining for astrocyte marker S100β from astrocytes derived from iPSC-NSCs (scale bar, 50 μm). Nuclei are stained with DAPI (blue).

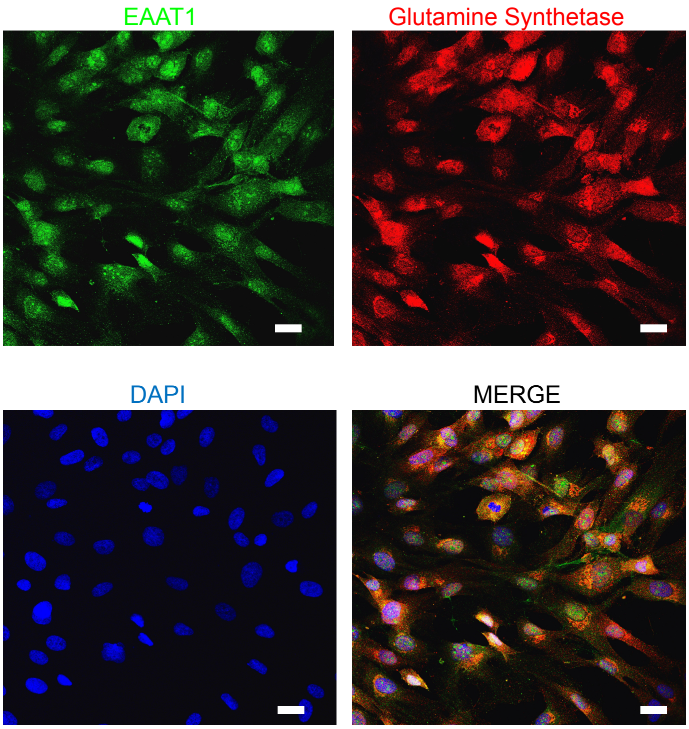

Appendix Figure S5:
Representative images of immunostaining for astrocyte markers EAAT1 and Glutamine Synthetase from astrocytes derived from iPSC-NSCs (scale bar, 50 μm). Nuclei are stained with DAPI (blue).

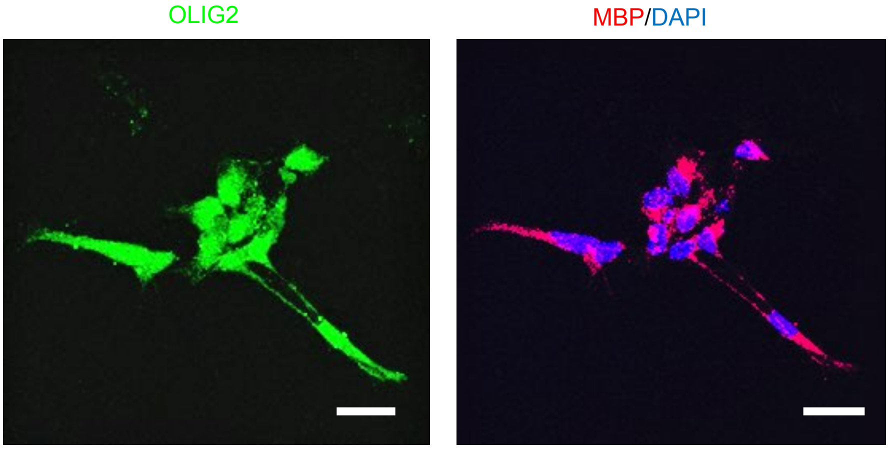

Appendix Figure S6：
Representative images of immunostaining for OLIG2 and MBP from oligodendrocytes derived from iPSC-NSCs (scale bar, 50 μm). Nuclei are stained with DAPI (blue).

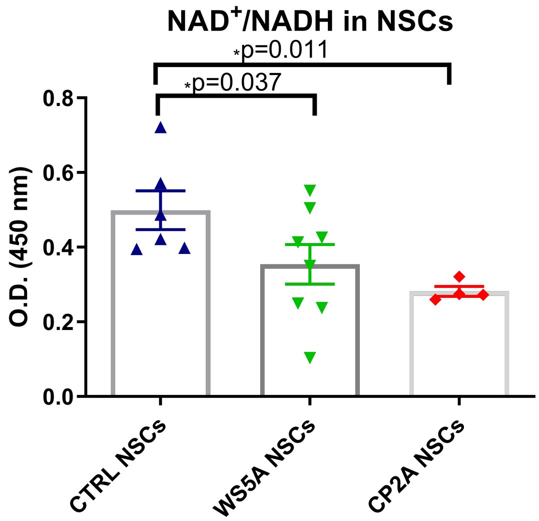

Appendix Figure S7：

Colorimetric analysis of measurements of the NAD^+/NADH ratio in Detroit 551 control, WS5A and CP2A NSCs

.

Data information: The data points represent NSCs generated from 2 or 3 different clones from Detroit 551 control, 3 different clones from WS5A patient and 2 different clones from CP2A patient. Data are presented as mean ± SEM for the number of samples (n=3, technical replicates per clone). Mann-Whitney U test is used. Significance is denoted for P values of less than 0.05. *P<0.05.

.
