# Supplementary material for: Disease‐specific phenotypes in iPSC‐derived neural stem cells with POLG mutations
Source: EMBO Mol Med. 2020 Aug 25;12(10):e12146. doi: 10.15252/emmm.202012146 (PMC7539330; doi:10.15252/emmm.202012146)
Supplement: Supplementary file 2 — Expanded View Figures PDF [file EMMM-12-e12146-s002.pdf]

Expanded View Figures

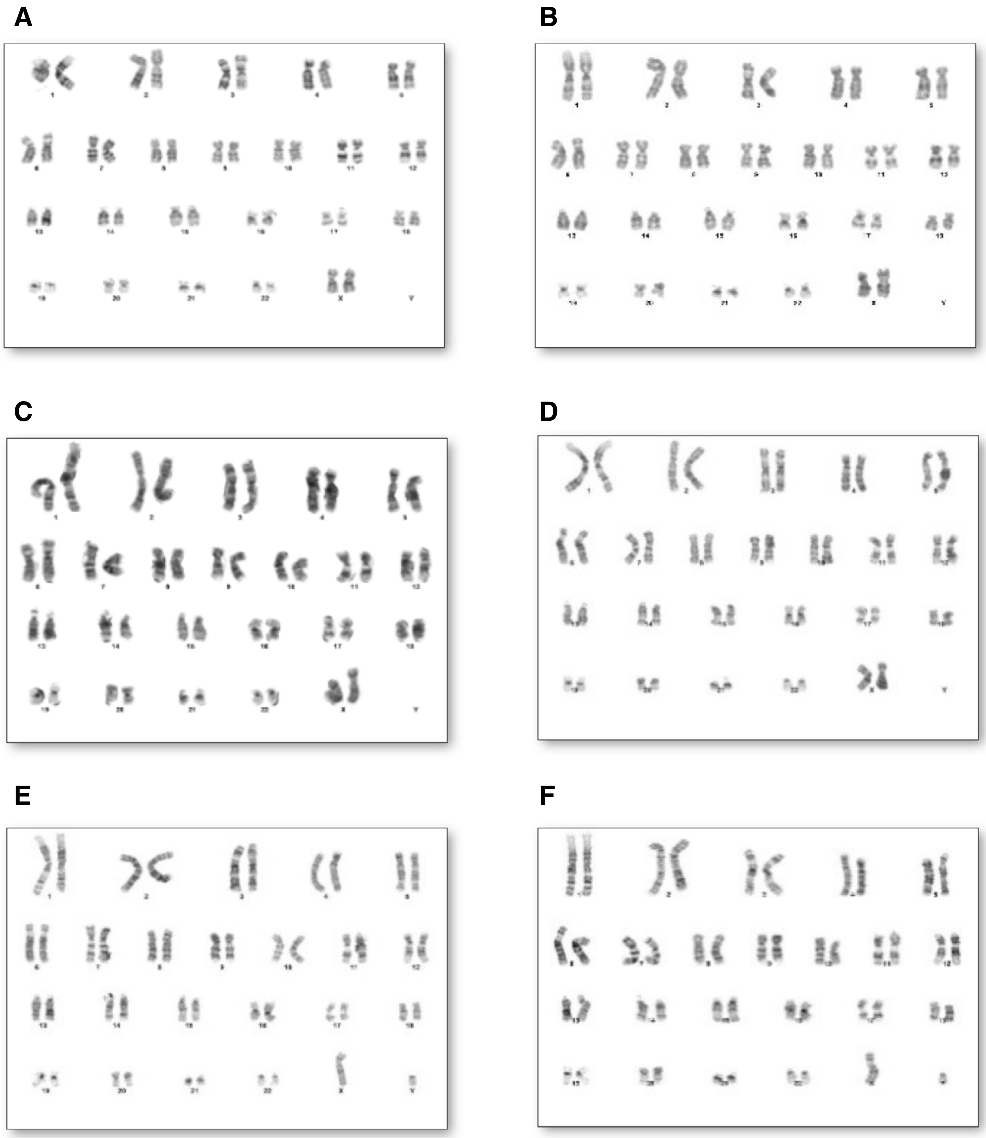

Figure EV1. Karyotype analysis for parental fibroblasts and reprogrammed iPSC lines.

A, C, E Representative karyotypes for Detroit 551 control fibroblasts (A) and WS5A and CP2A POLG fibroblasts (C, E).

B, D, F Representative karyotypes for control iPSC line (B) and POLG iPSC lines (D, F).

© 2020 The Authors

Figure EV2. Flow cytometric analysis of expression level of pluripotency markers TRA-1-60, TRA-1-81 and NANOG in iPSC lines.

A–C Flow cytometric analysis of expression level of pluripotency markers TRA-1-60 (A, $n = 3$, technical replicates per line/clone), TRA-1-81 (B, $n = 3$, technical replicates per line/clone), and NANOG (C, $n = 9$, technical replicates per line/clone for ESCs, control, and WS5A iPSCs; $n = 6$, technical replicates per clone for CP2A iPSCs) in ESC and iPSC lines. Data are demonstrated as individual clones (left panel, a) and combination as a group for ESCs, CTRL iPSCs, and WS5A and CP2A patient lines (right panel, b).

Data information: The data points in A–C represent 2 ESC lines, 2 different control clones from Detroit 551 iPSCs, 3 different iPSC clones from WS5A patient, and 2 different clones from CP2A patient iPSCs. Data are presented as mean ± SEM for the number of samples. Mann–Whitney *U*-test was used for the data presented in B, b. Two-sided Student's *t*-test was used for the data presented in A, b and C, b. Significance is denoted for *P* values of less than 0.05. $^{*}P < 0.05$; $^{**}P < 0.01$.
Source data are available online for this figure.

 © 2020 The Authors

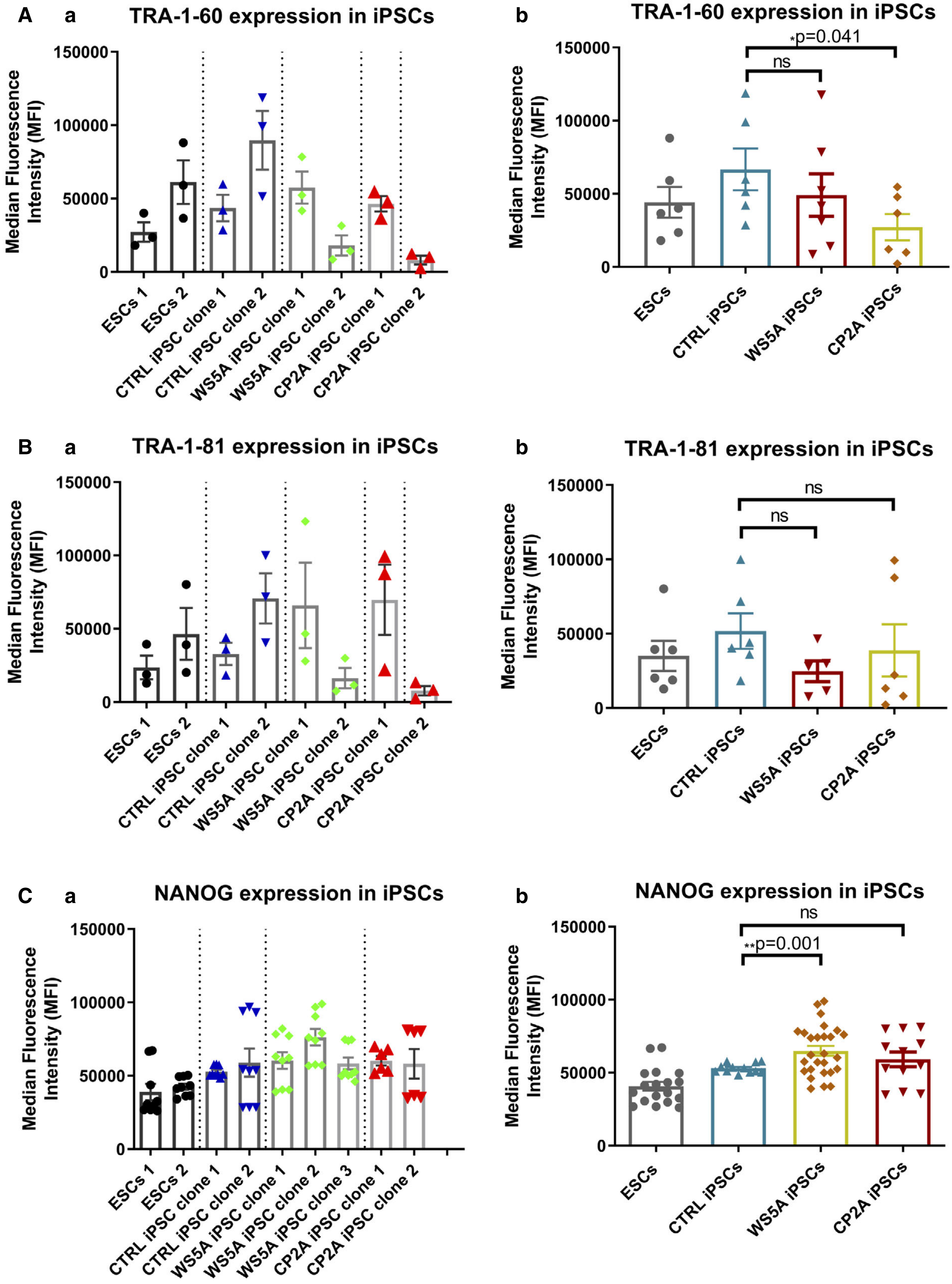
A
a
TRA-1-60 expression in iPSCs
Median Fluorescence Intensity (MFI)
ESCs 1
ESCs 2
CTRL iPSC clone 1
CTRL iPSC clone 2
WS5A iPSC clone 1
WS5A iPSC clone 2
CP2A iPSC clone 1
CP2A iPSC clone 2
b
TRA-1-60 expression in iPSCs
*p=0.041
ns
ESCs
CTRL iPSCs
WS5A iPSCs
CP2A iPSCs
B
a
TRA-1-81 expression in iPSCs
b
TRA-1-81 expression in iPSCs
ns
ns
C
a
NANOG expression in iPSCs
WS5A iPSC clone 3
b
NANOG expression in iPSCs
ns
**p=0.001

Figure EV2.

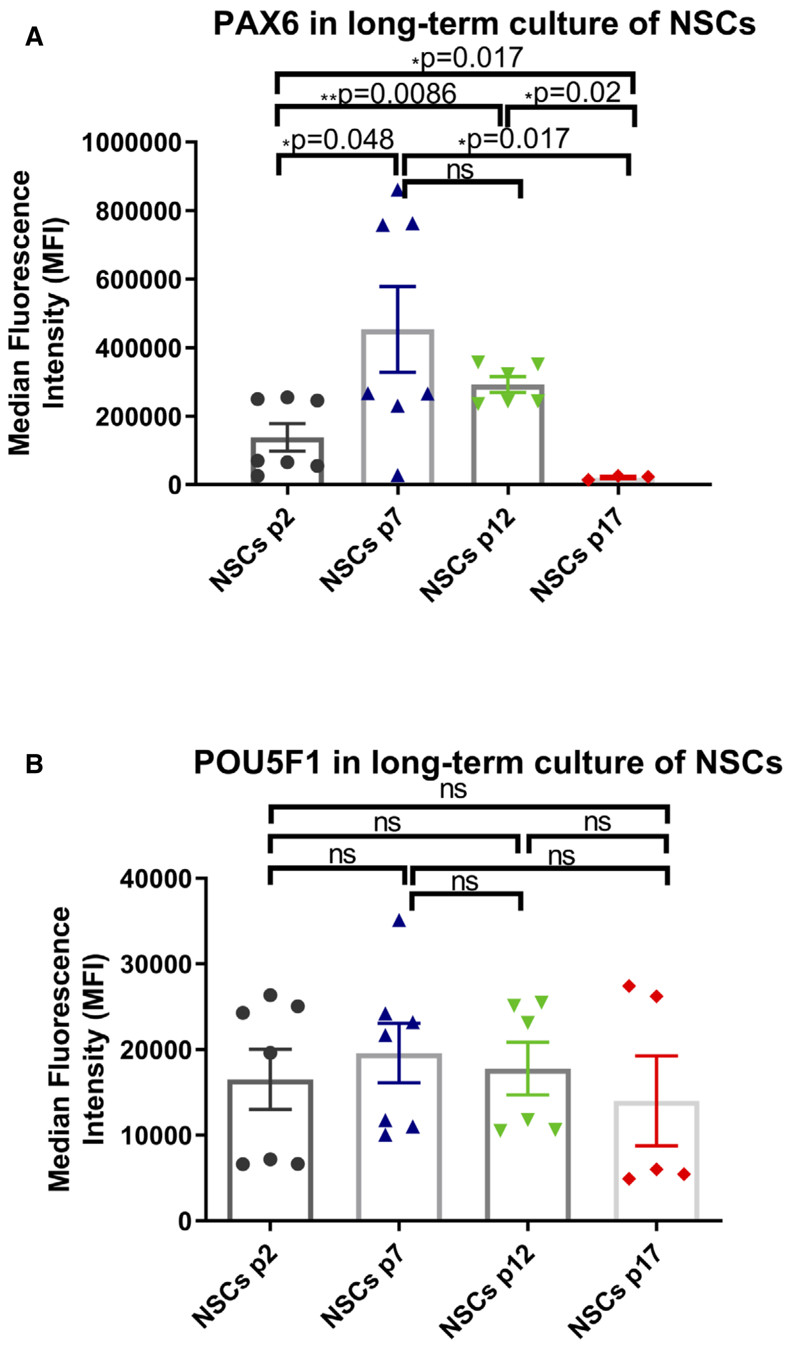

Figure EV3. Flow cytometric analysis of NSC marker PAX6 and pluripotency marker POU5F1 expression in long-term NSC culture.

A, B Representative bar graphs of NSC marker PAX6 (A, $n = 7$, technical replicates for p2; $n = 6$, technical replicates for p7, 12; $n = 3$, technical replicates for p17) and pluripotency marker POU5F1 (B, $n = 7$, technical replicates for p2; $n = 6$, technical replicates for p7, 12; $n = 3$, technical replicates for p17) in different passages during long-term NSC culture using flow cytometric analysis.

Data information: The data points in A and B represent NSCs generated from one Detroit 551 control iPSC line. Data are presented as mean ± SEM for the number of samples. Mann–Whitney *U*-test was used for the data presented in A and B. Significance is denoted for *P* values of less than 0.05. $^*P < 0.05$; $^{**}P < 0.01$.
Source data are available online for this figure.

 © 2020 The Authors

Figure EV4. Intracellular and mitochondrial ROS production in Detroit 551 control, WS5A, and CP2A fibroblasts, iPSCs, and NSCs.

A, B Intracellular ROS production measurements of the total ROS (DFCDA) level (A, n = 7, technical replicates per clone for control; n = 5, technical replicates per clone for WS5A; n = 8, technical replicates per clone for CP2A) and specific ROS level (B, n = 7, technical replicates per clone for control; n = 5, technical replicates per clone for WS5A; n = 8, technical replicates per clone for CP2A) calculated by total DCFDA/MTDR in iPSCs.

C, D Total intracellular ROS (C, n = 6, technical replicates for control; n = 4, technical replicates for WS5A; n = 5, technical replicates for CP2A) and specific ROS (D, n = 6, technical replicates for control; n = 4, technical replicates for WS5A; n = 5, technical replicates for CP2A) production measurements in fibroblasts.

E, F Intracellular ROS production measurements of the total ROS level (E, n = 6, technical replicates per clone for control; n = 5, technical replicates per clone for WS5A; n = 4, technical replicates per clone for CP2A) and mitochondrial ROS level (F, n = 6, technical replicates per clone for control; n = 5, technical replicates per clone for WS5A; n = 4, technical replicates per clone for CP2A) in NSCs.

Data information: The data points in A, B and F represent iPSCs and NSCs generated from 3 different control clones including 2 different clones from Detroit 551 control, 3 different clones from WS5A patient, and 2 different clones from CP2A patient. The data points in E represent NSCs generated from 4 clones from Detroit 551 control and one clone from control AG05836, 3 clones from WS5A patient, and 2 clones from CP2A patient. Data are presented as mean ± SEM for the number of samples. Mann–Whitney U-test was used for the data presented in A–C and E. Two-sided Student's t-test was used for the data presented in D and F. Significance is denoted for P values of less than 0.05. *$P < 0.05$; **$P < 0.01$; ****$P < 0.0001$.
Source data are available online for this figure.

© 2020 The Authors

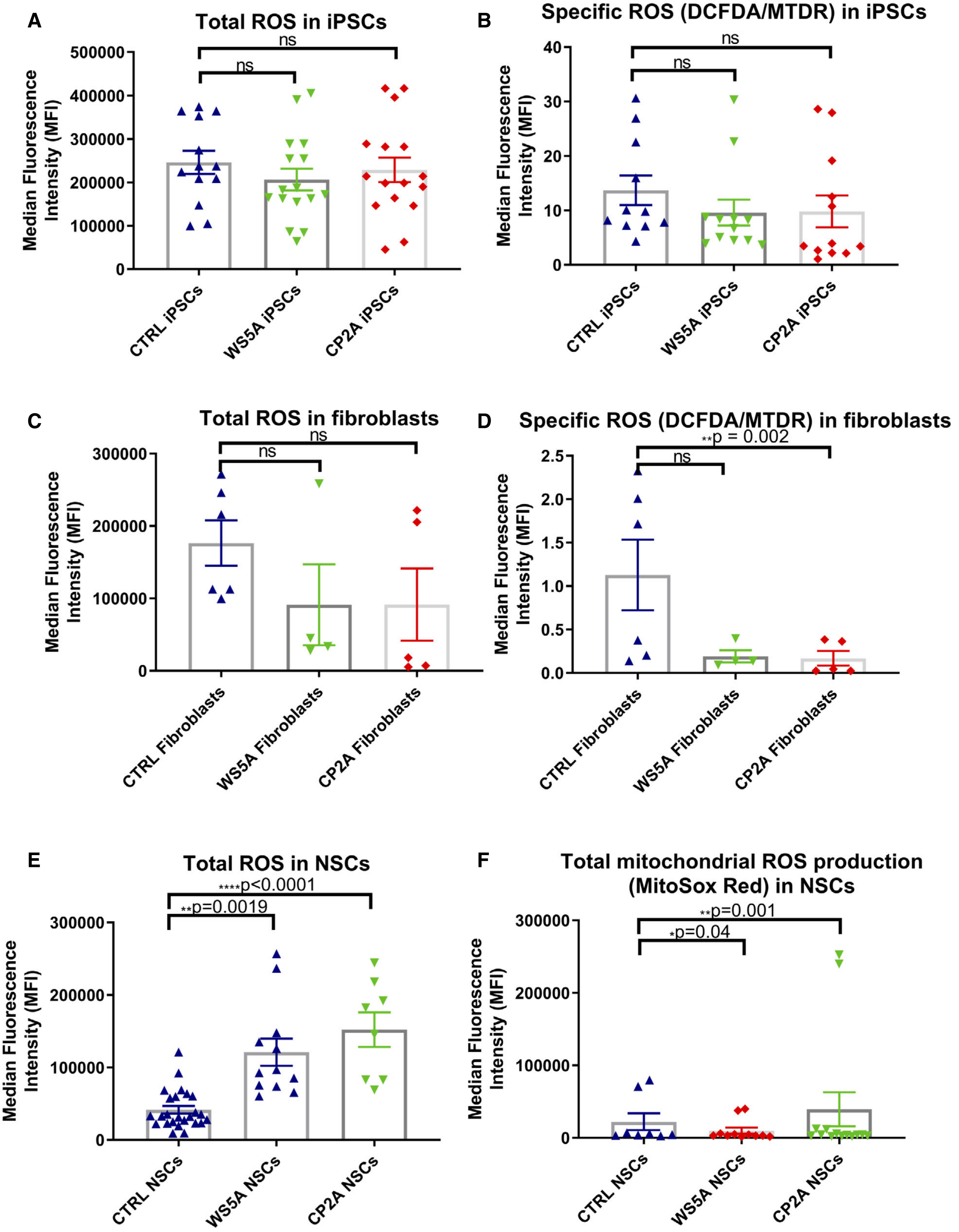

Figure EV4.

© 2020 The Authors

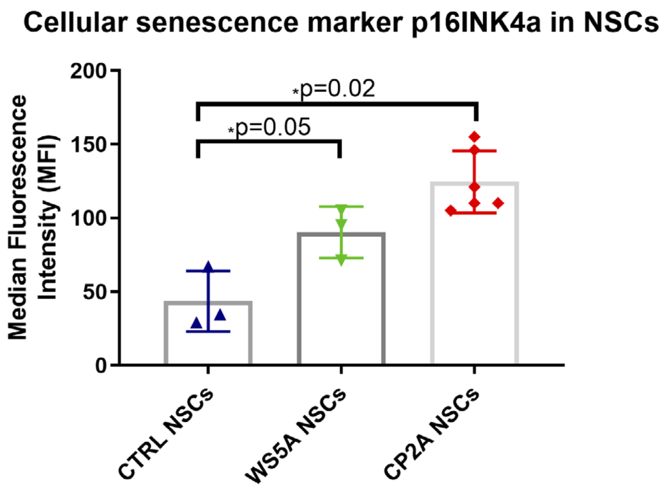

Figure EV5. Flow cytometric analysis of the expression level of cellular senescence marker p16INK4a in NSCs.

The data points represent NSCs generated from one clone from Detroit 551 control, one clone from WS5A patient, and 2 different clones from CP2A patient. Data are presented as mean ± SEM for the number of samples (n = 3, technical replicates per clone). Mann–Whitney U-test was used for the data presented. Significance is denoted for P values of less than 0.05. $^{*}P < 0.05$.

Source data are available online for this figure.
