# Supplementary material for: Disease‐specific phenotypes in iPSC‐derived neural stem cells with POLG mutations
Source: EMBO Mol Med. 2020 Aug 25;12(10):e12146. doi: 10.15252/emmm.202012146 (PMC7539330; doi:10.15252/emmm.202012146)

Appendix Figure S5 EAAT1

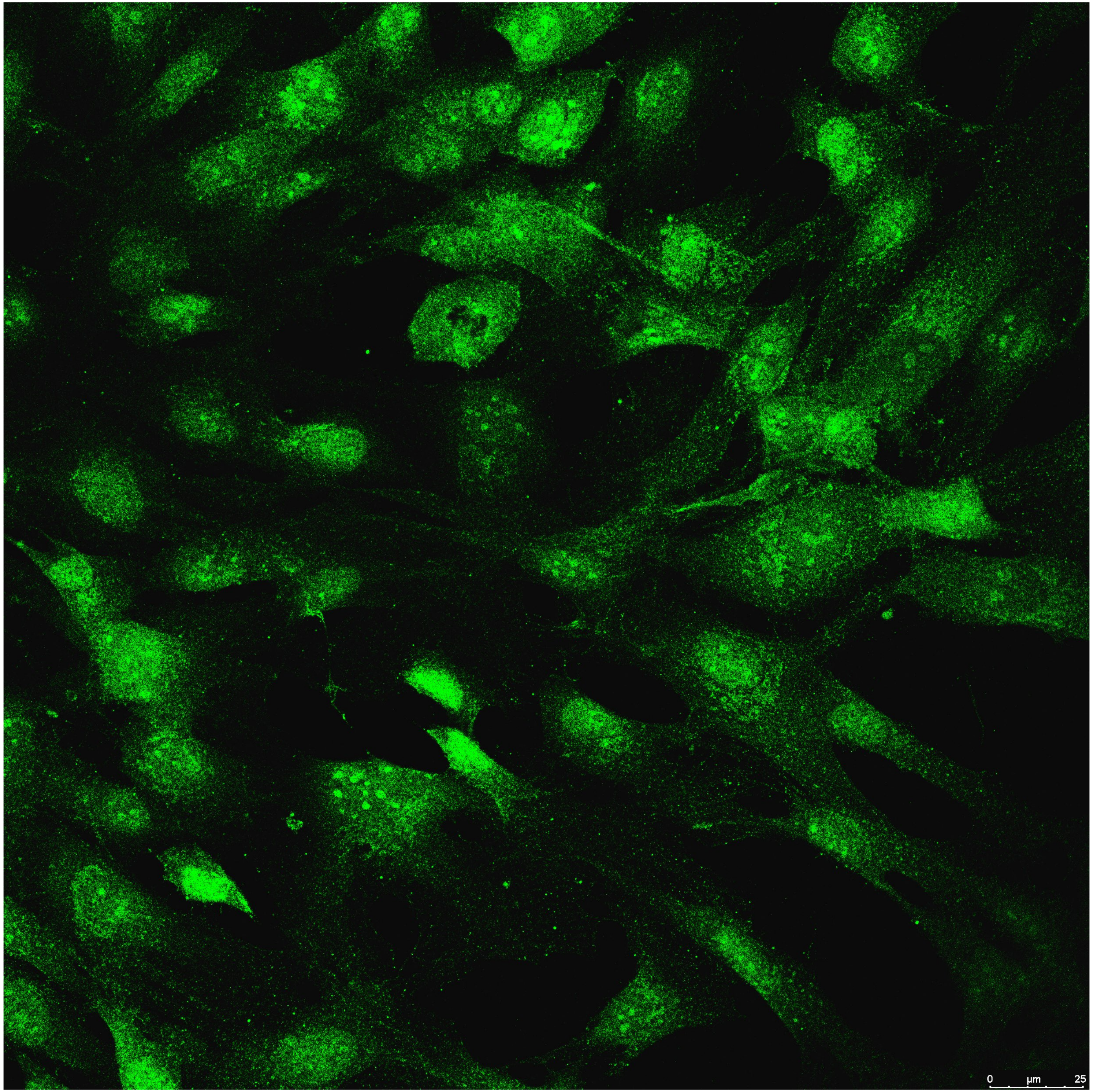

Appendix Figure S5 Glutamine Synthetase

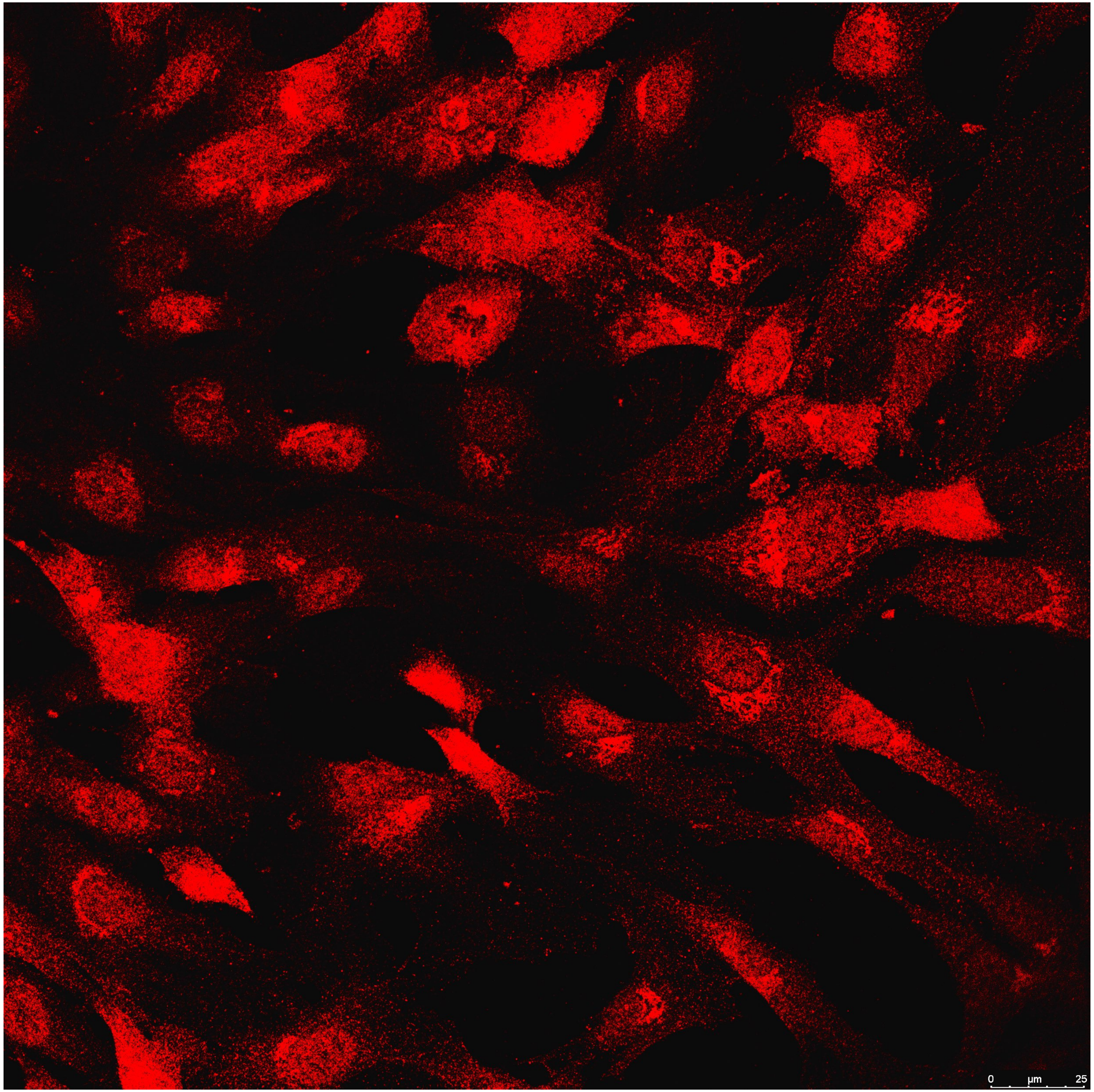

Appendix Figure S5 DAPI

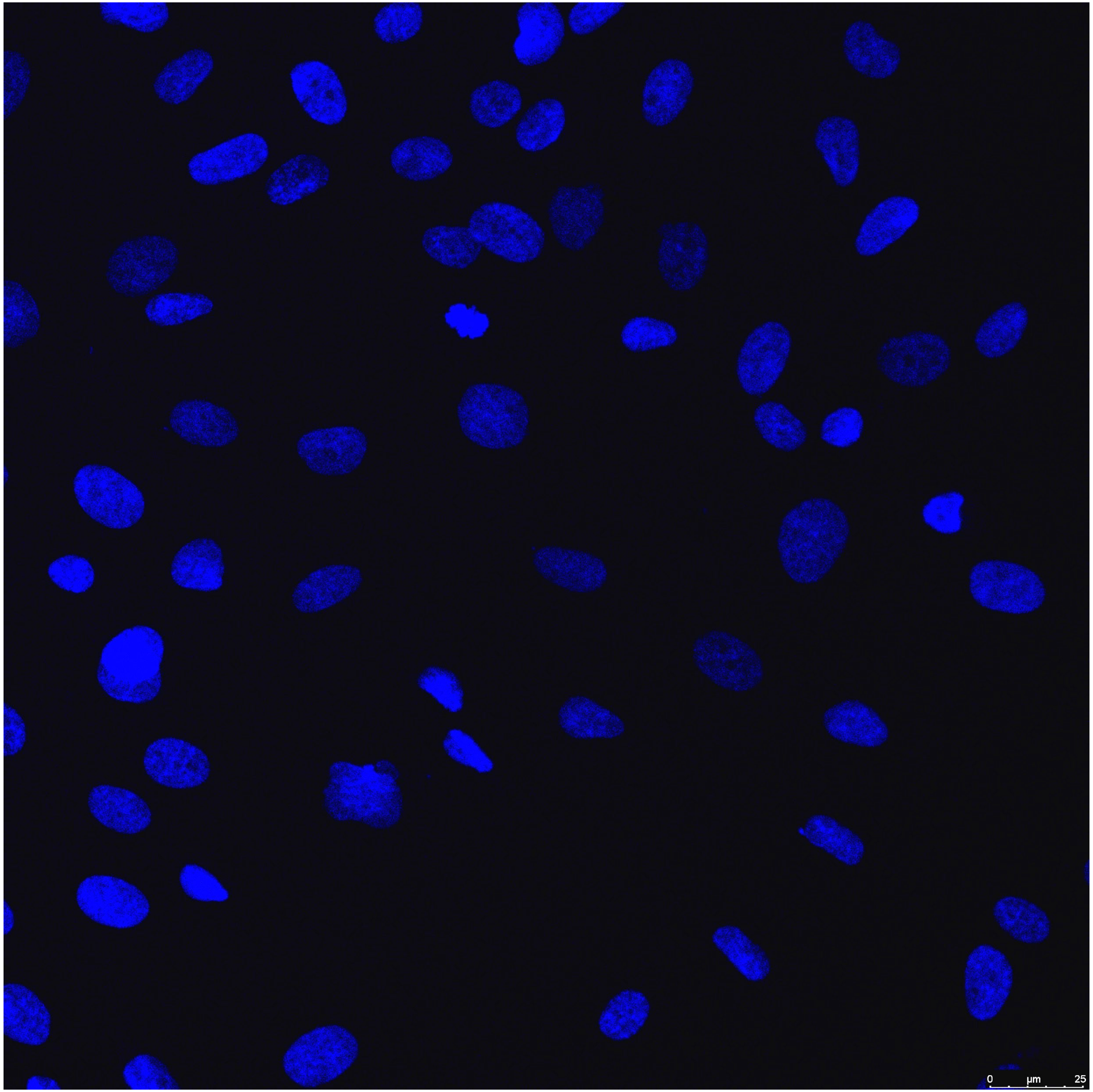

Appendix Figure S5 MERGE

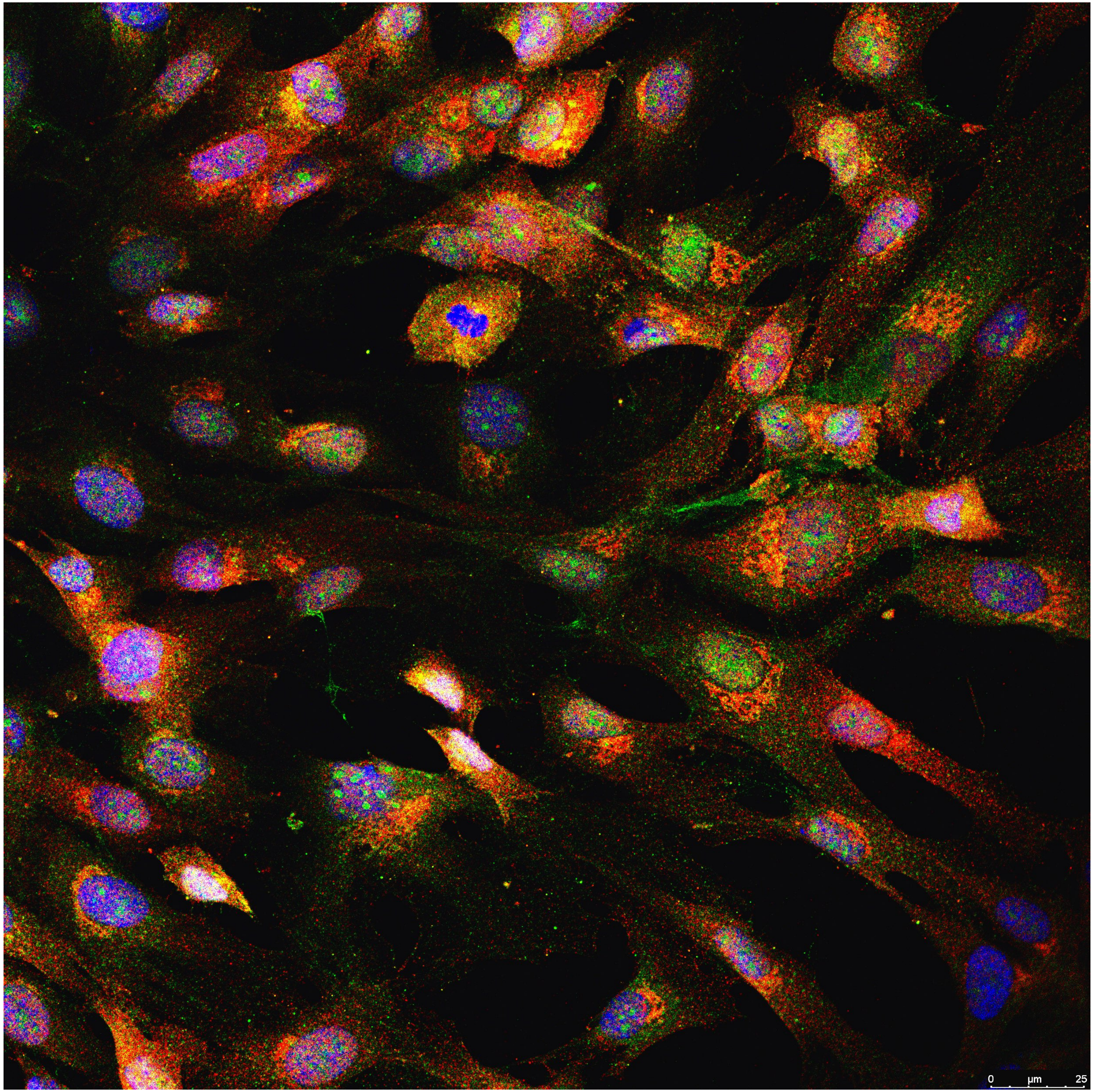

Supplement: Supplementary file 3 — Source Data for Expanded View and Appendix [file EMMM-12-e12146-s011.zip › EMM-2020-12146-V5_Source data_Appendix Figure S5/EMM-2020-12146-V5_Source data_Appendix Figure S5.pdf]
