# Supplementary figures and images for: Disease‐specific phenotypes in iPSC‐derived neural stem cells with POLG mutations
Source: EMBO Mol Med. 2020 Aug 25;12(10):e12146. doi: 10.15252/emmm.202012146 (PMC7539330; doi:10.15252/emmm.202012146)

Appendix Figure S4 S100 $\beta$

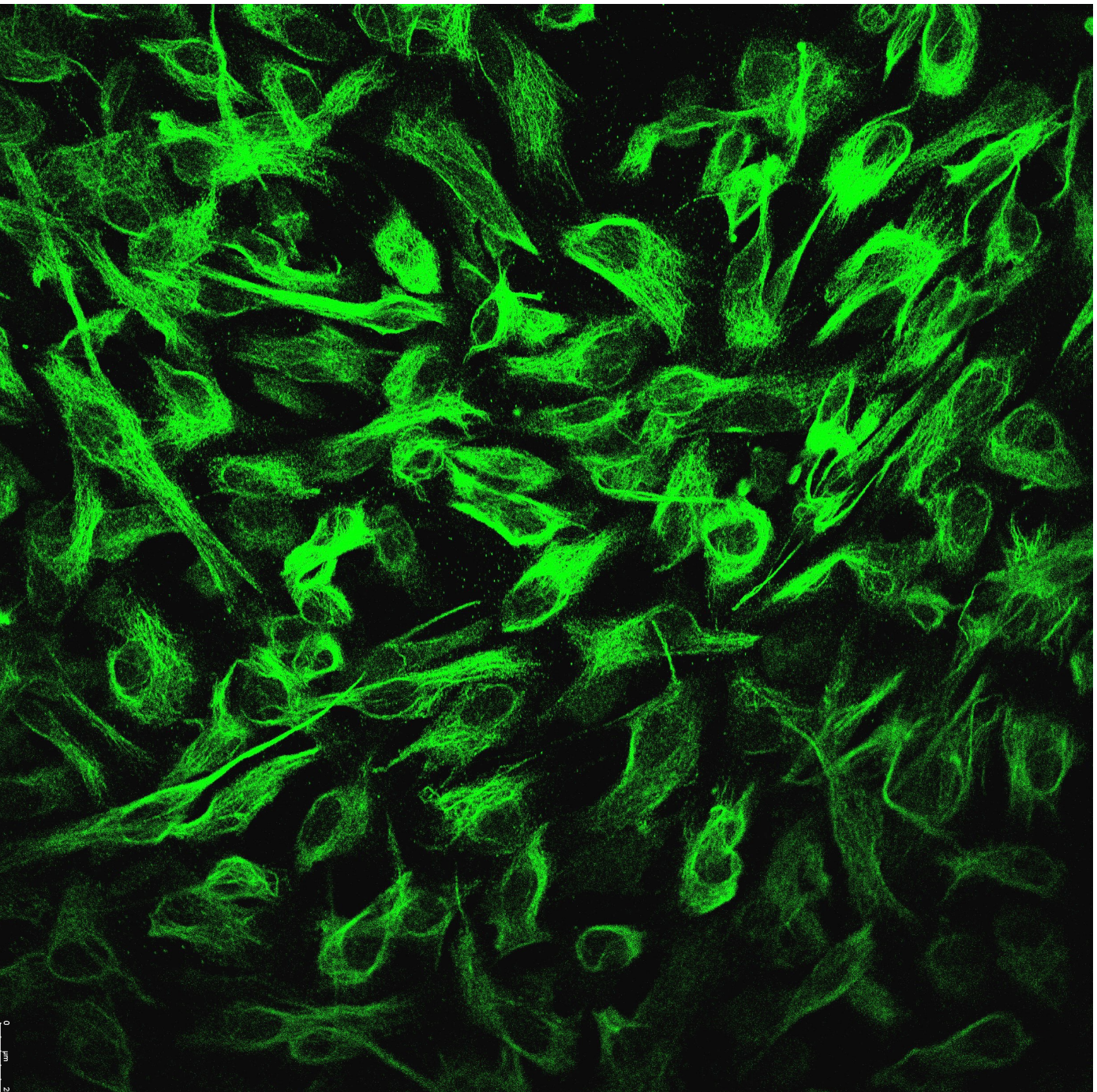

Appendix Figure S4 DAPI

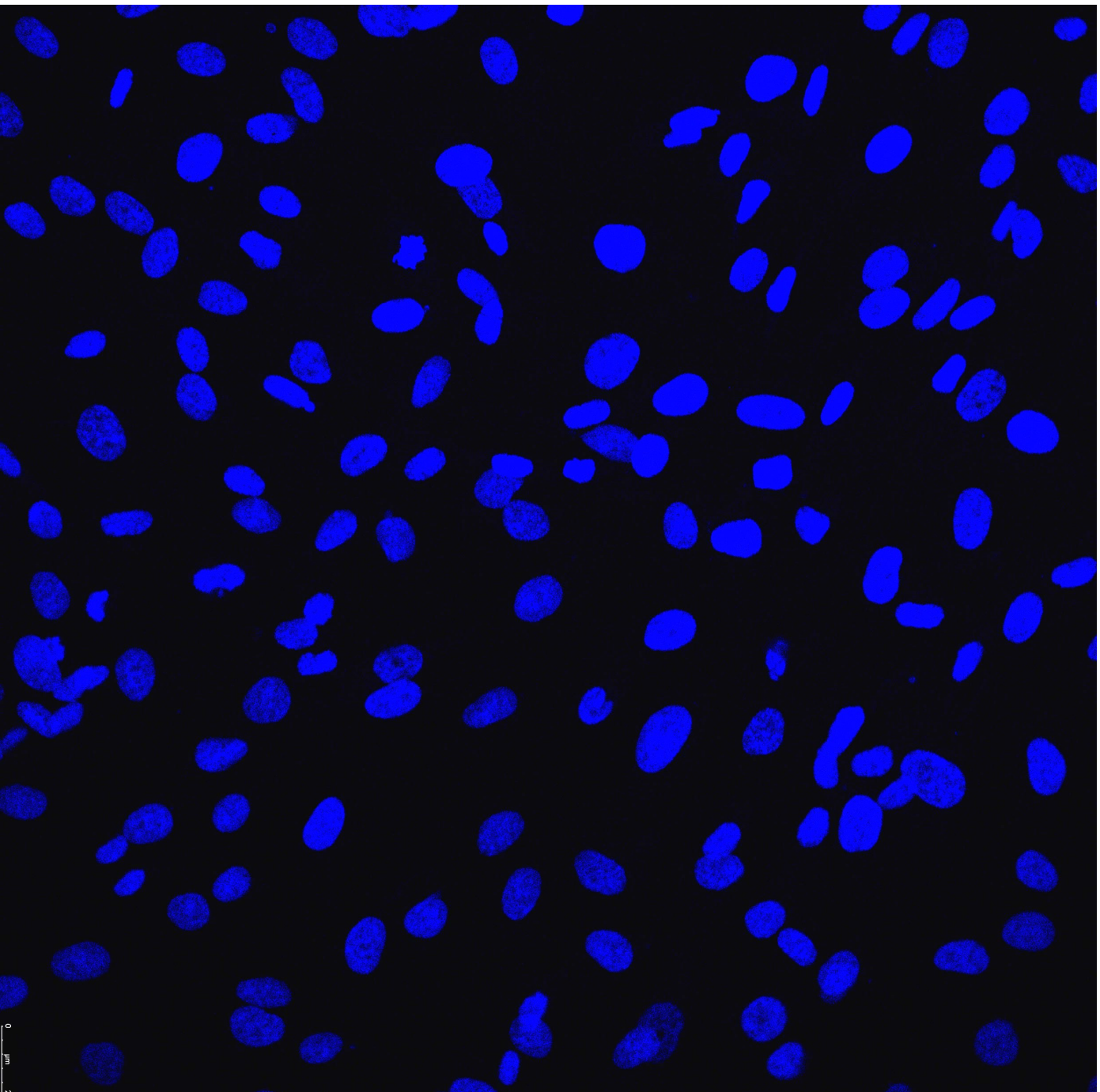

Appendix Figure S4 MERGE

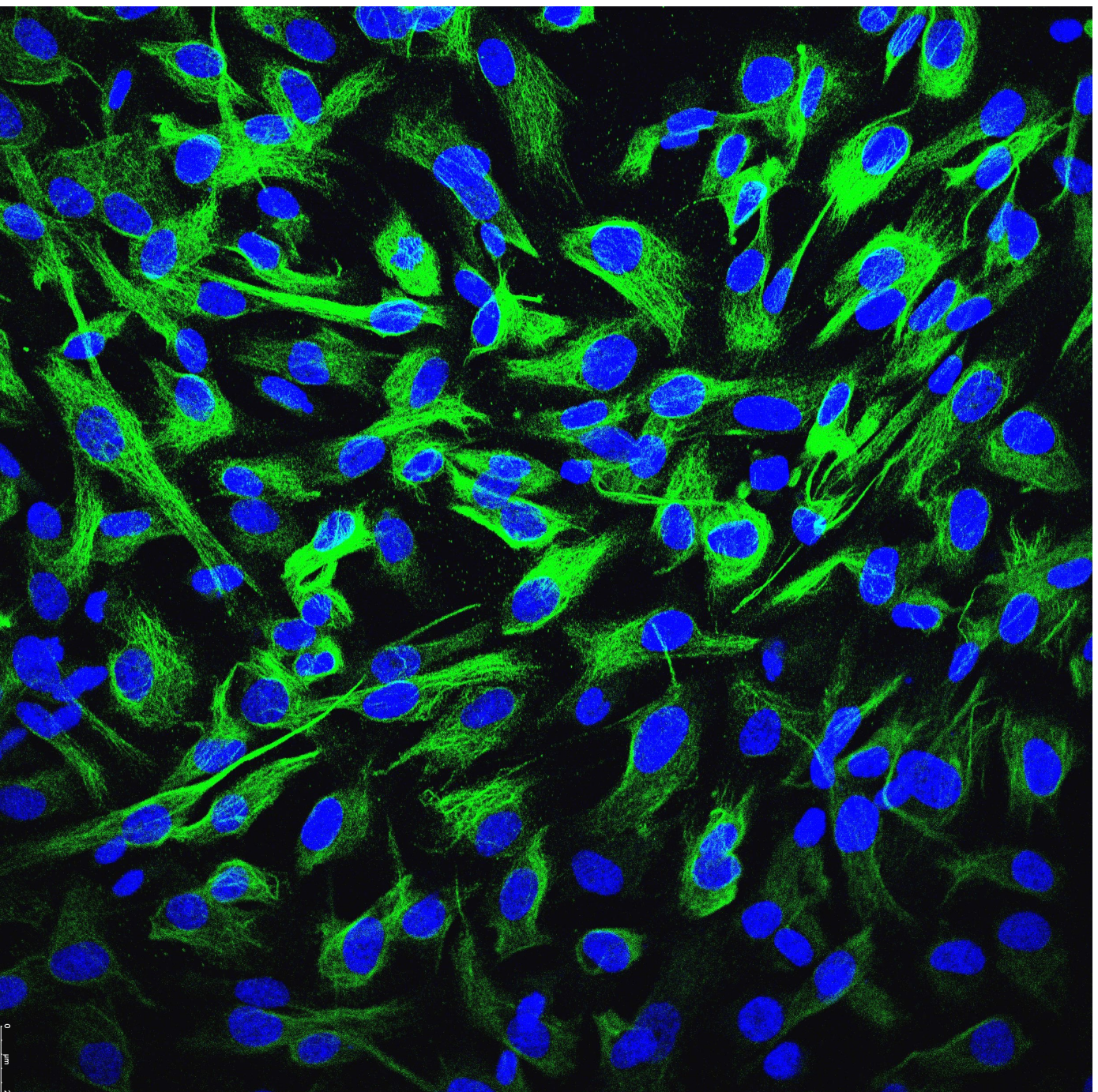

Supplement: Supplementary file 3 — Source Data for Expanded View and Appendix [file EMMM-12-e12146-s011.zip › EMM-2020-12146-V5_Source data_Appendix Figure S4/EMM-2020-12146-V5_Source data_Appendix Figure S4.pdf]

Fig. 4E, a astrocytes- GFAP/DAPI

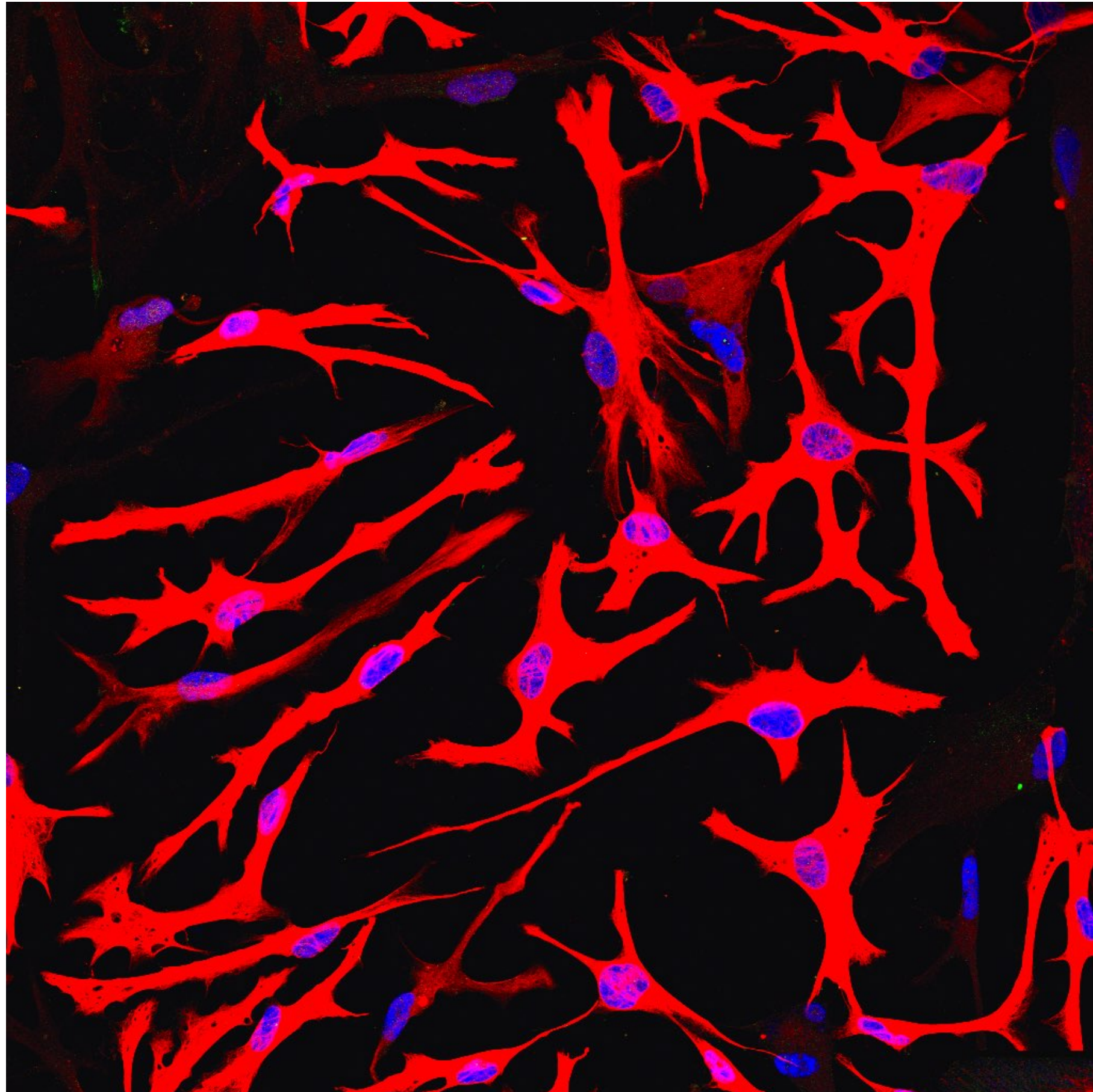

Fig. 4E, b Oligodendrocytes - GALC/DAPI

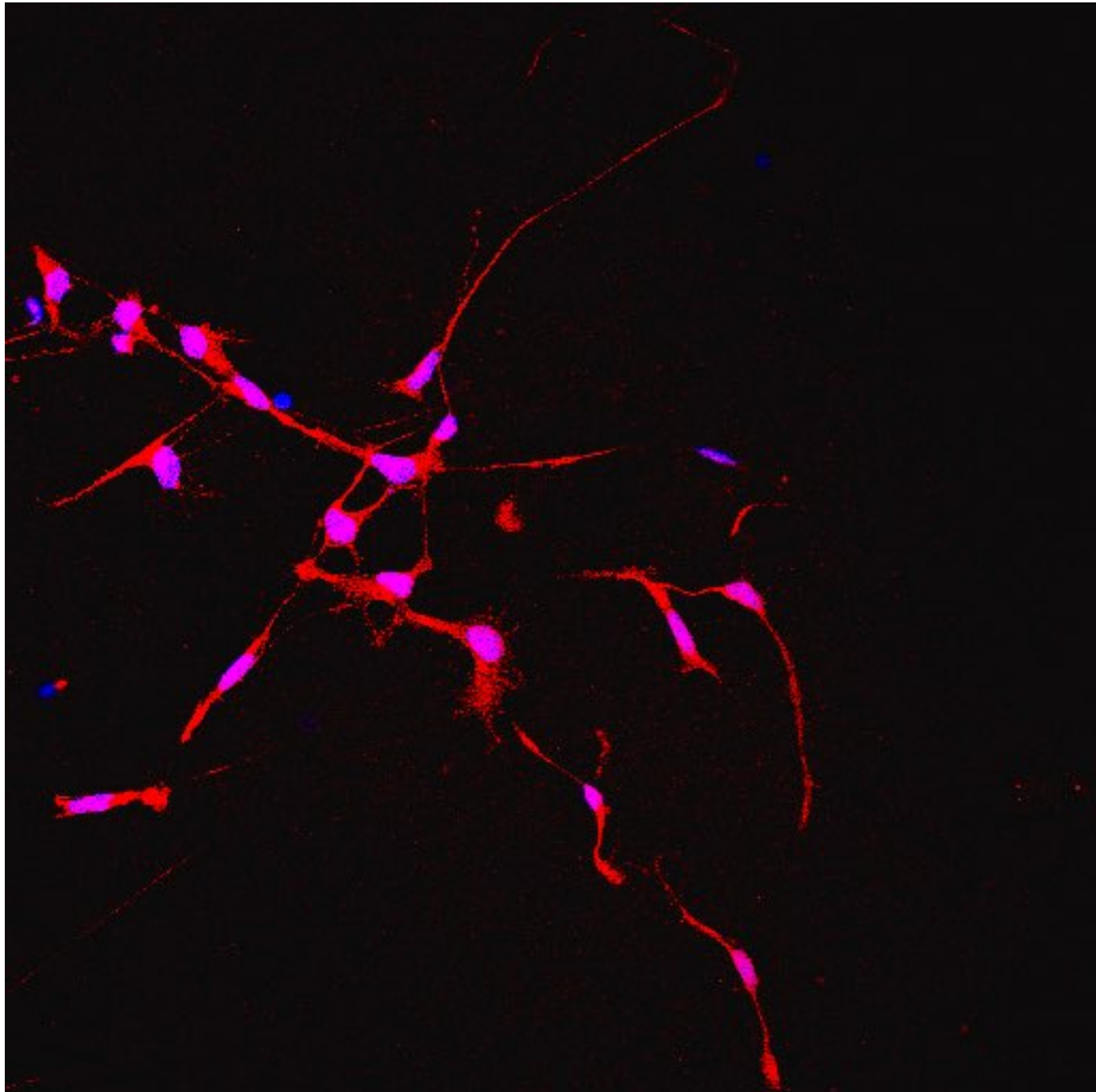

Fig. 4E, c NEURON- TH/TUJ1/DAPI

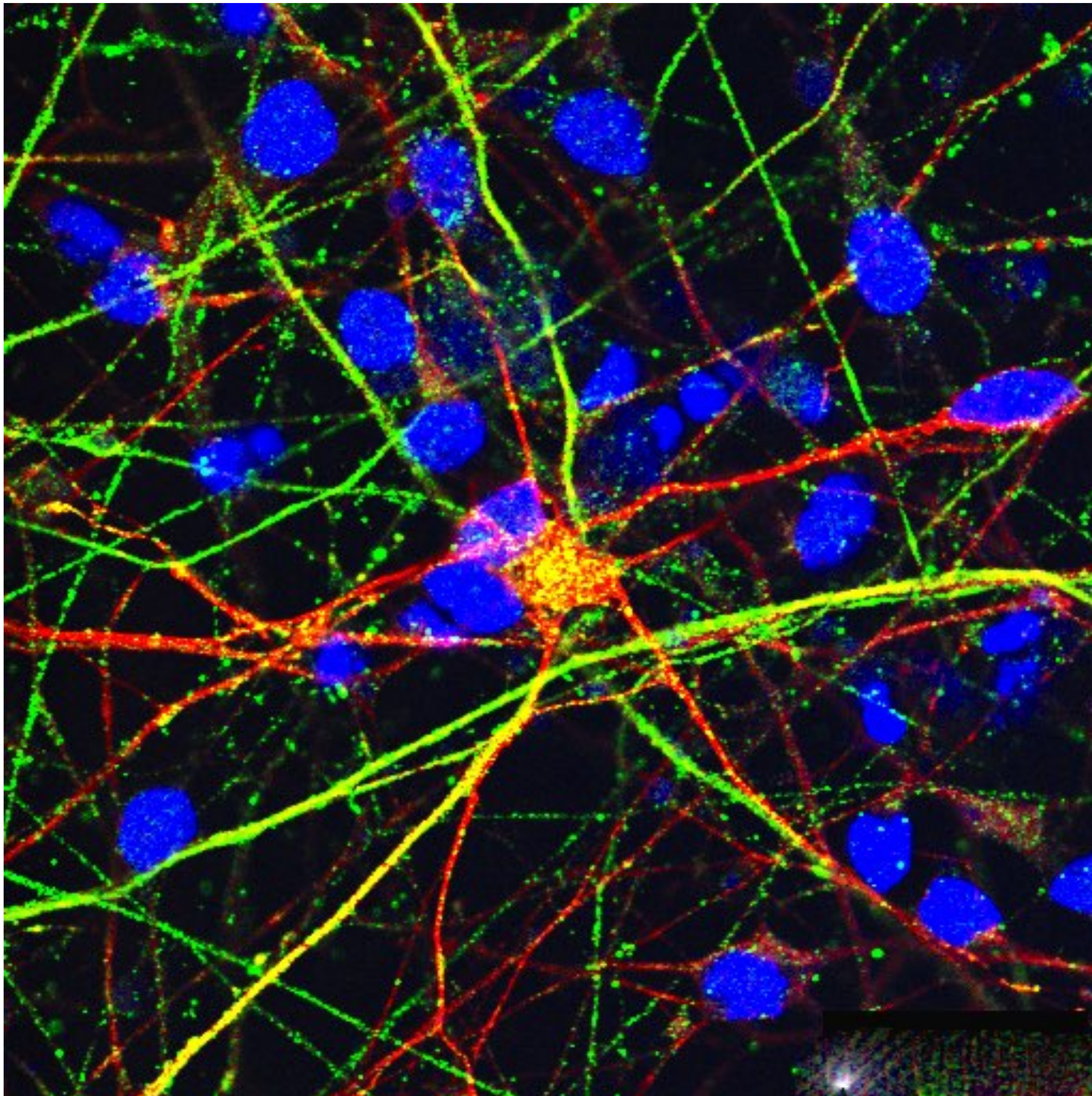

Supplement: Supplementary file 8 — Source Data for Figure 4 [file EMMM-12-e12146-s006.zip › EMM-2020-12146-V5_Source data_Images_Figure 4.pdf]

Fig. 5A, CTRL NSC

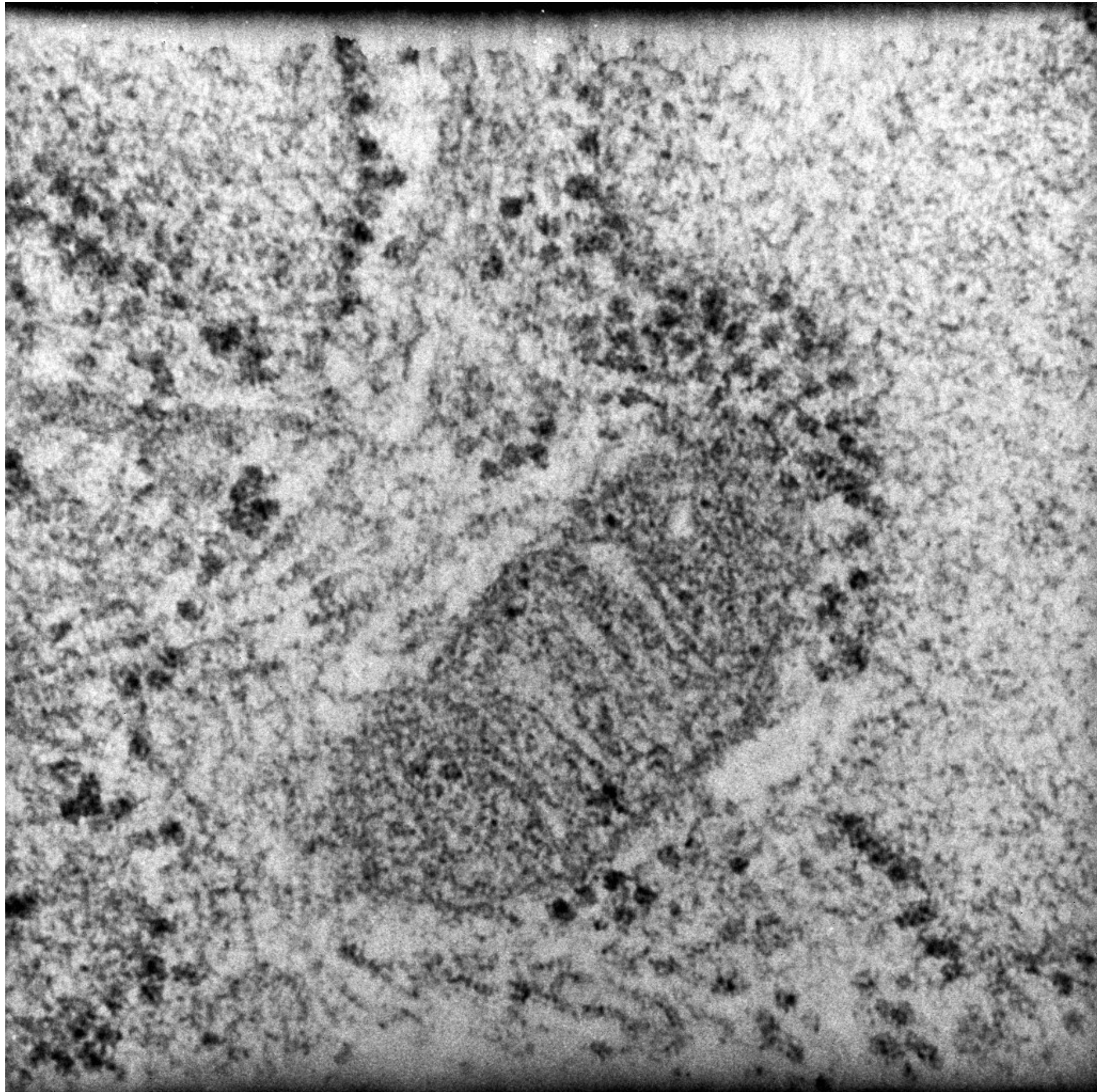

Fig. 5A, WS5A NSC

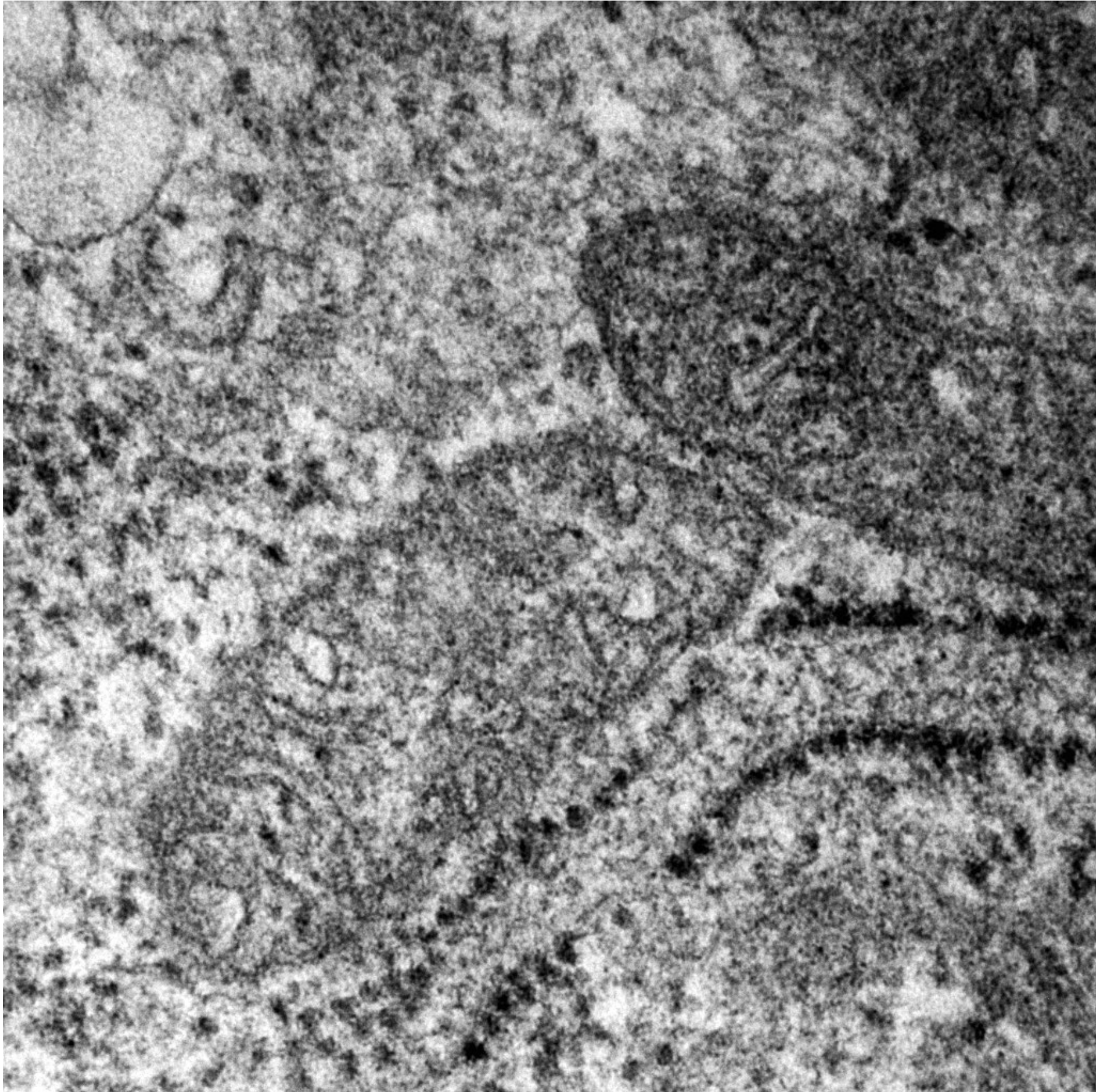

Fig. 5A, CP2A NSC

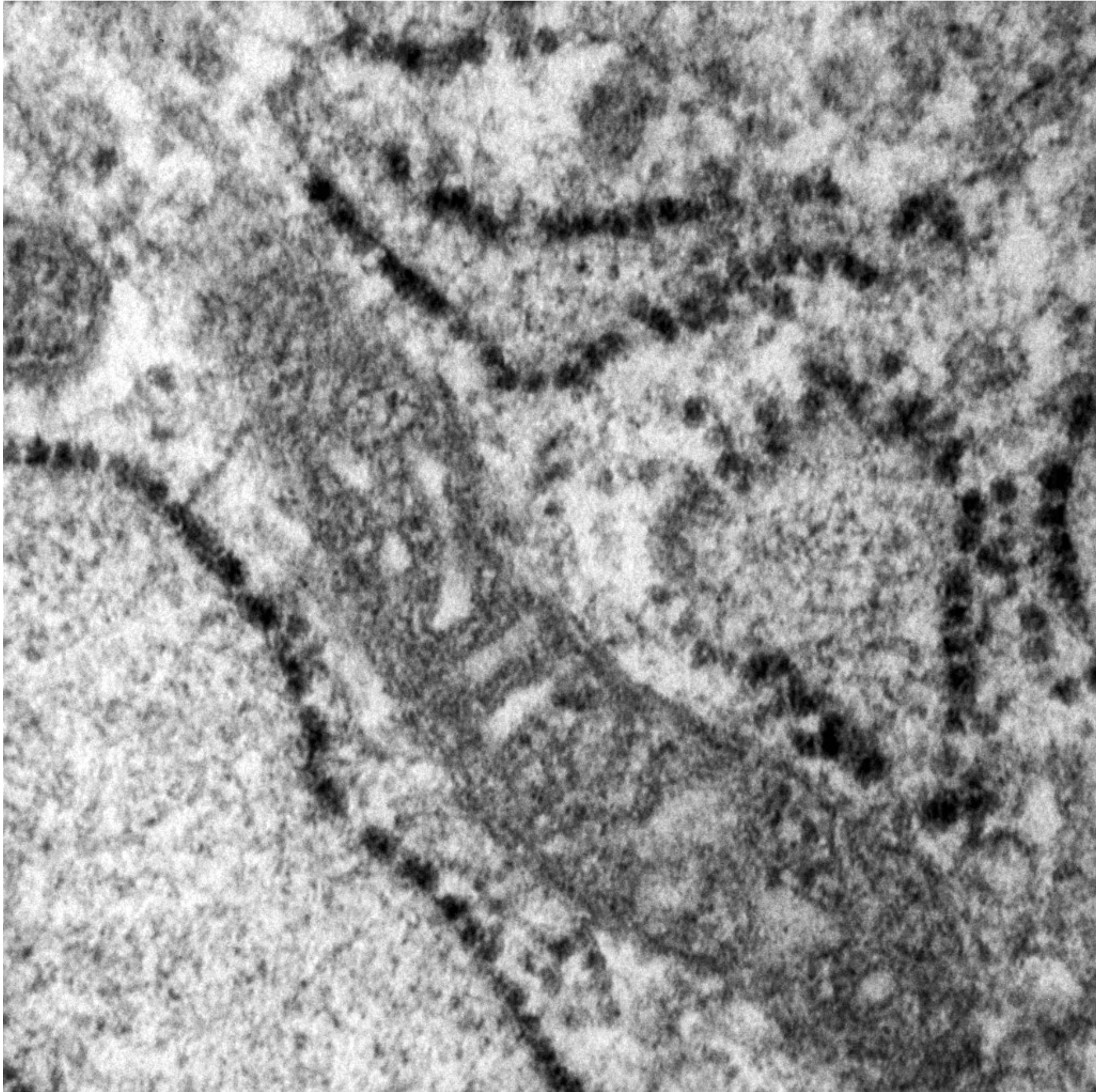

Supplement: Supplementary file 9 — Source Data for Figure 5 [file EMMM-12-e12146-s007.zip › EMM-2020-12146-V5_Source data_Images_Figure 5.pdf]

Fig. 6G, Long-PCR

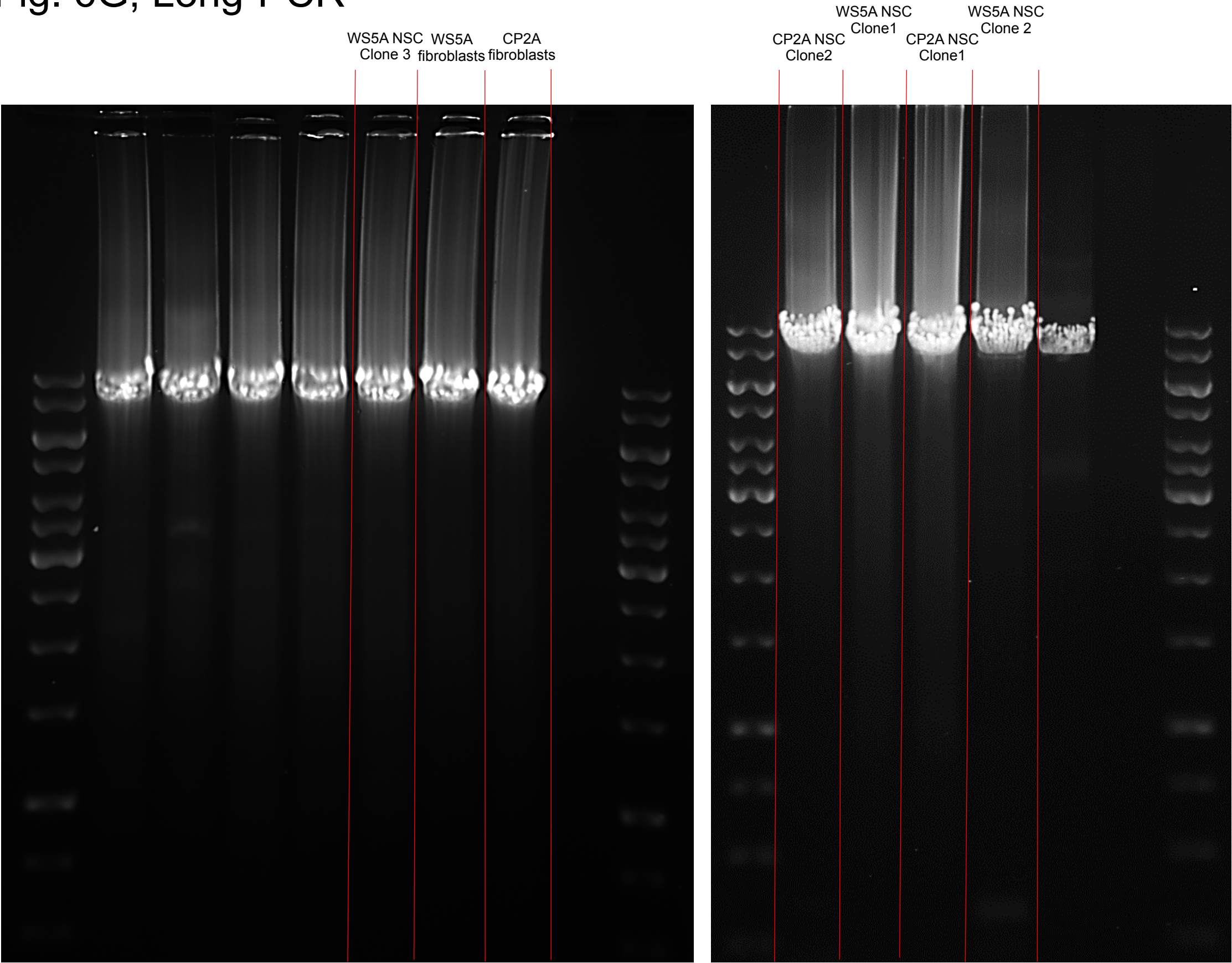

Fig. 6H DAPI

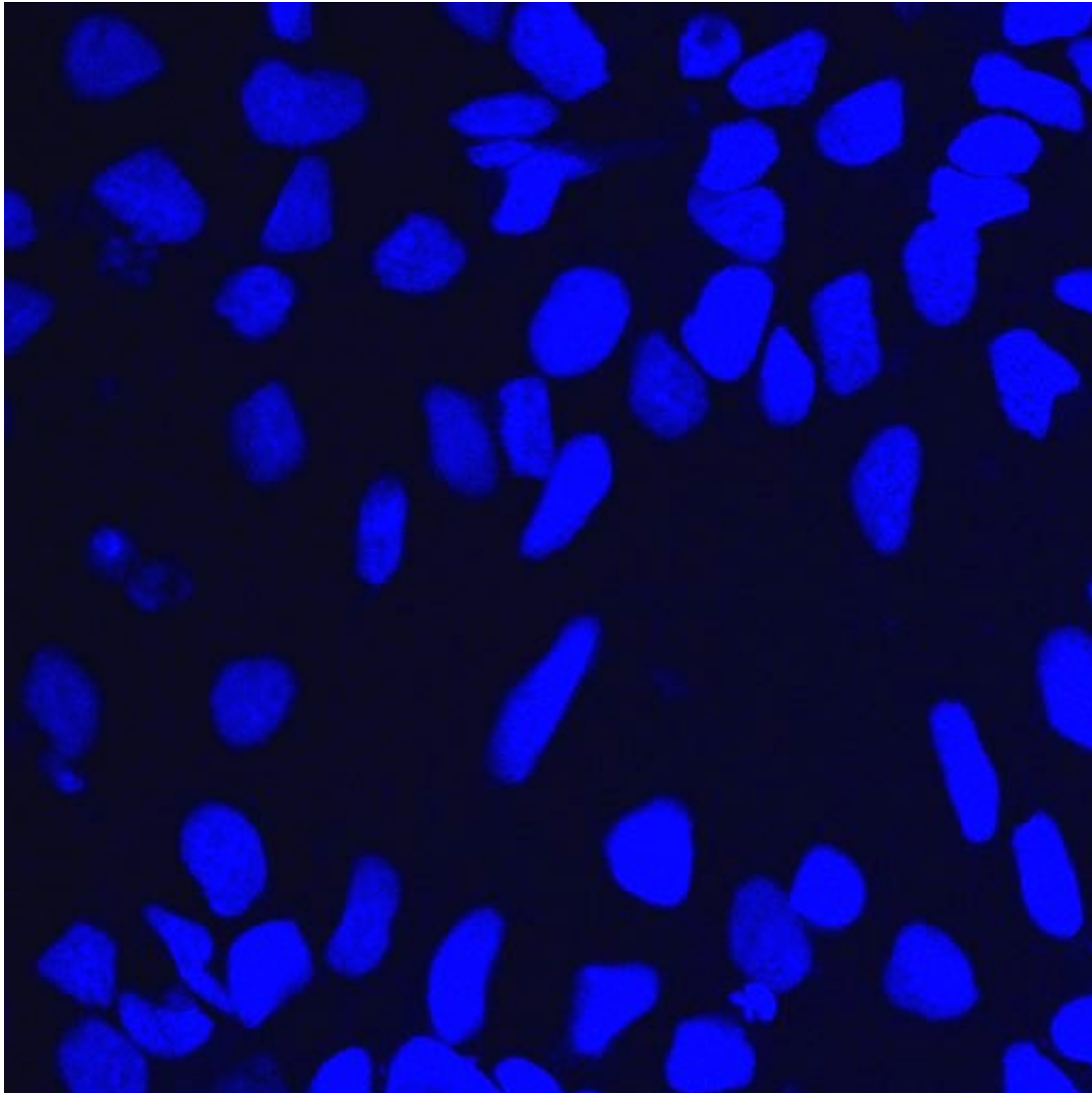

Fig. 6H TH

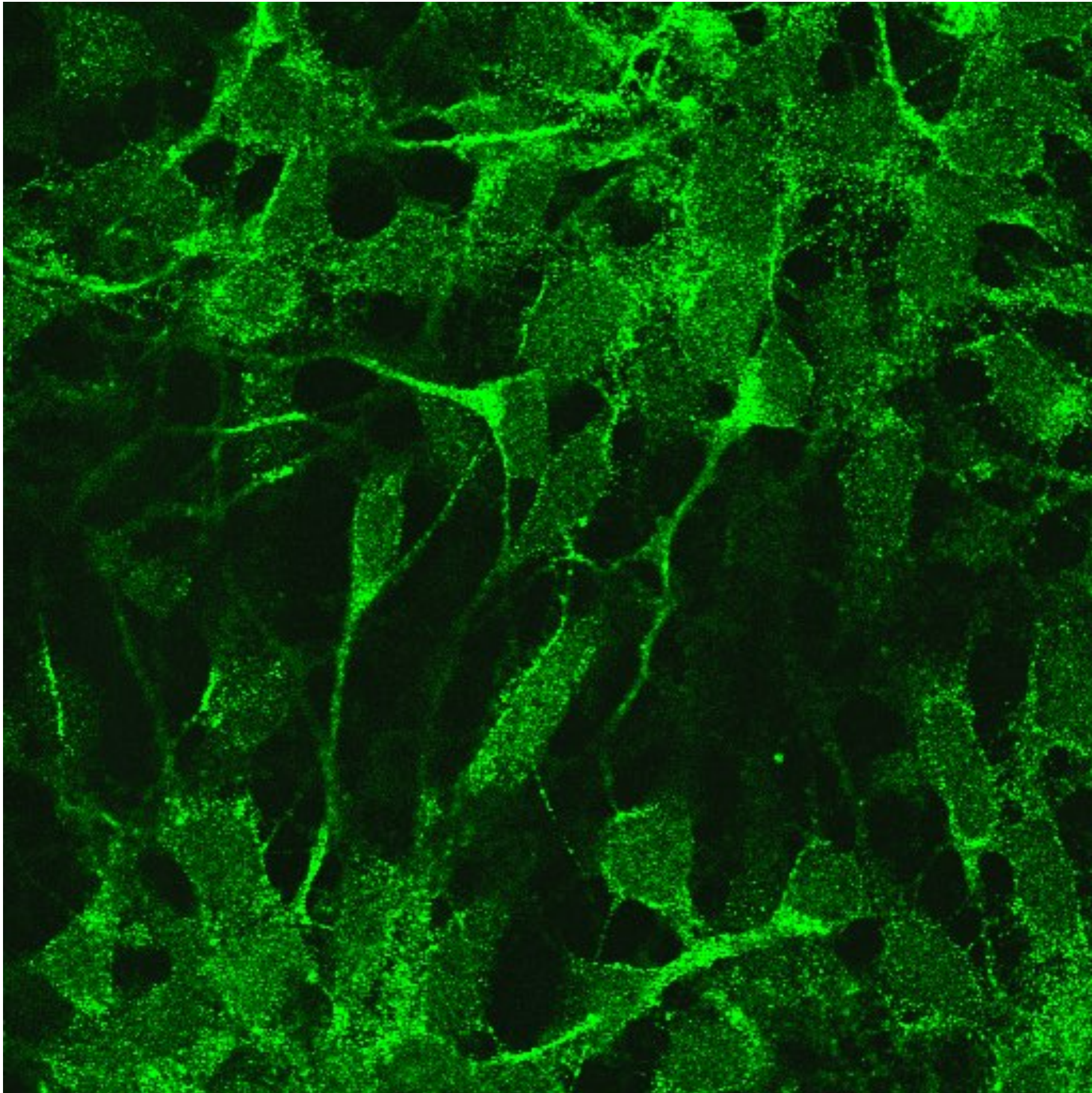

Fig. 6H MAP2

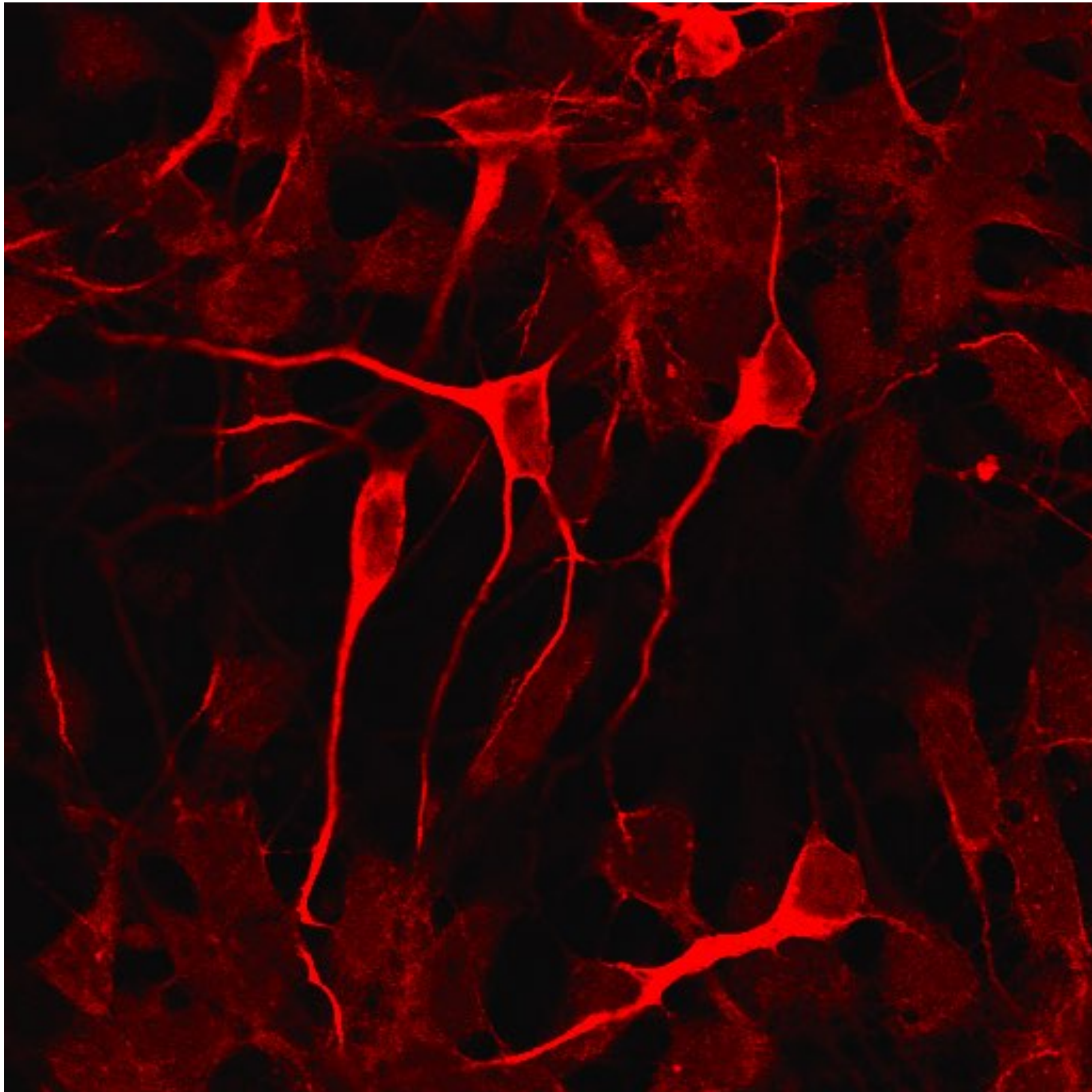

Fig. 6H MERGE

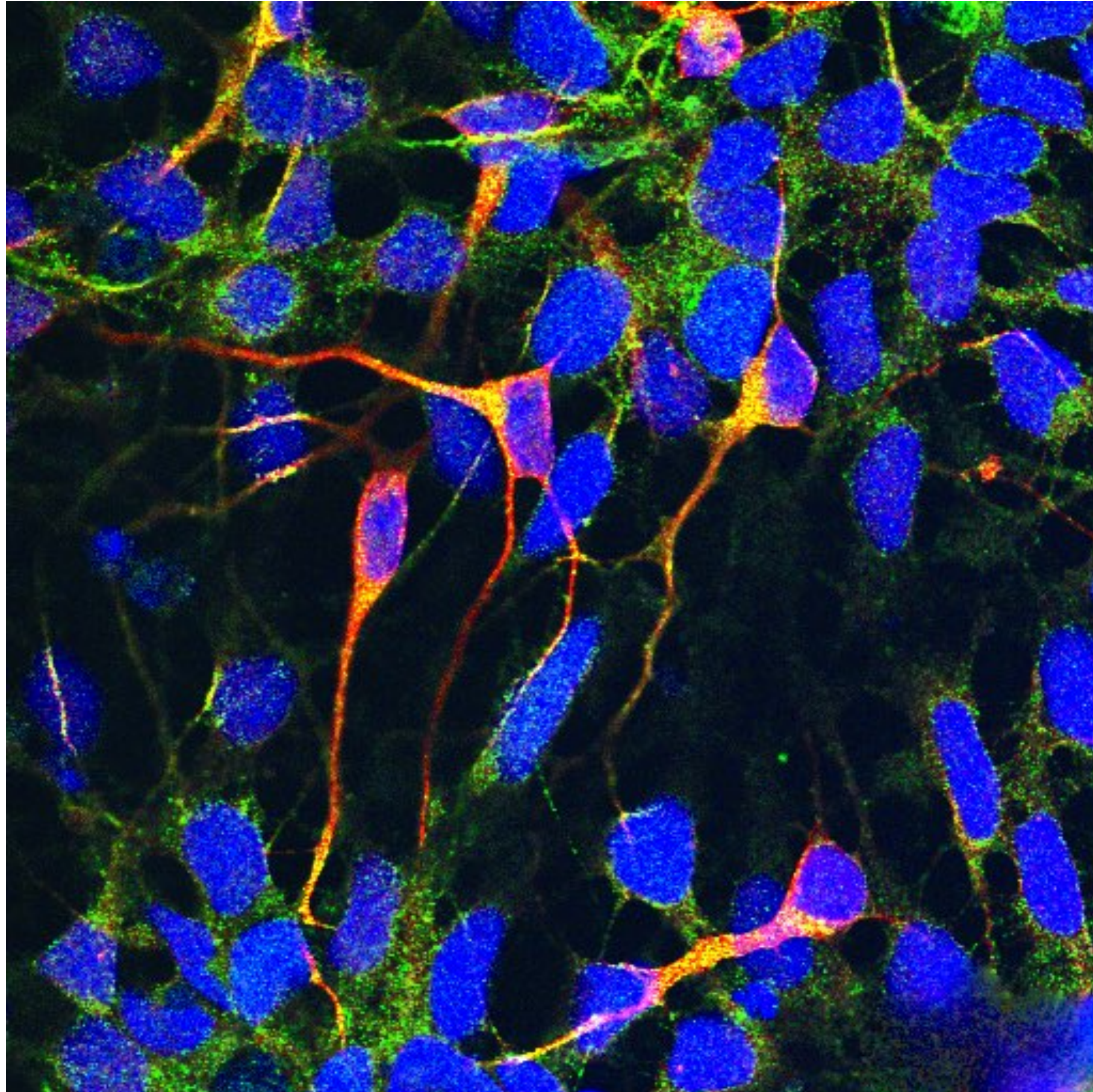

Supplement: Supplementary file 10 — Source Data for Figure 6 [file EMMM-12-e12146-s008.zip › EMM-2020-12146-V5_Source data_Images_Figure 6.pdf]
