# Supplementary material for: Disease‐specific phenotypes in iPSC‐derived neural stem cells with POLG mutations
Source: EMBO Mol Med. 2020 Aug 25;12(10):e12146. doi: 10.15252/emmm.202012146 (PMC7539330; doi:10.15252/emmm.202012146)

Fig. 1A CTRL Fibroblasts

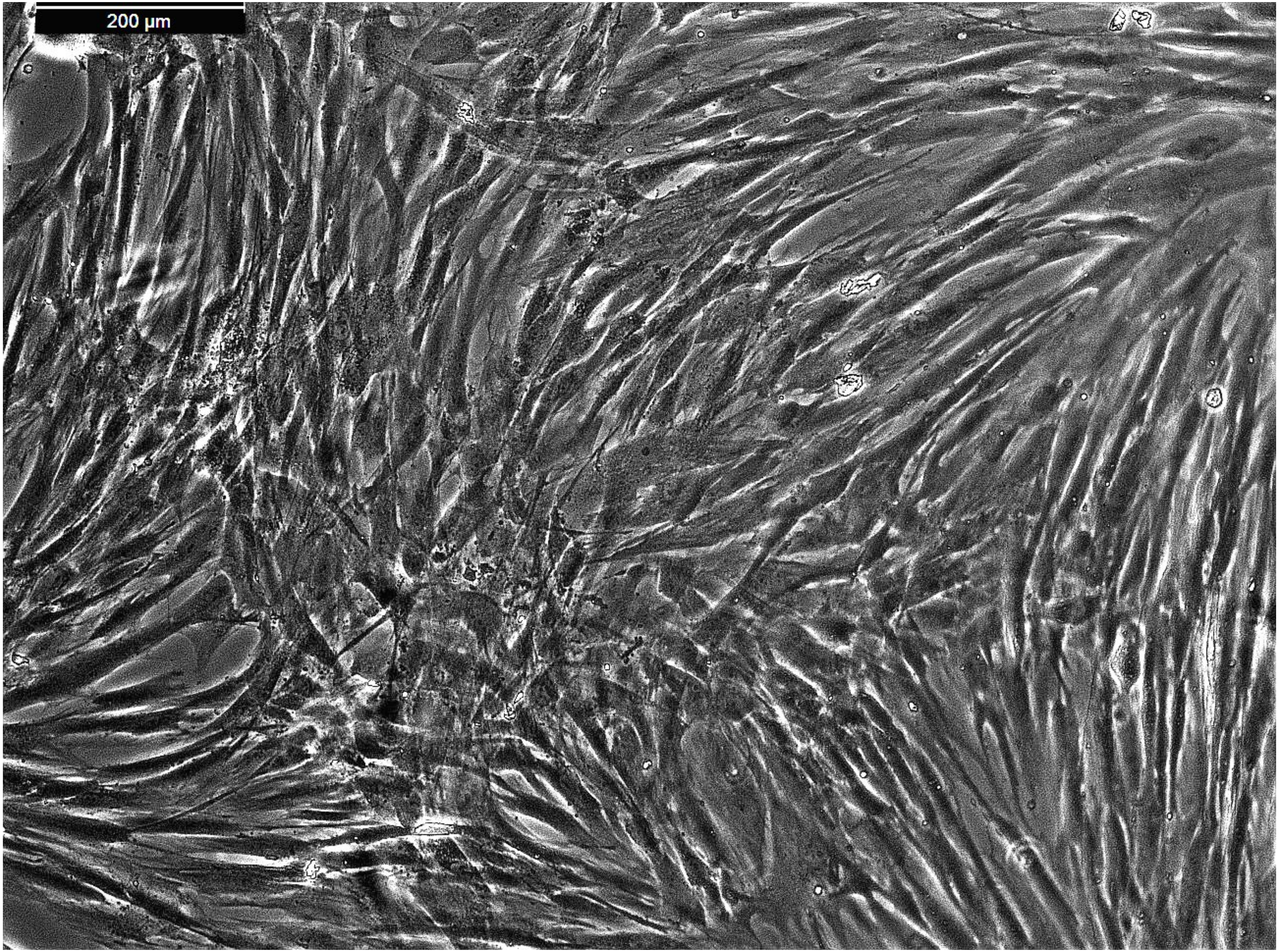

Fig. 1A WS5A Fibroblasts

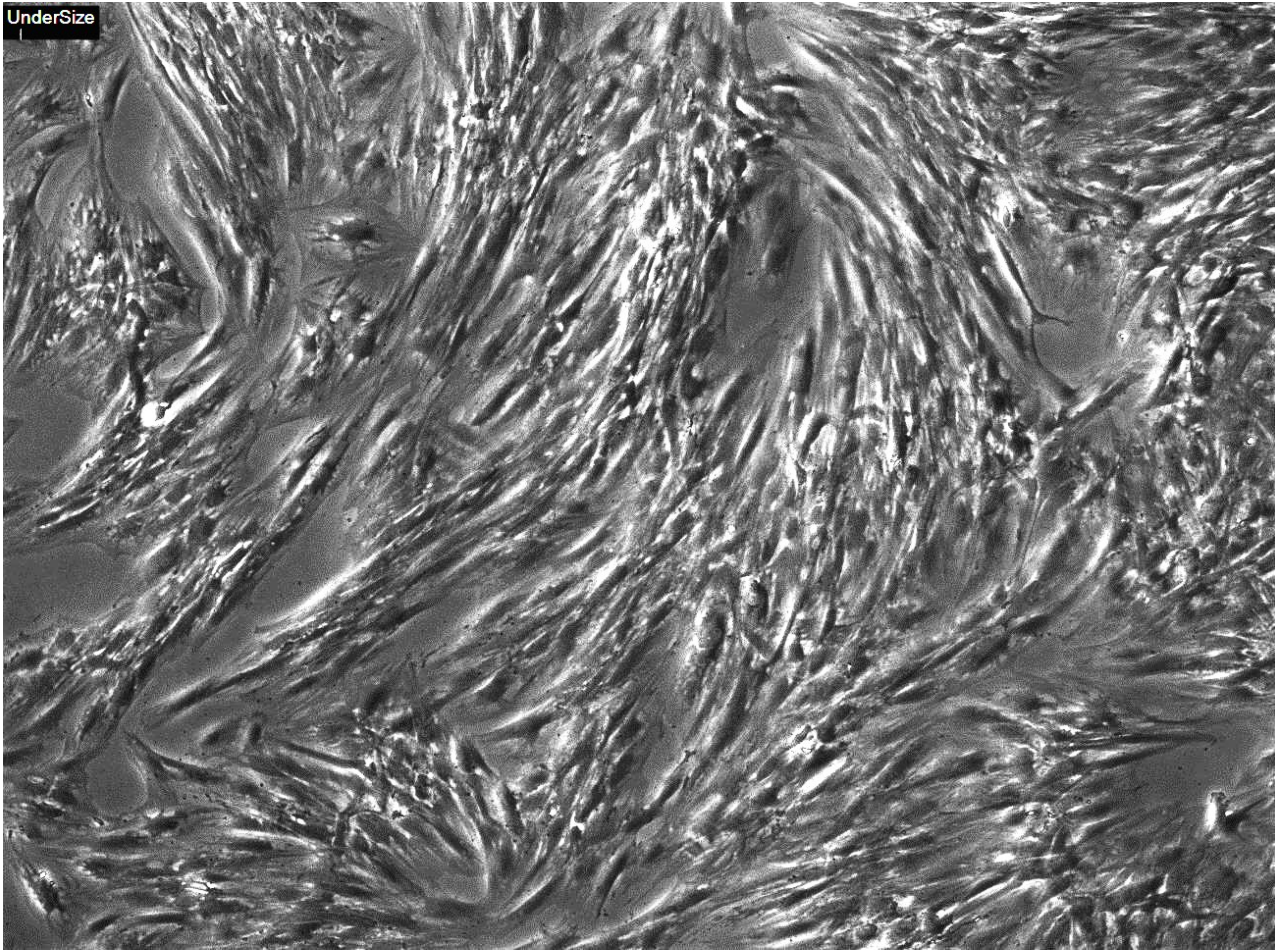

Fig. 1A CP2A Fibroblasts

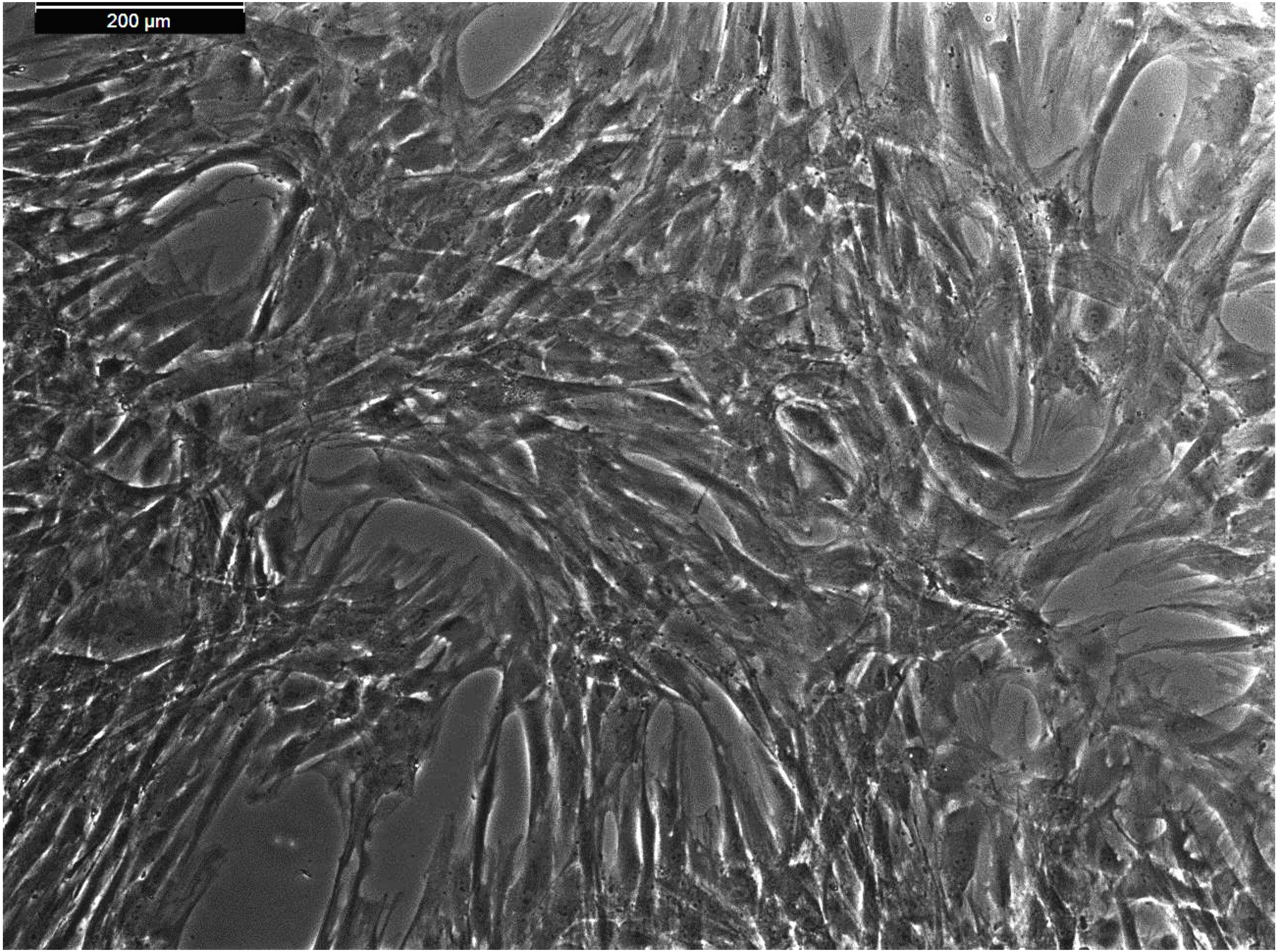

Fig. 1A CTRL iPSCs

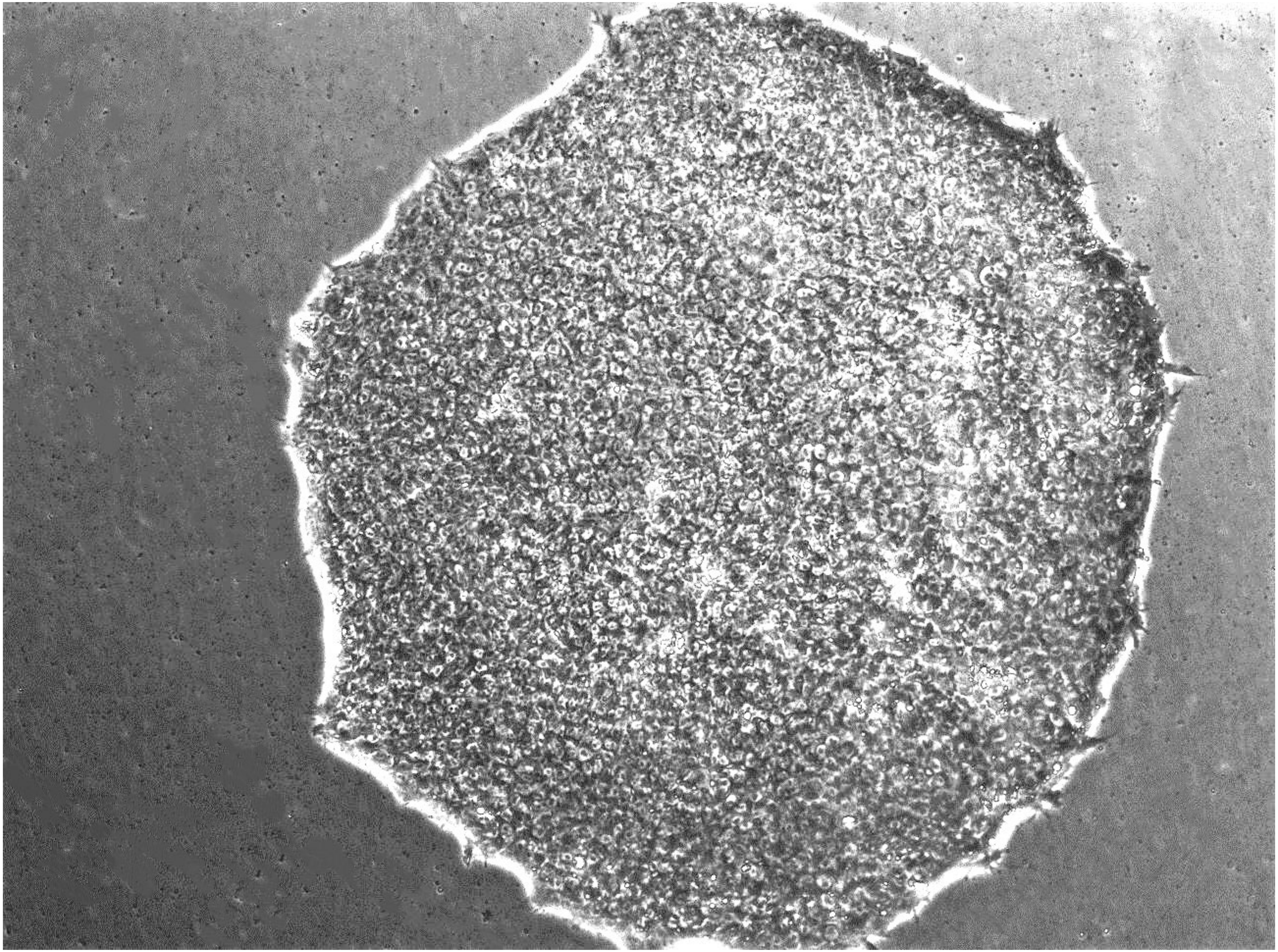

Fig. 1A WS5A iPSCs

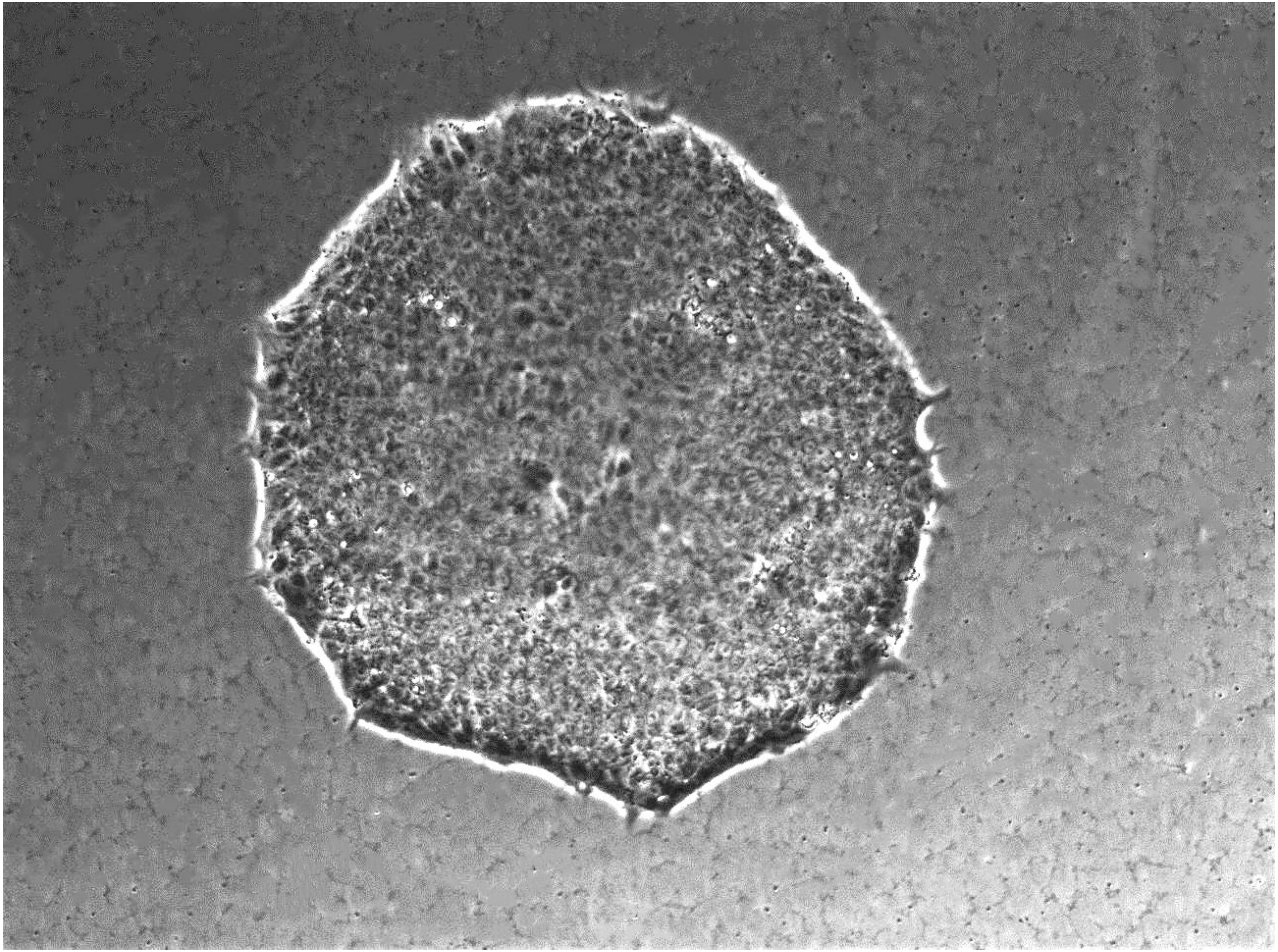

Fig. 1A CP2A iPSCs

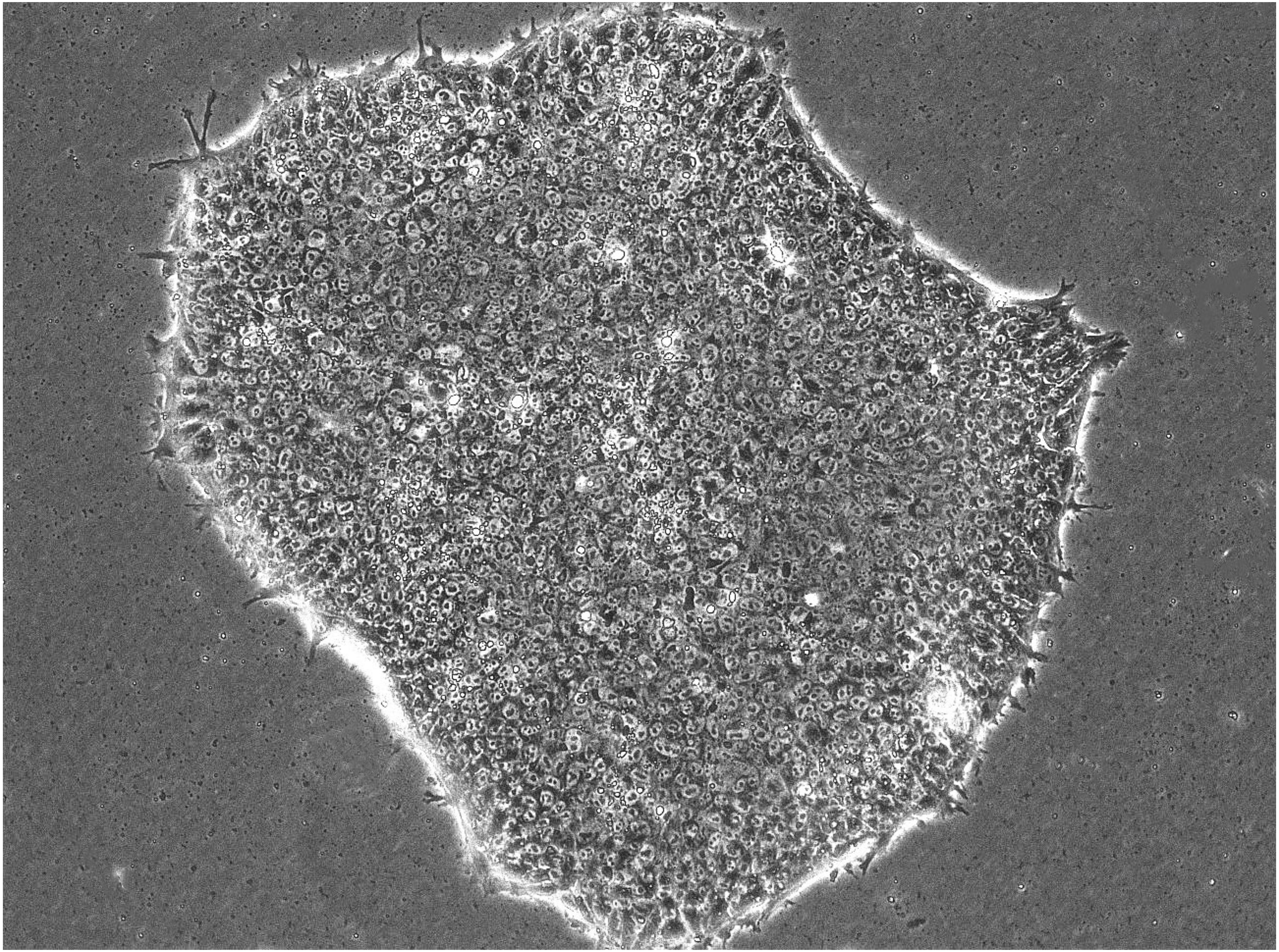

Fig. 1B CTRL iPSC DAPI

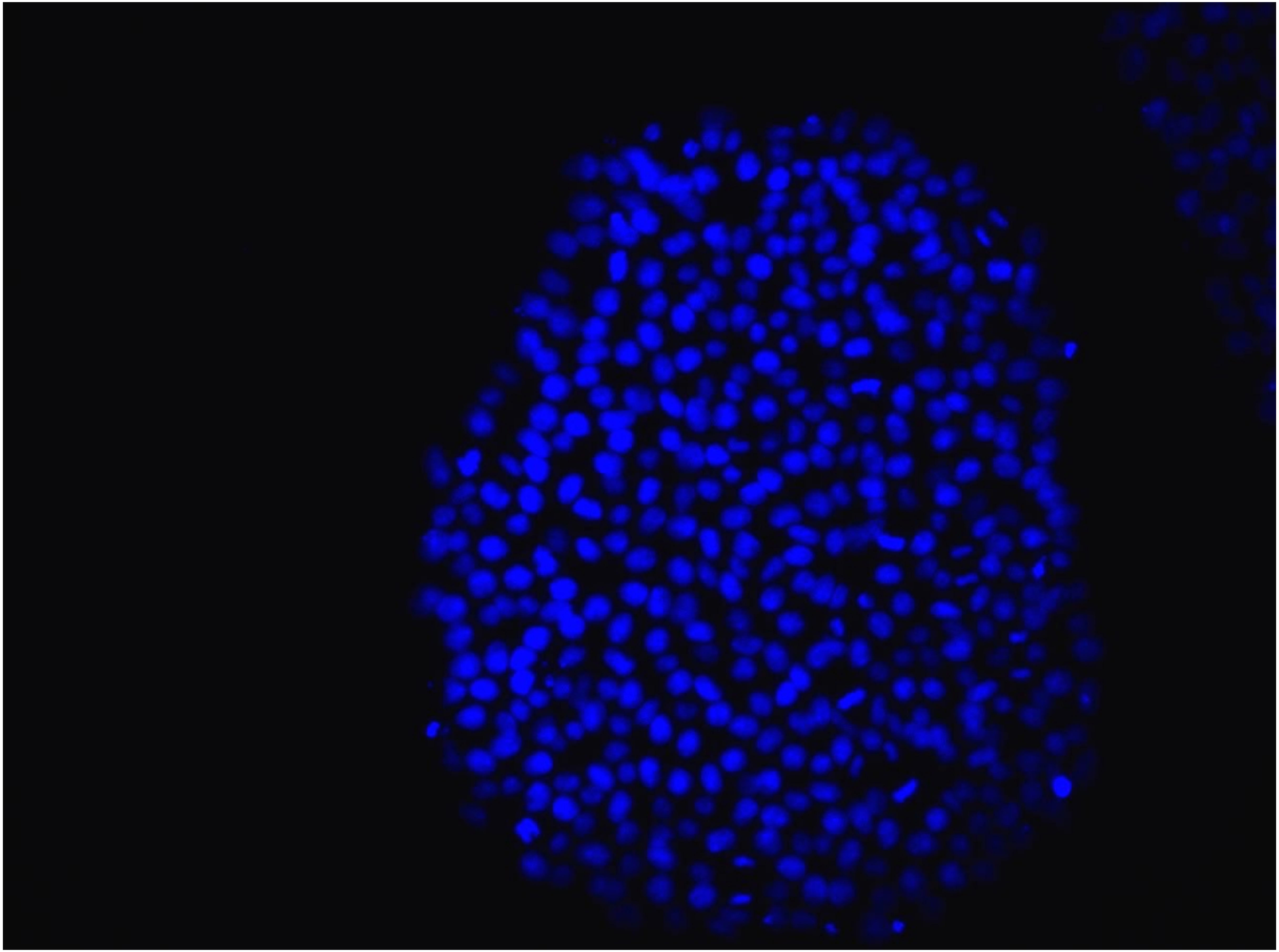

Fig. 1B CTRL iPSC POU5F1

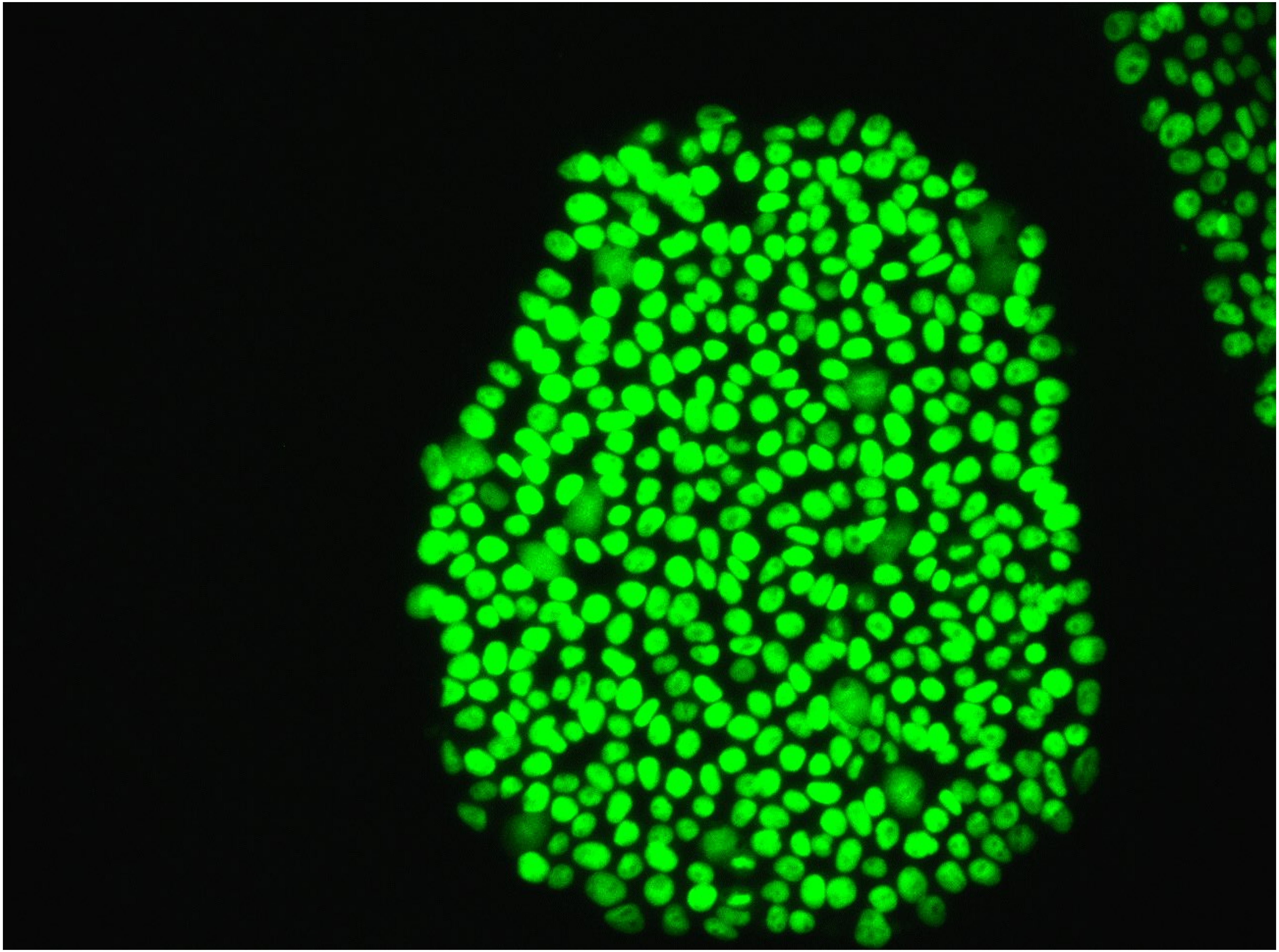

Fig. 1B CTRL iPSC SSEA4

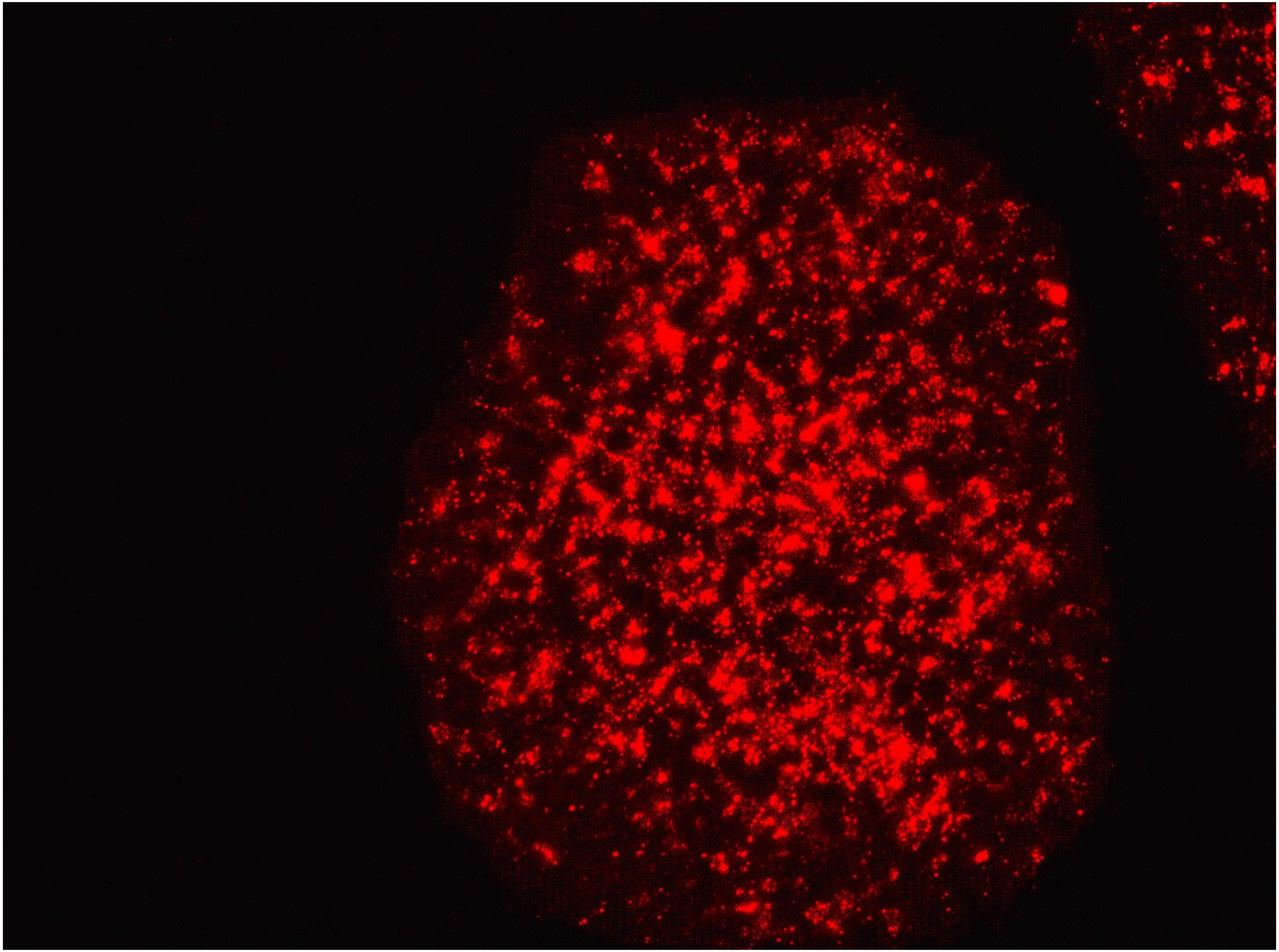

Fig. 1B CTRL iPSC MERGE

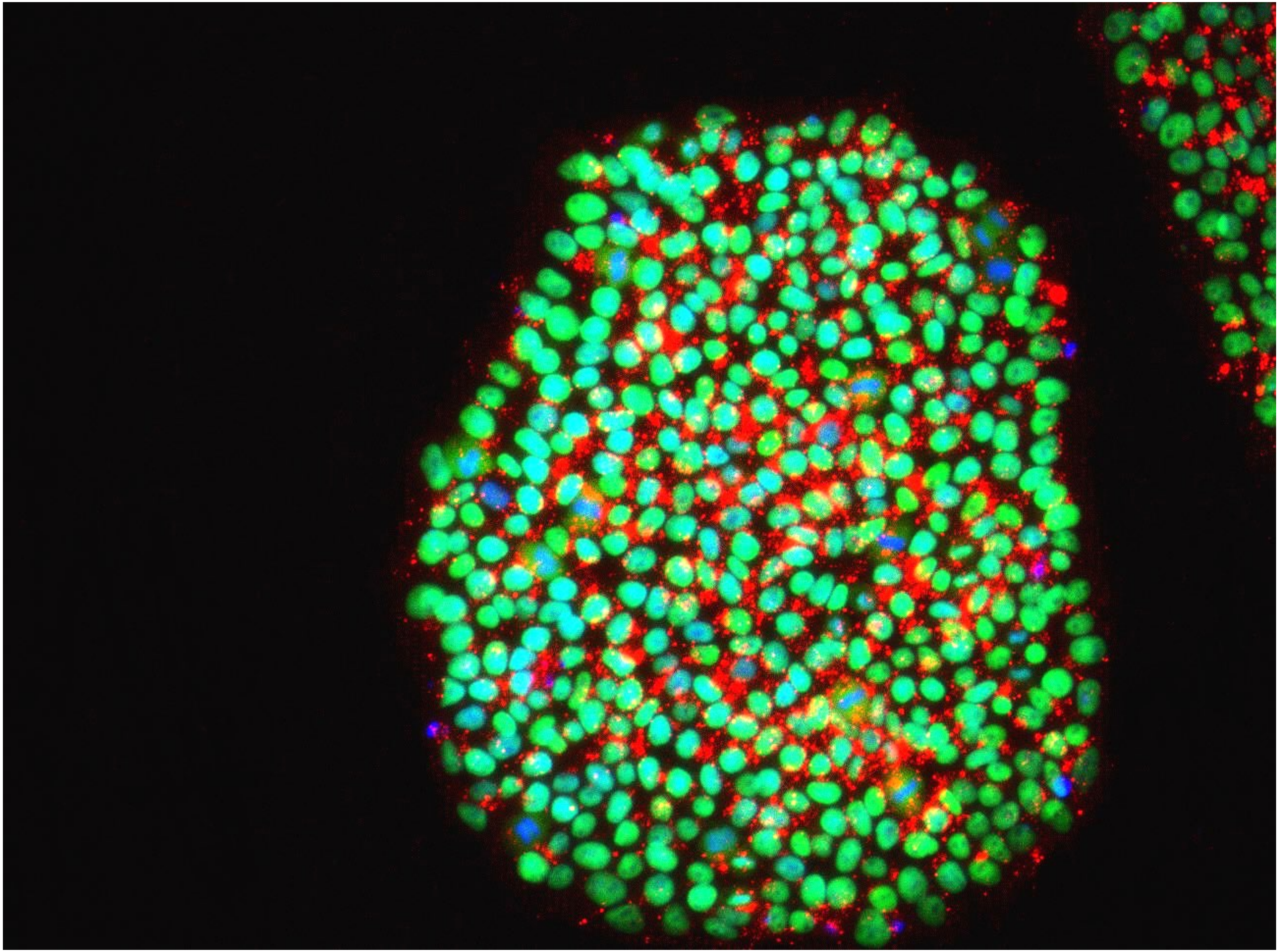

Fig. 1B WS5A iPSC DAPI

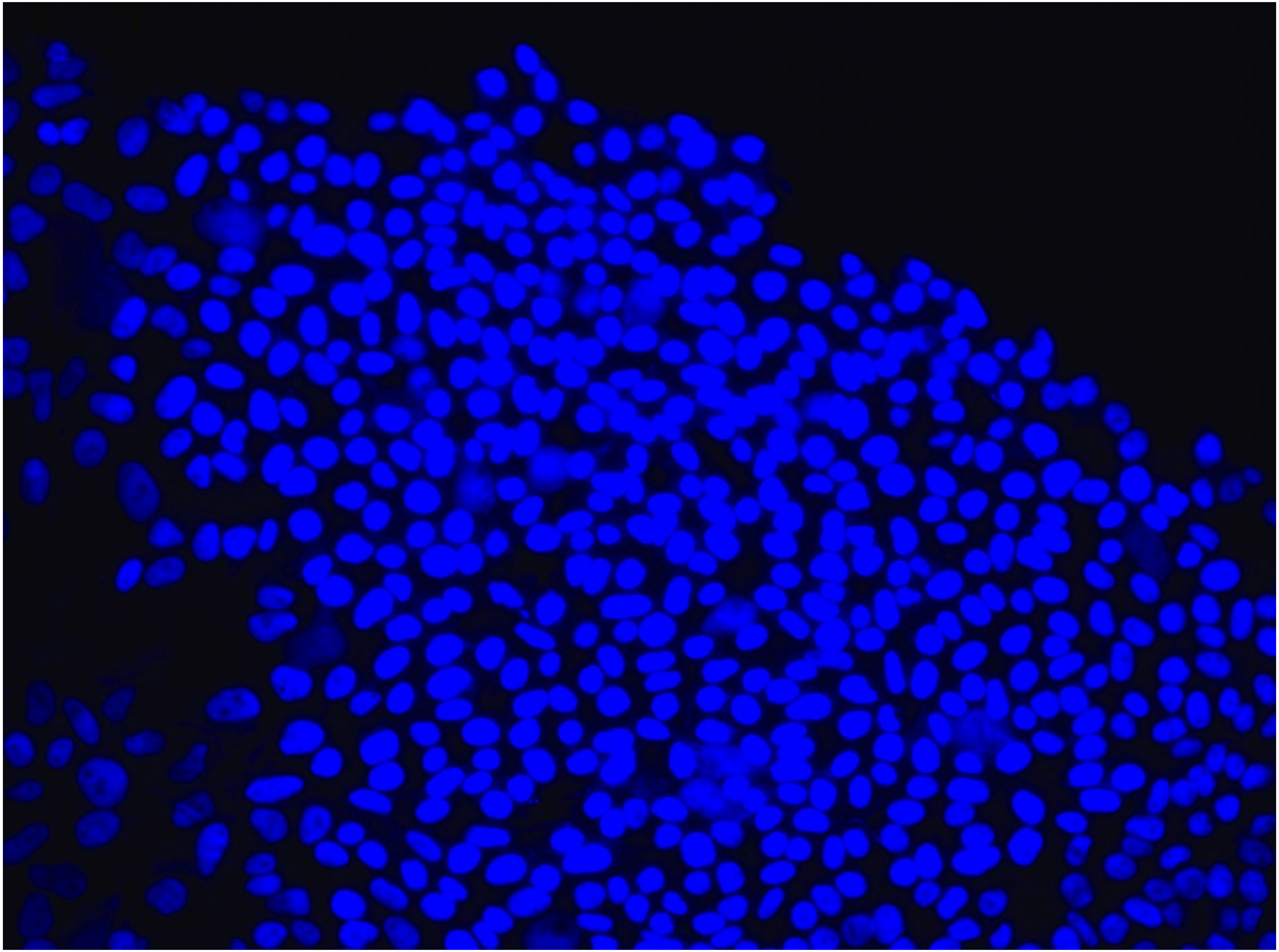

Fig. 1B WS5A iPSC POU5F1

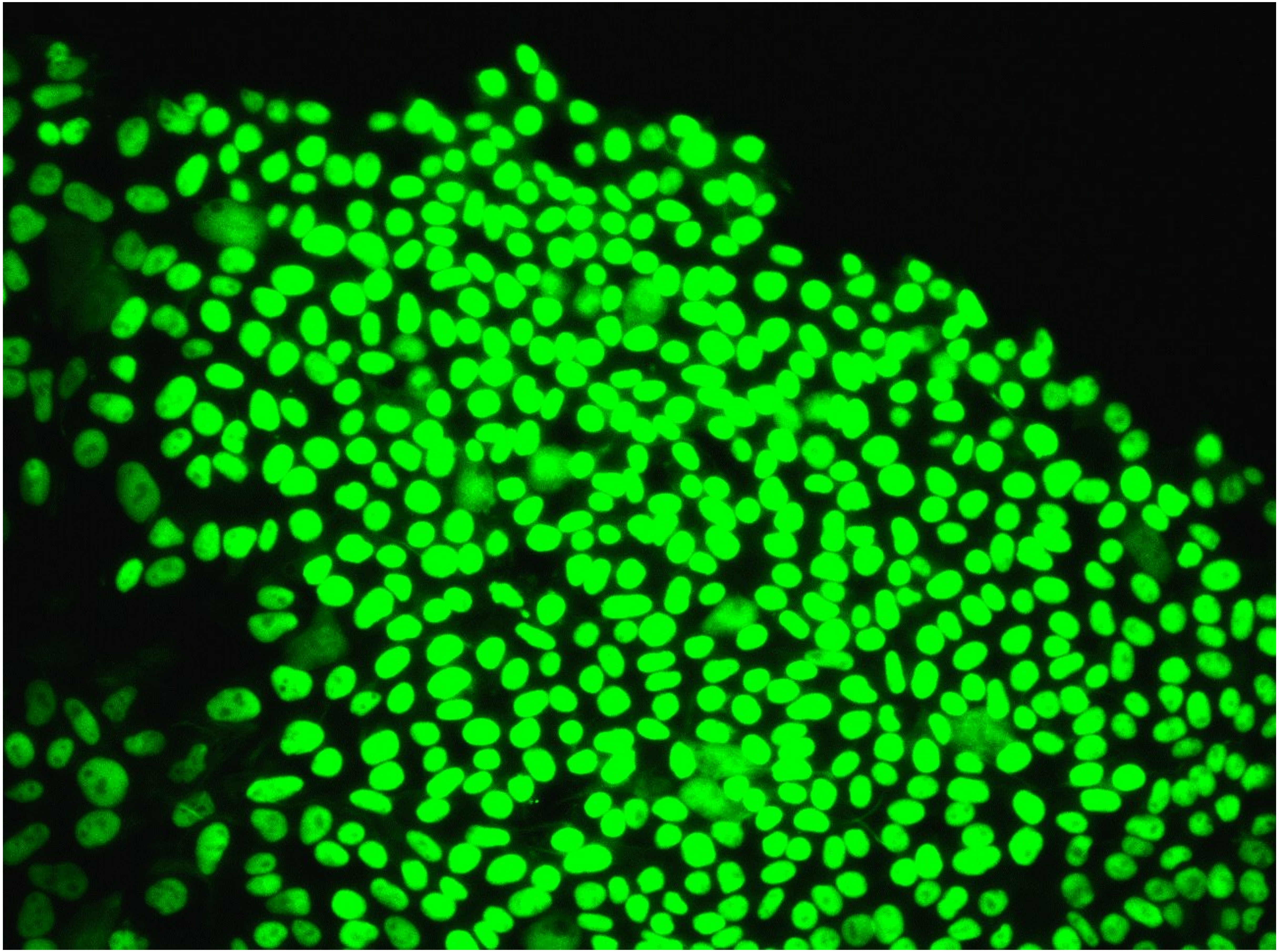

Fig. 1B WS5A iPSC SSEA4

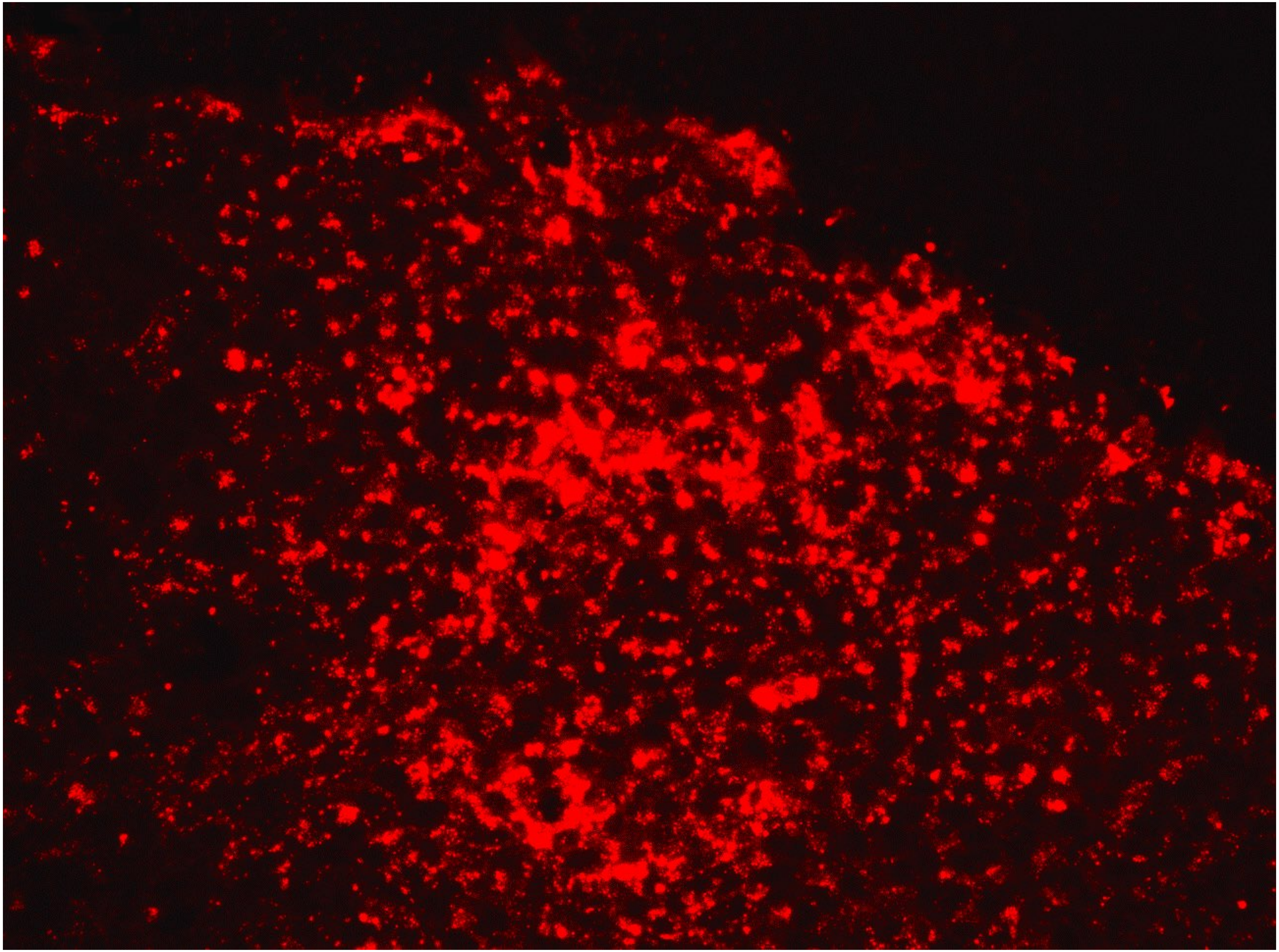

Fig. 1B WS5A iPSC MERGE

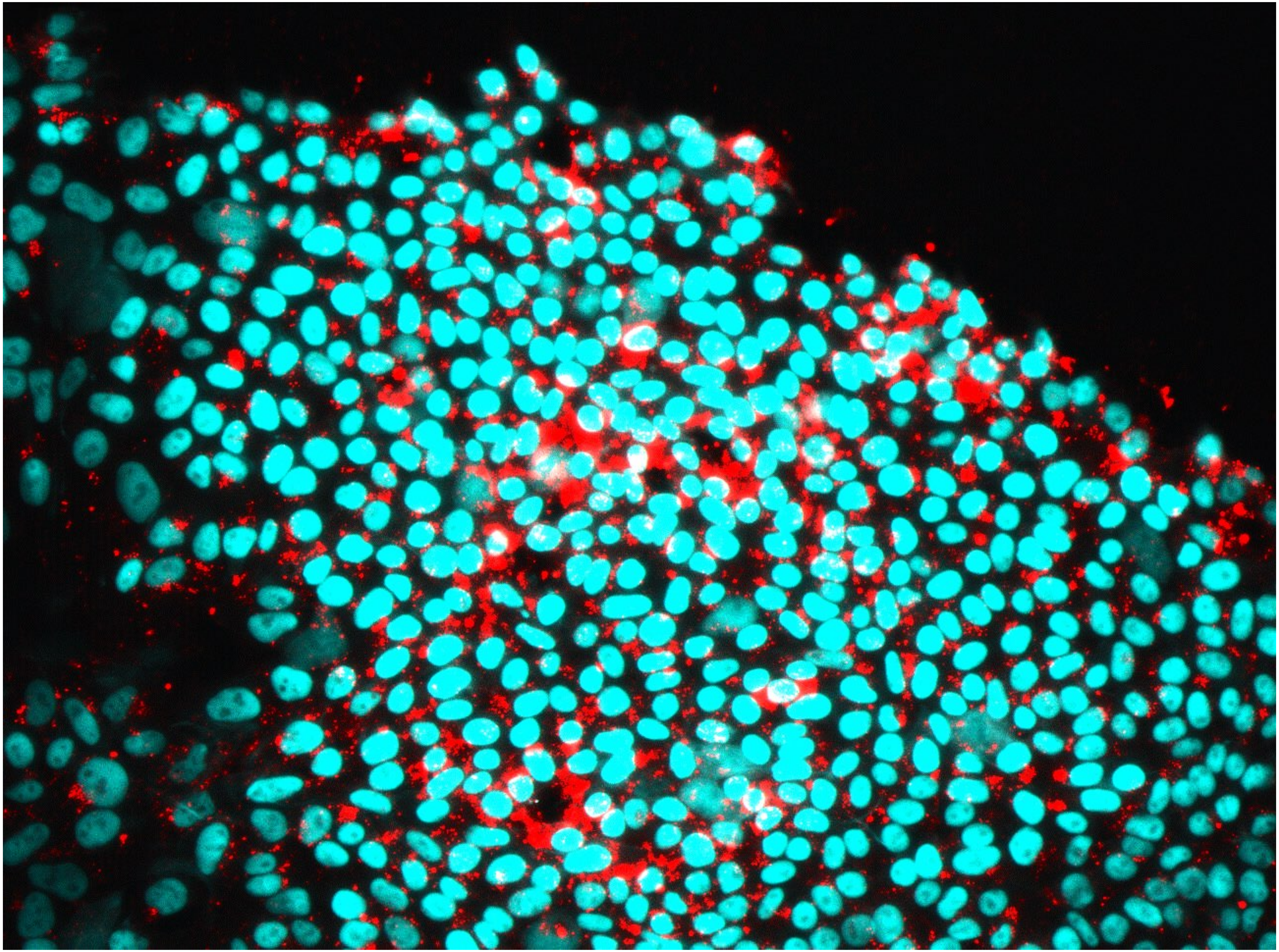

Fig. 1B CP2A iPSC DAPI

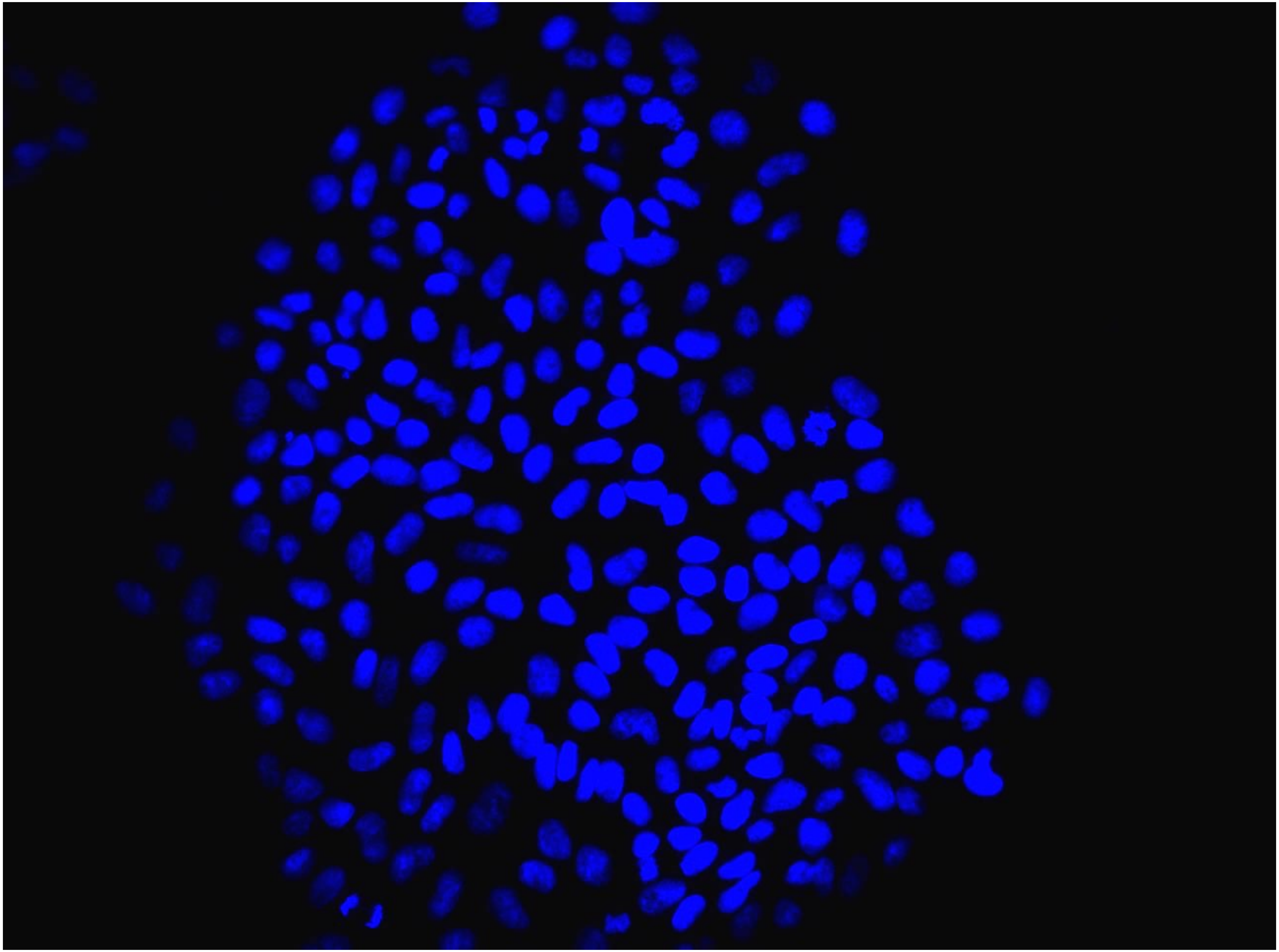

Fig. 1B CP2A iPSC POU5F1

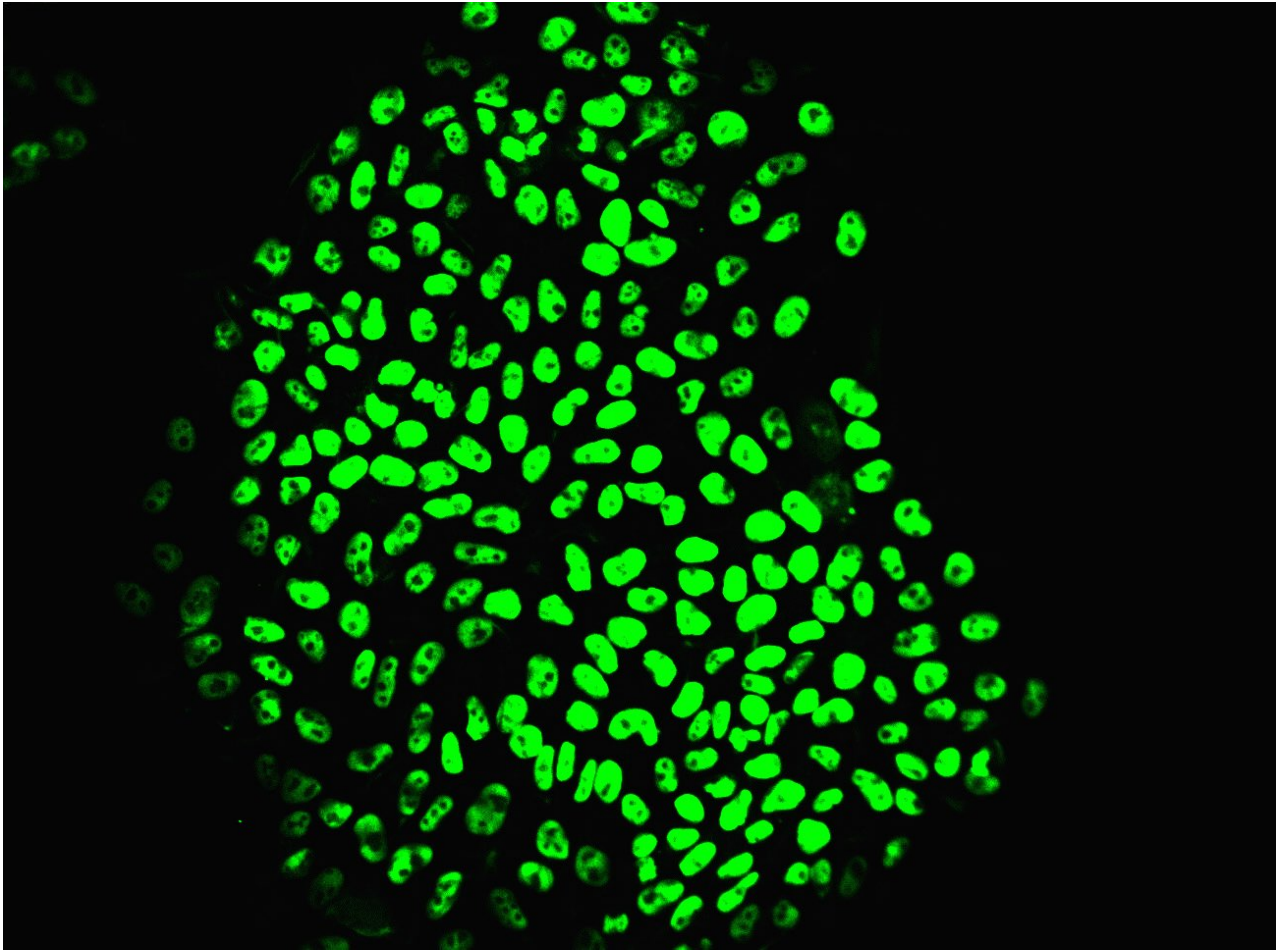

Fig. 1B CP2A iPSC SSEA4

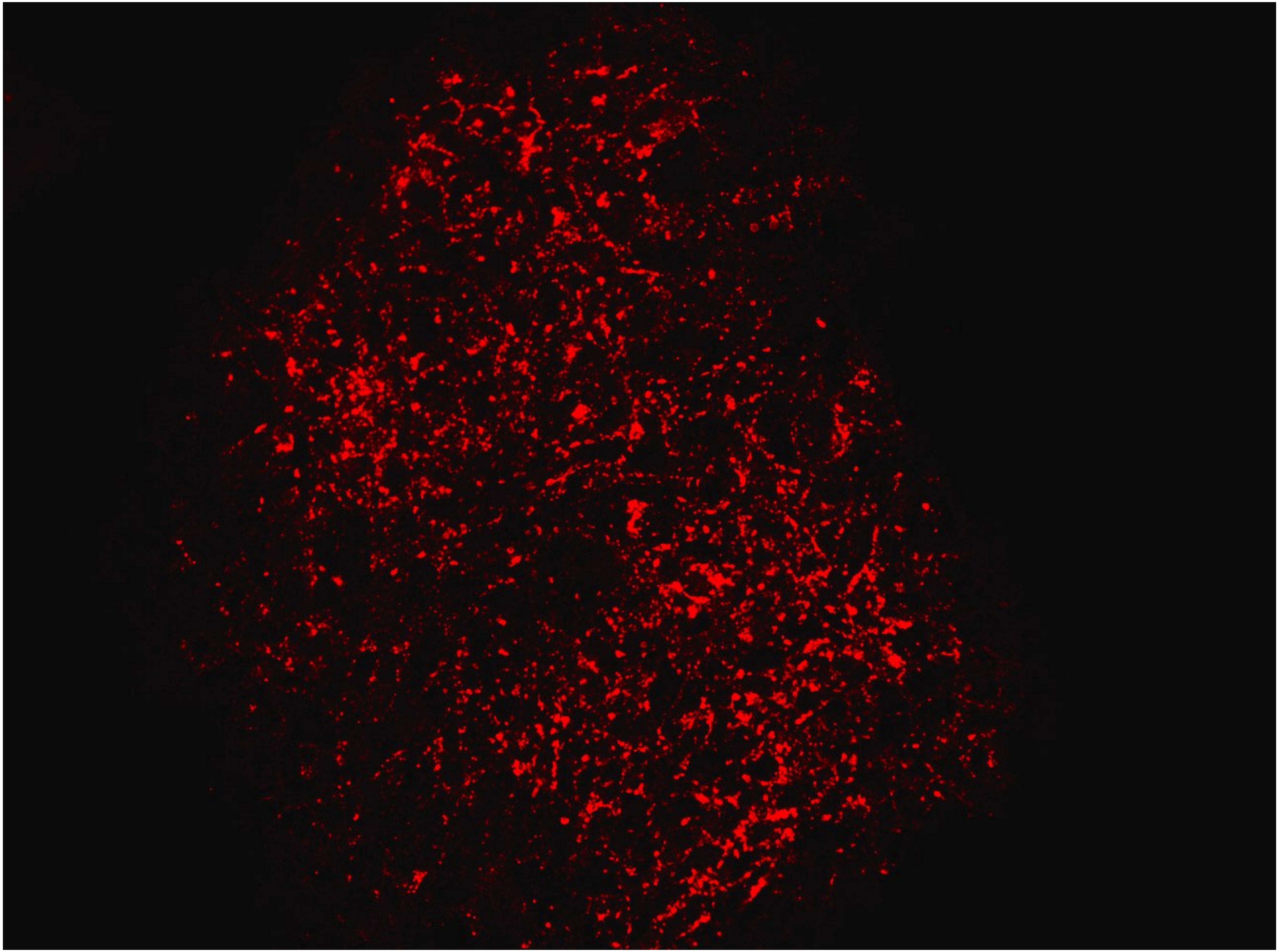

Fig. 1B CP2A iPSC MERGE

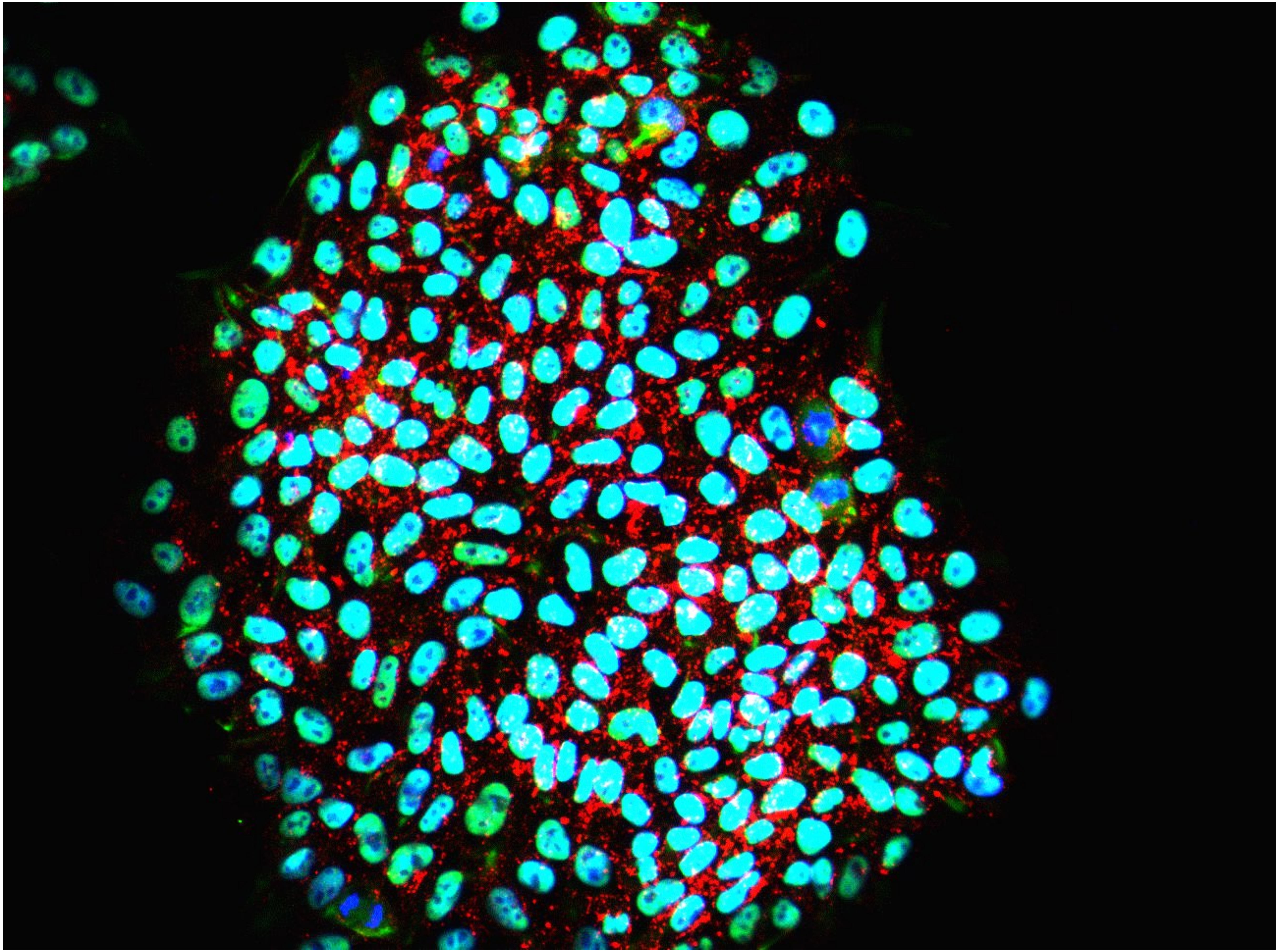

Supplement: Supplementary file 5 — Source Data for Figure 1 [file EMMM-12-e12146-s003.zip › EMM-2020-12146-V5_Source data_Images_Figure 1.pdf]
