# Supplementary material for: Disease‐specific phenotypes in iPSC‐derived neural stem cells with POLG mutations
Source: EMBO Mol Med. 2020 Aug 25;12(10):e12146. doi: 10.15252/emmm.202012146 (PMC7539330; doi:10.15252/emmm.202012146)

Fig. 2A, a Hepatocytes (endoderm): ALBUMIN/ HNF4A/DAPI

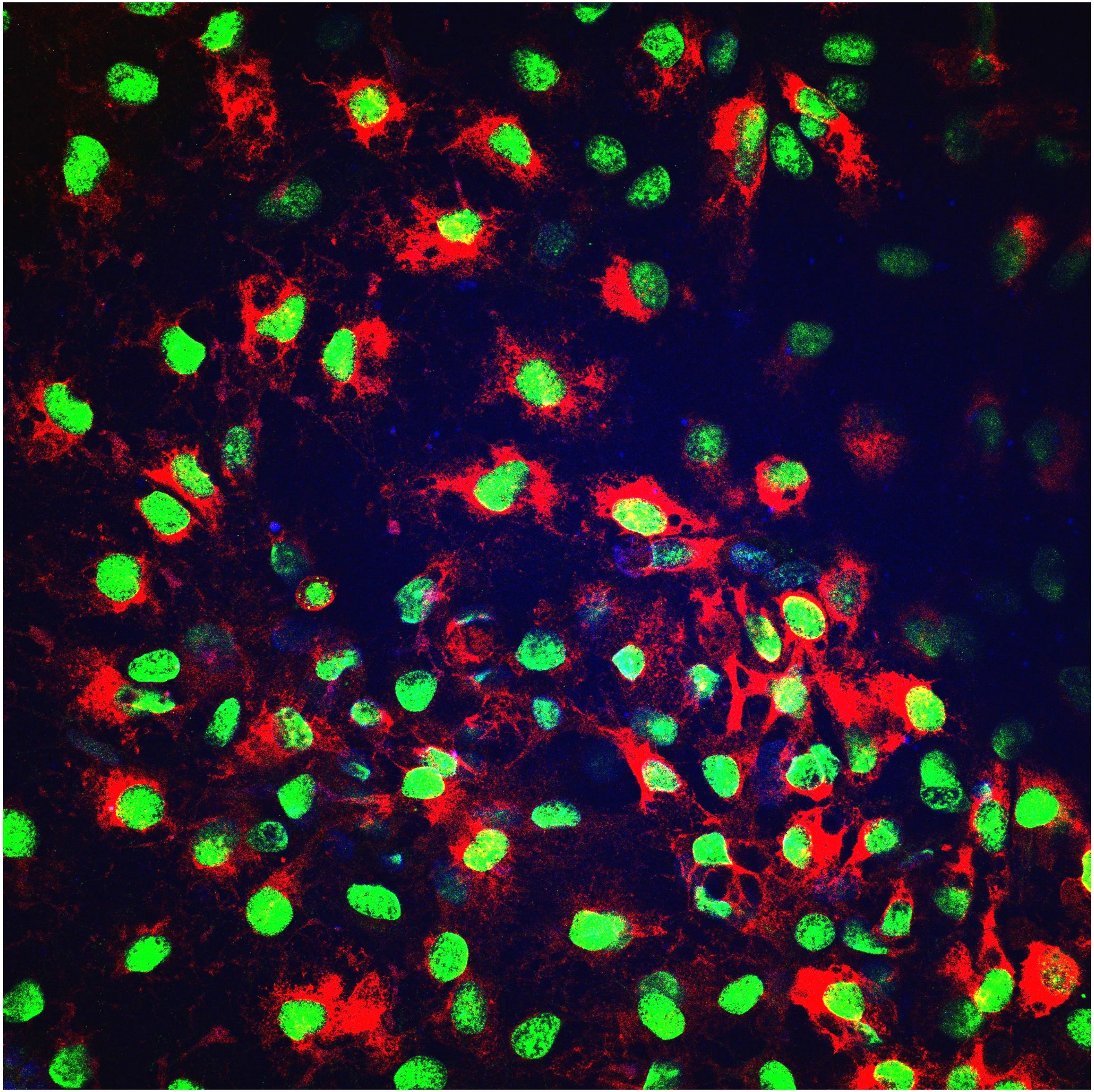

Fig. 2A, b Cardiomyocytes (mesoderm): TNNT2/DAPI

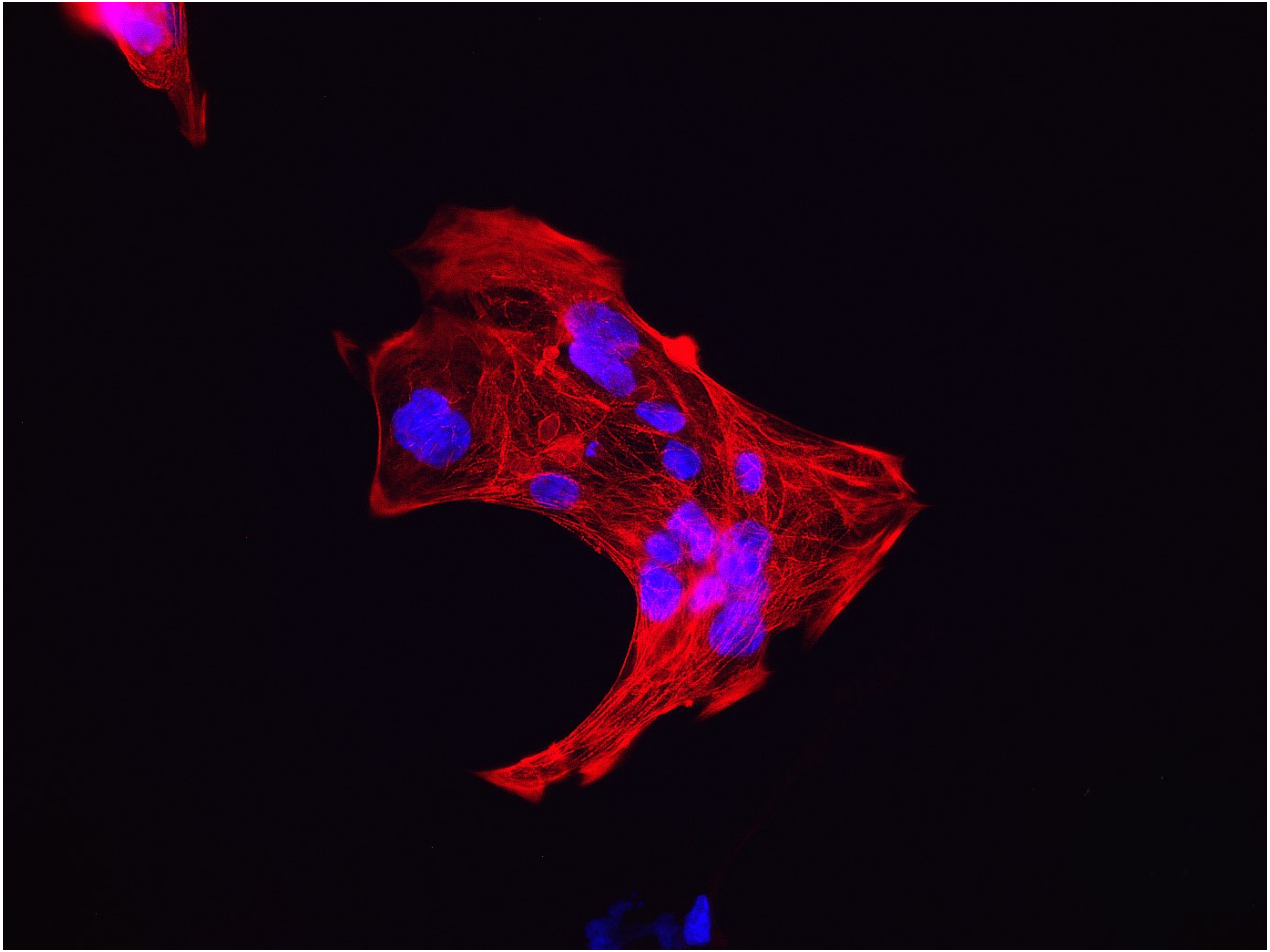

Fig. 2A, c Neurons (ectoderm): TH/MAP2/DAPI

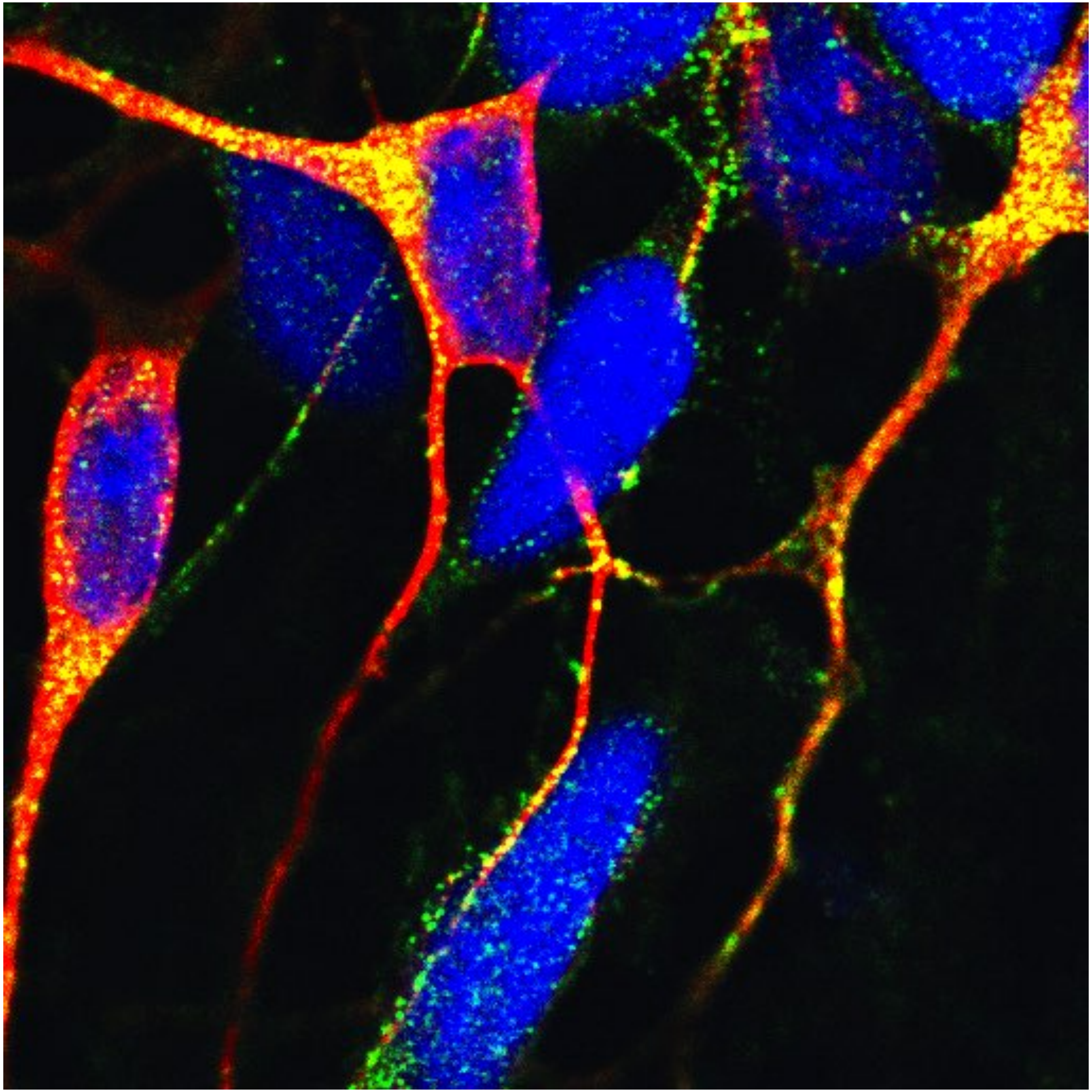

Fig. 2B CTRL iPSC: MTG

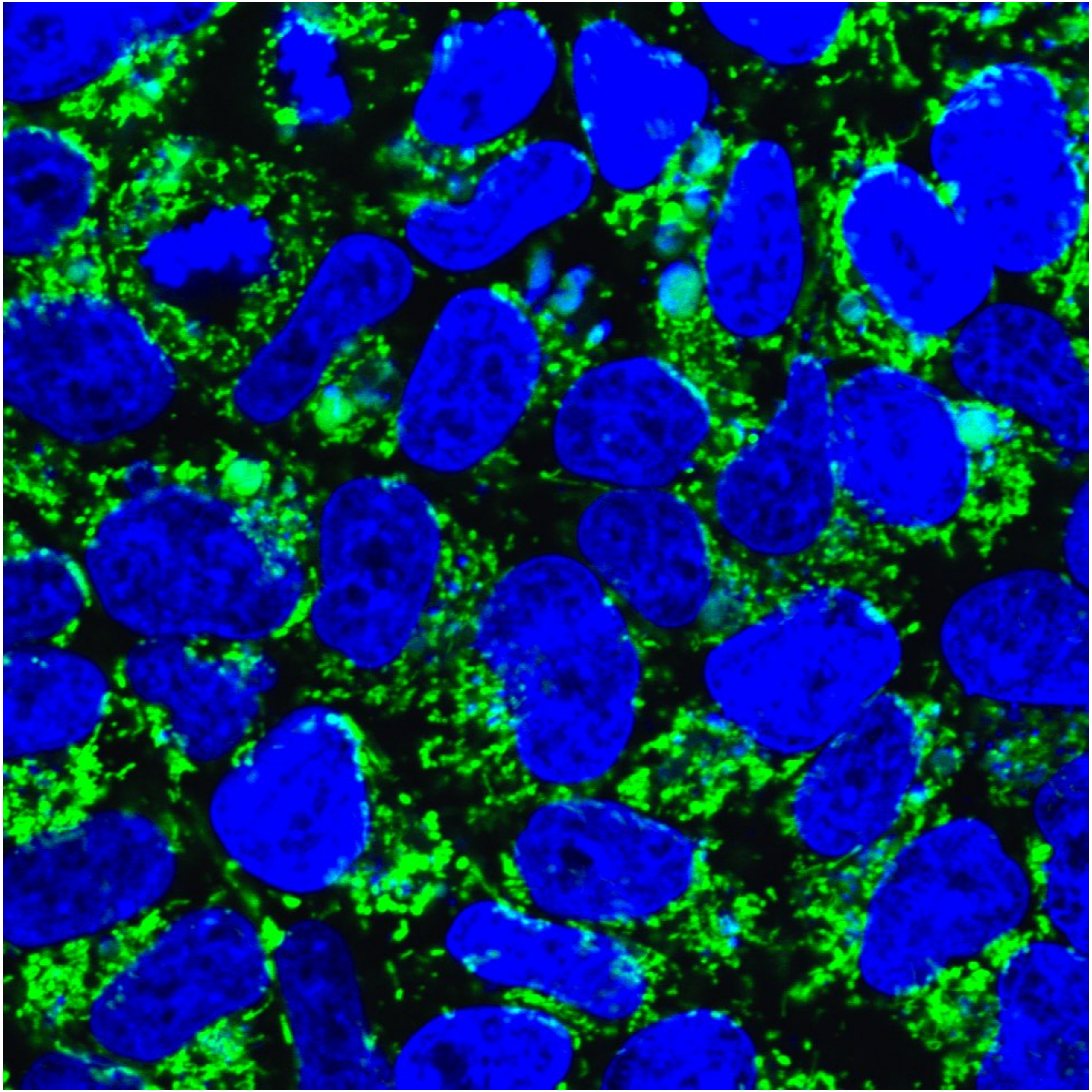

Fig. 2B CTRL iPSC: TMRE

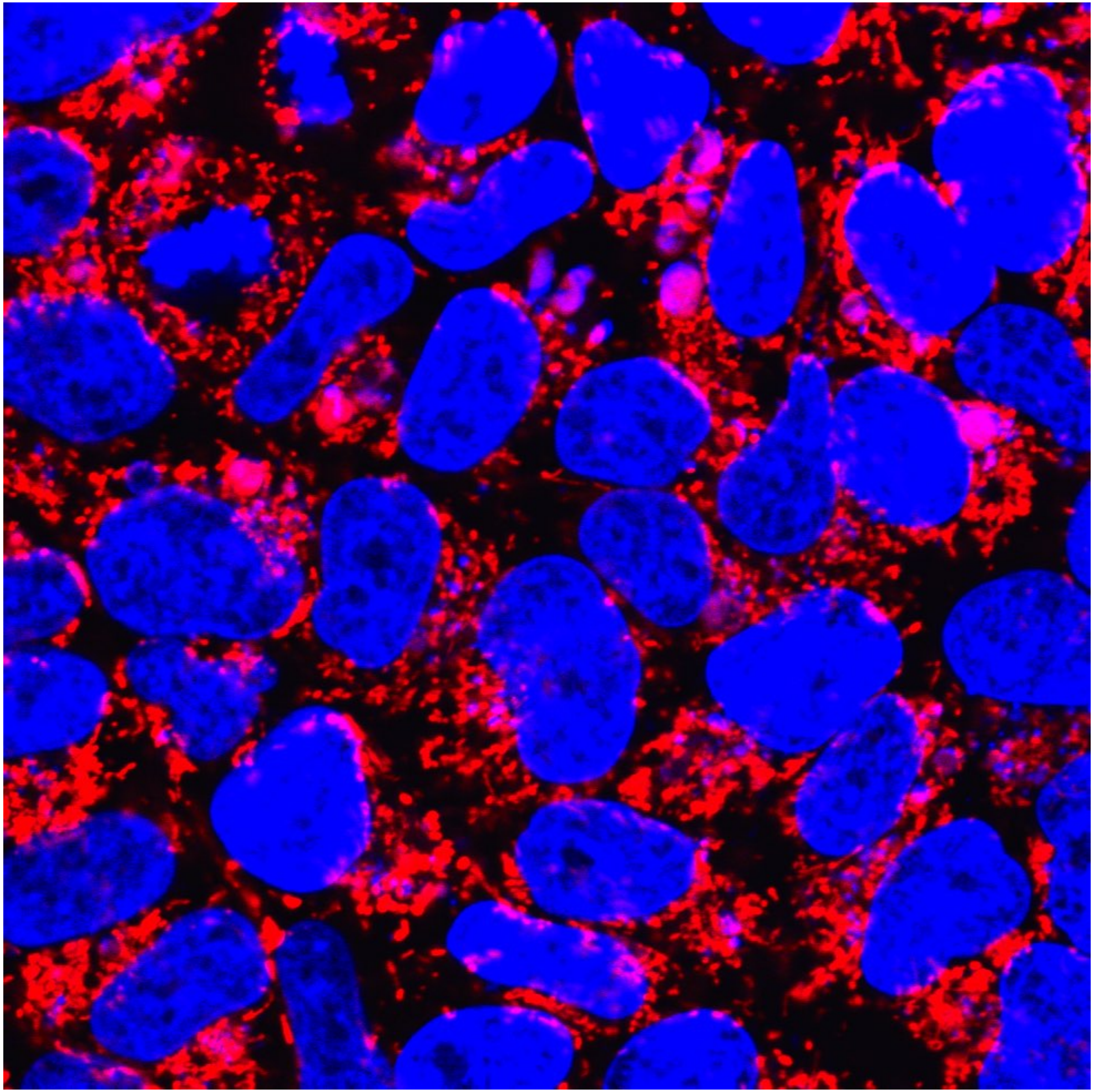

Fig. 2B WS5A iPSC: MTG

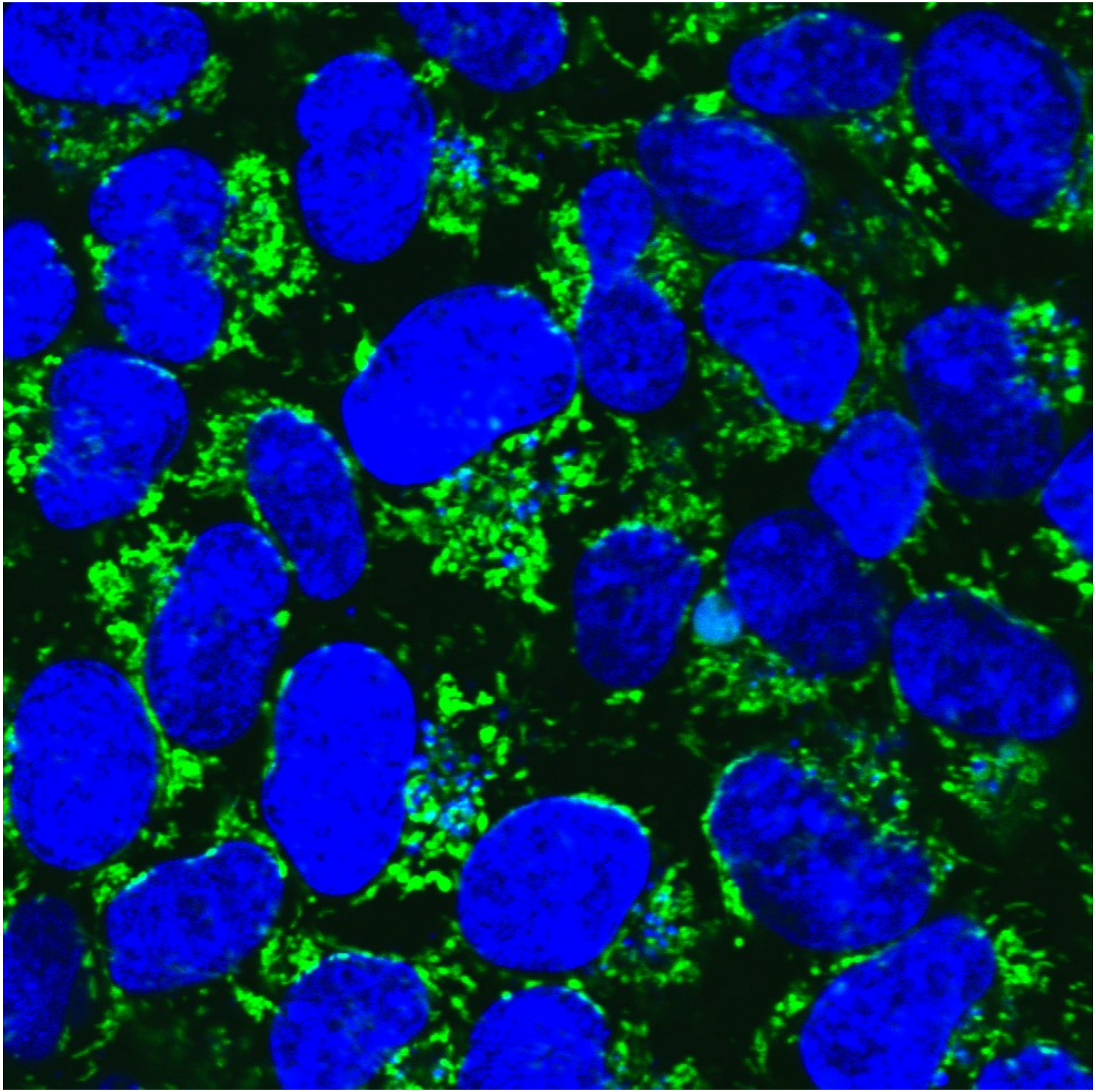

Fig. 2B WS5A iPSC: TMRE

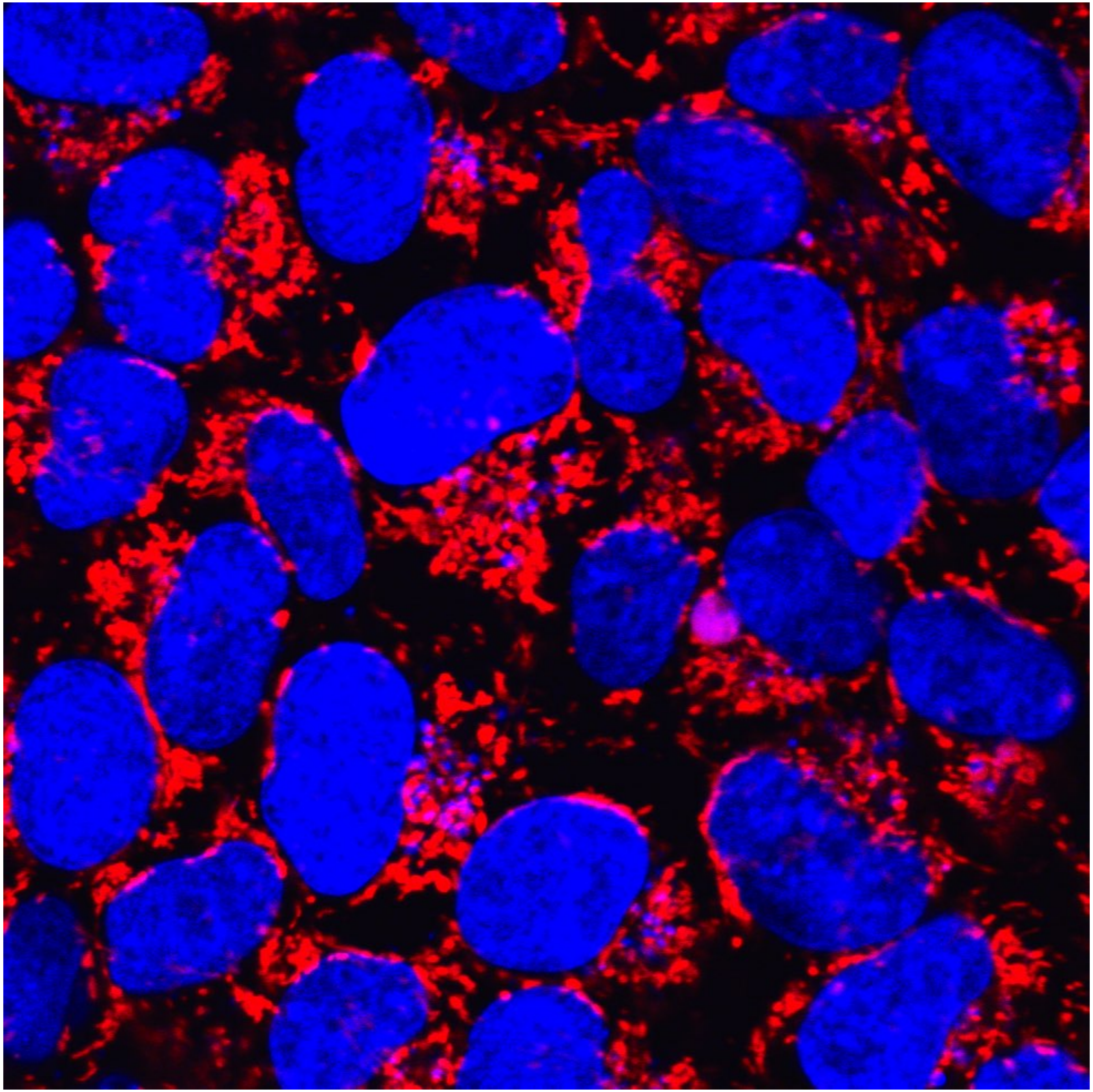

Supplement: Supplementary file 6 — Source Data for Figure 2 [file EMMM-12-e12146-s004.zip › EMM-2020-12146-V5_Source data_Images_Figure 2.pdf]
