# Supplementary material for: Disease‐specific phenotypes in iPSC‐derived neural stem cells with POLG mutations
Source: EMBO Mol Med. 2020 Aug 25;12(10):e12146. doi: 10.15252/emmm.202012146 (PMC7539330; doi:10.15252/emmm.202012146)

Fig. 3B, a D0 - iPSC

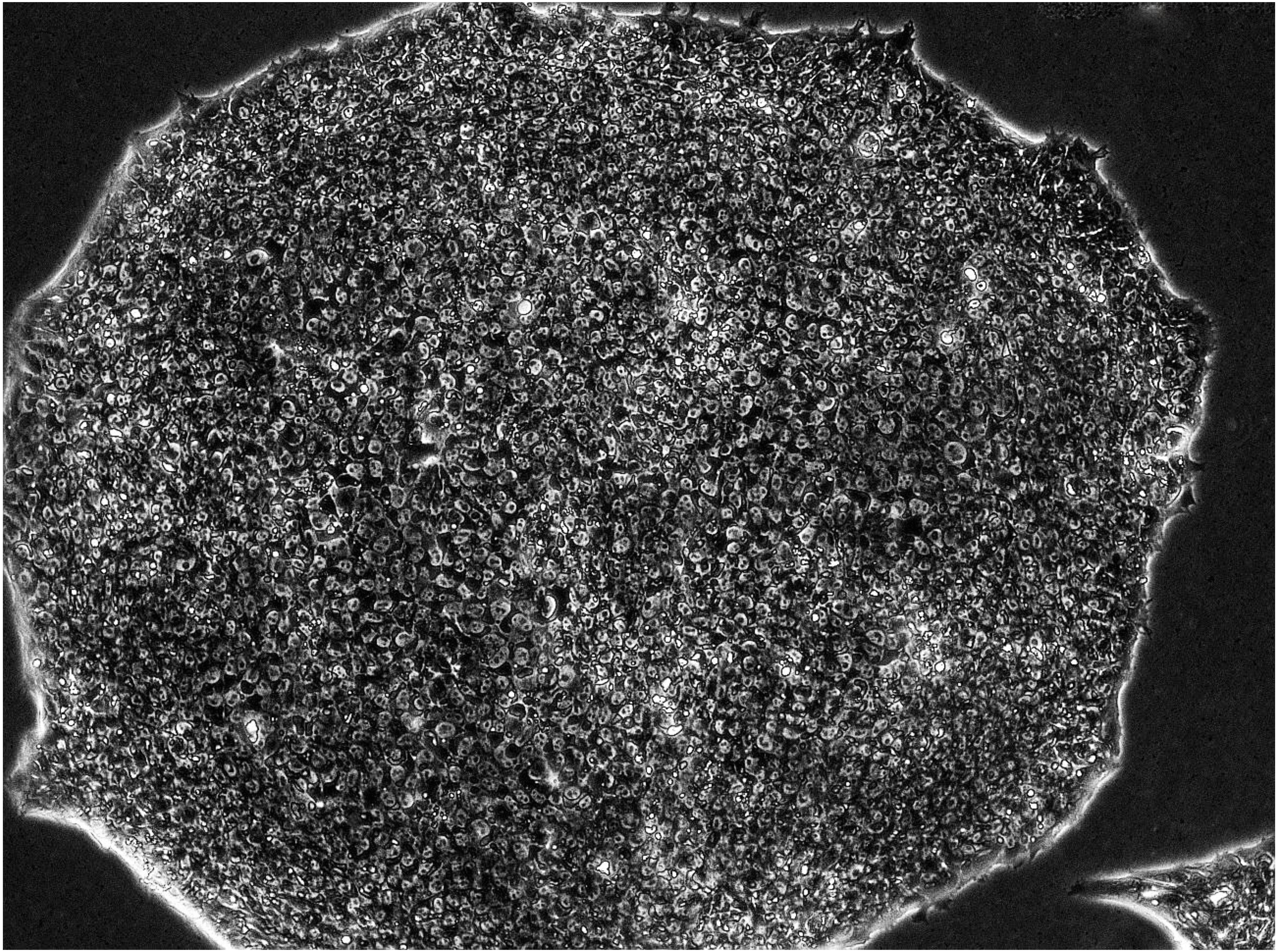

Fig. 3B, b D5 - Neuroepithelium

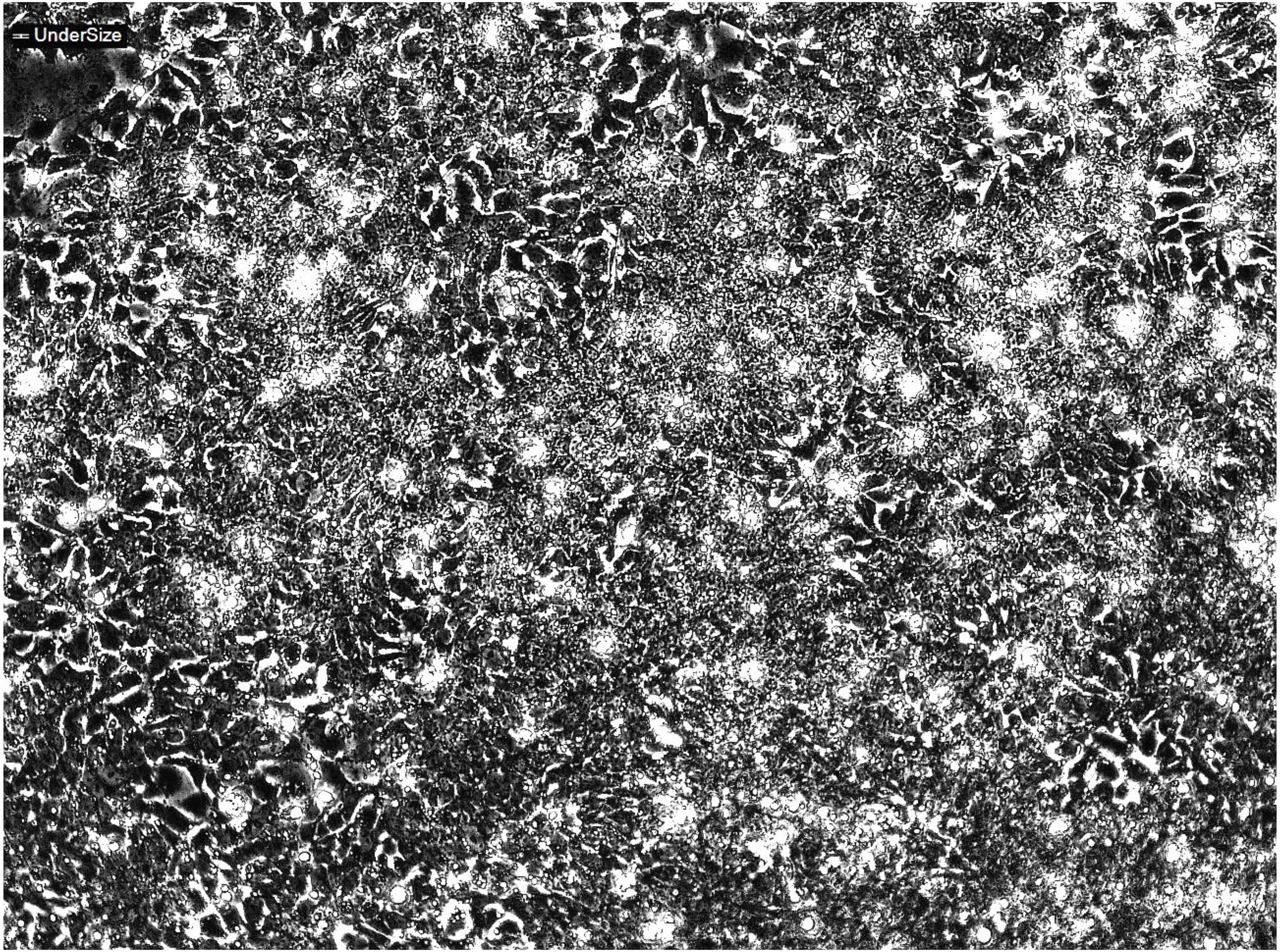

Fig. 3B, c D6 - D9 - Neurospheres

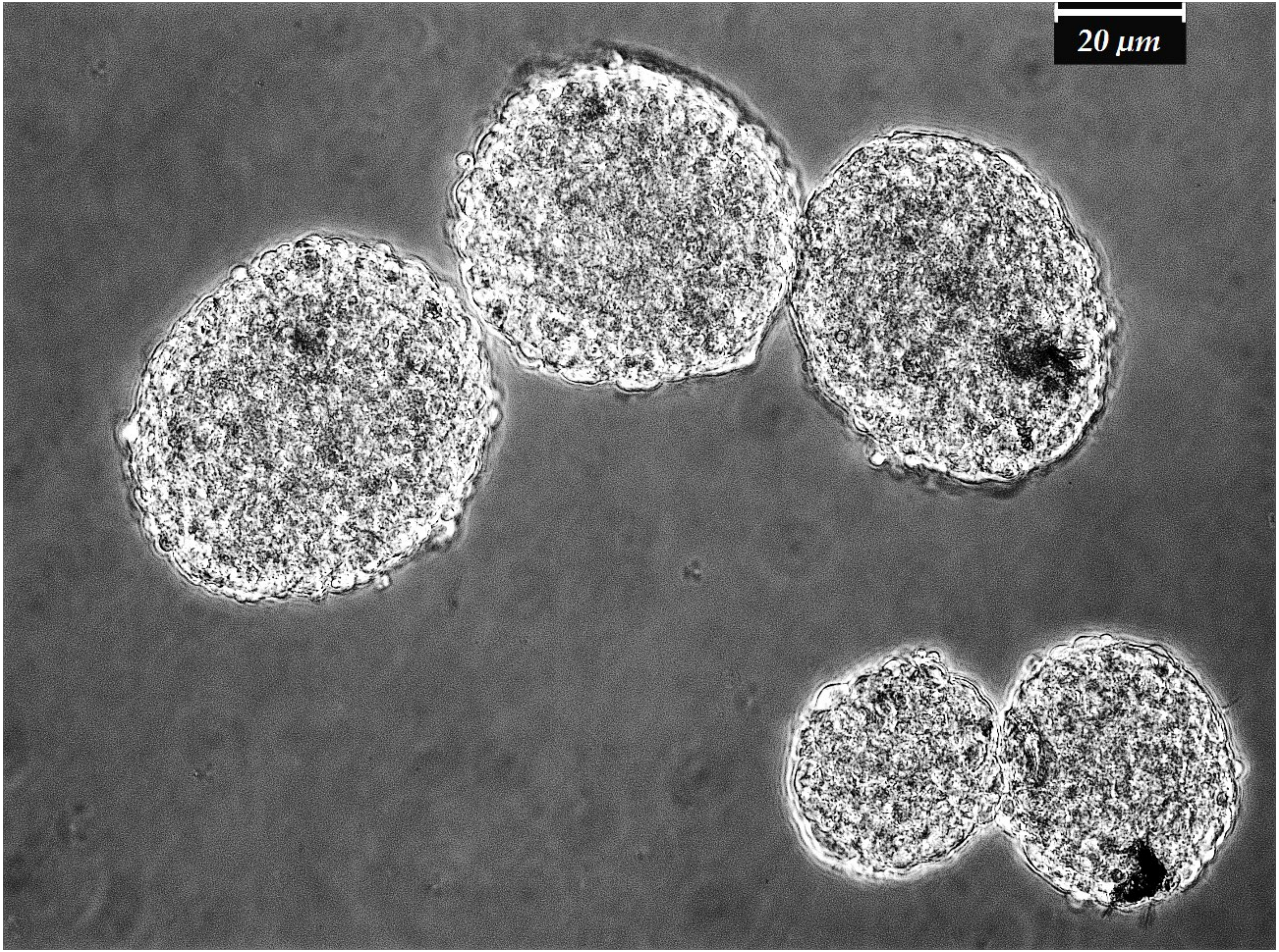

Fig. 3B, d NSC

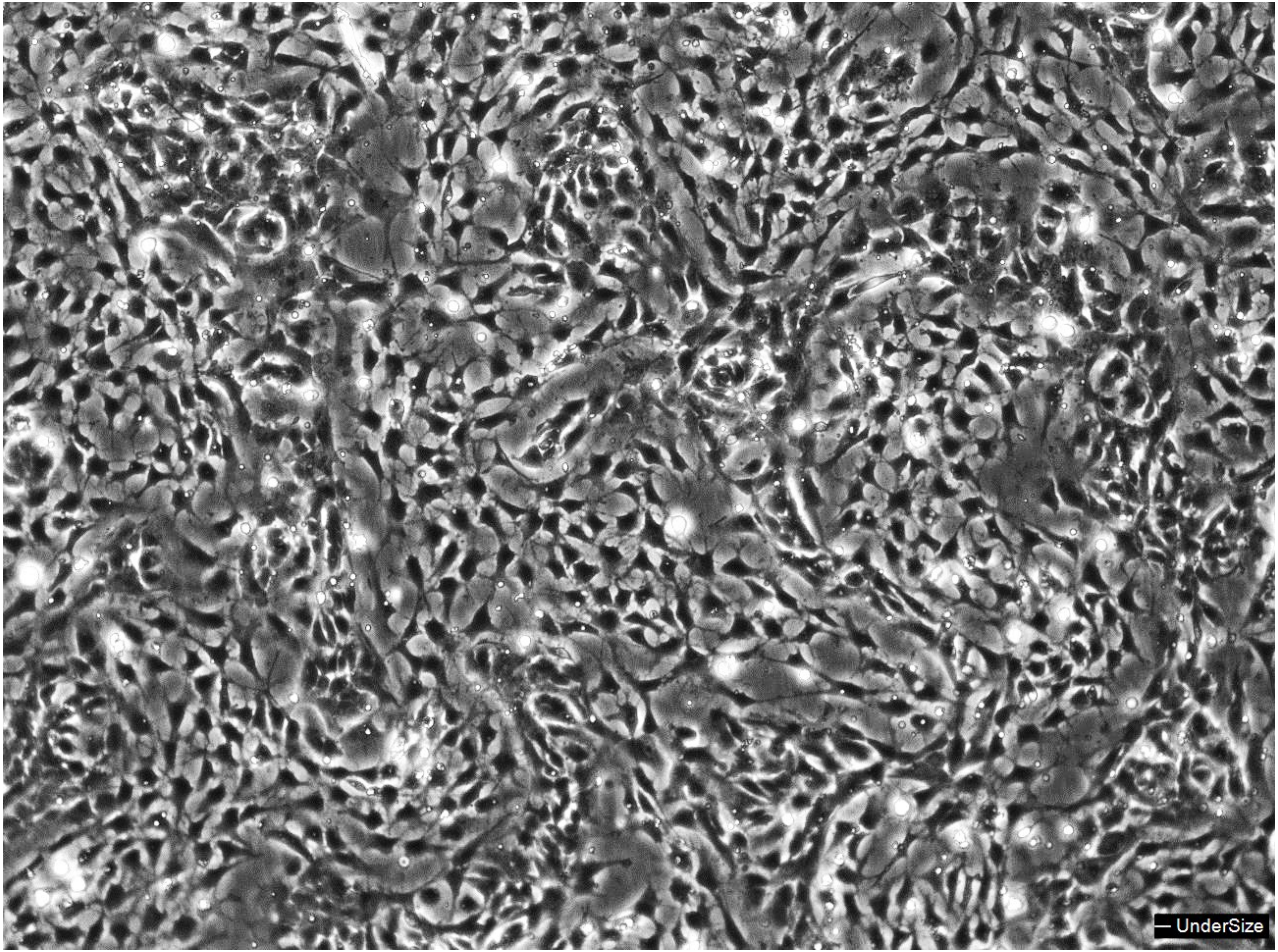

Fig. 3B, e iPSC: SSEA4/POU5F1/DAPI

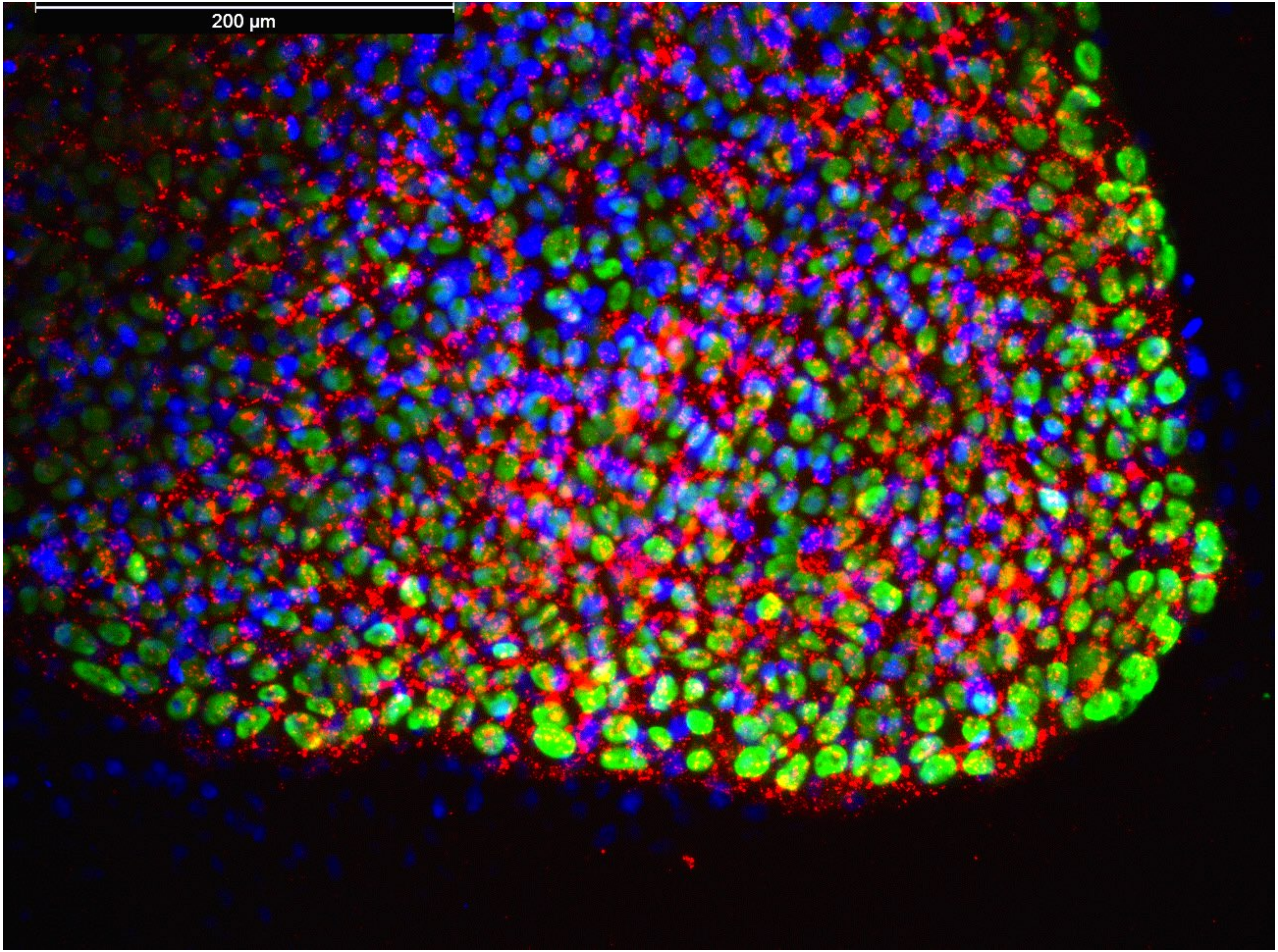

Fig. 3B, f Neuroepithelium: PAX6/NESTIN

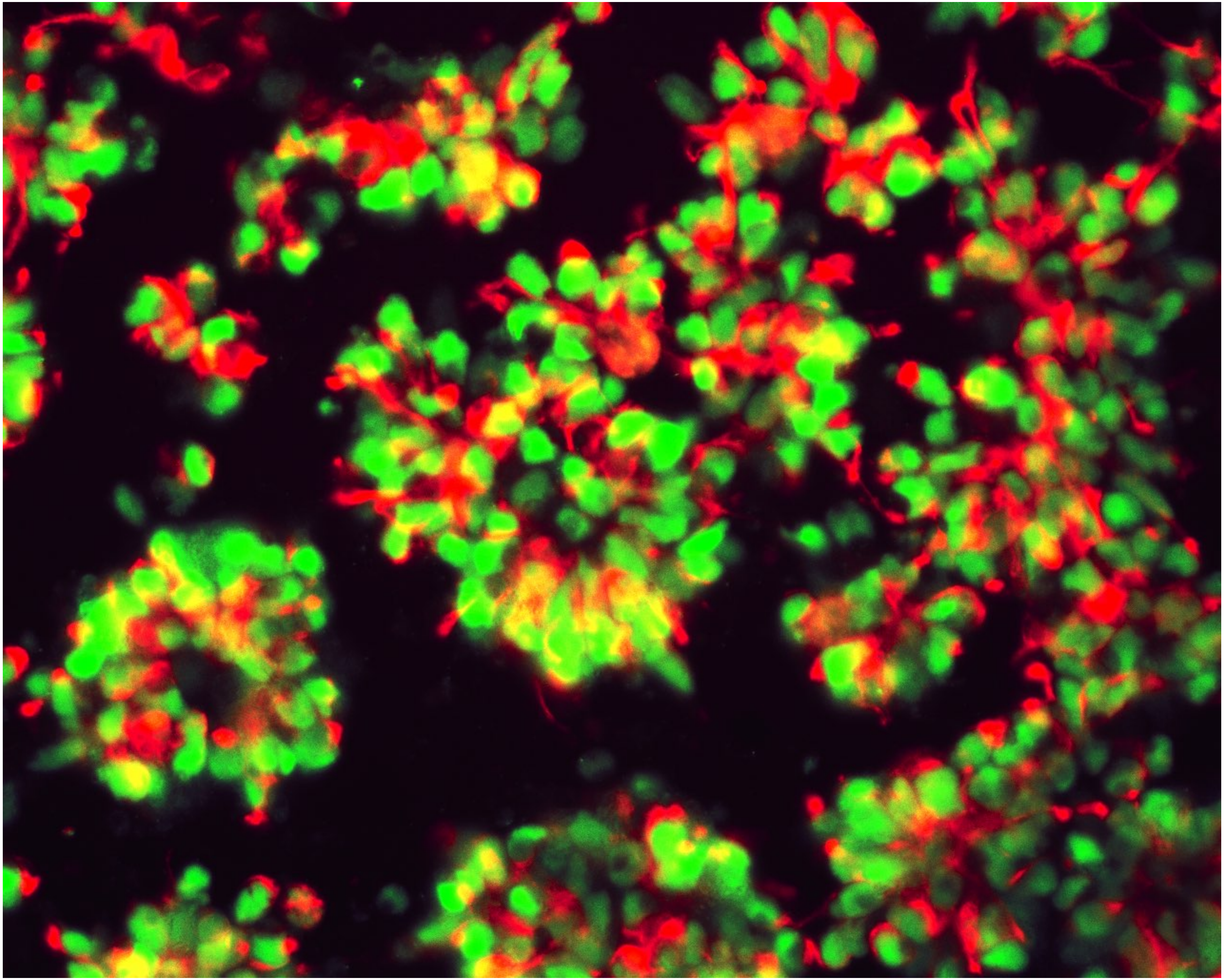

Fig. 3B, g Neurospheres: NESTIN

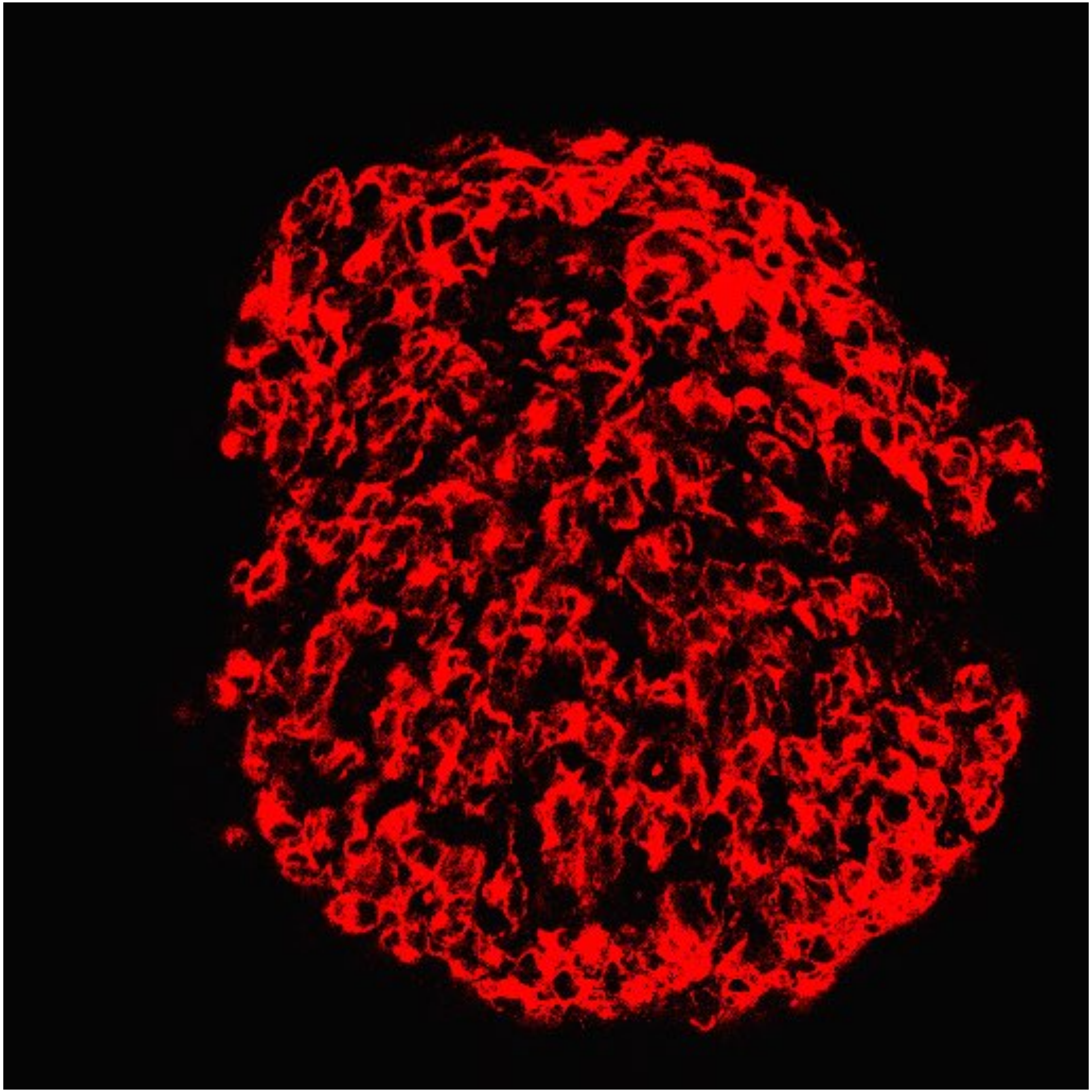

Fig. 3B, h NSC: PAX6

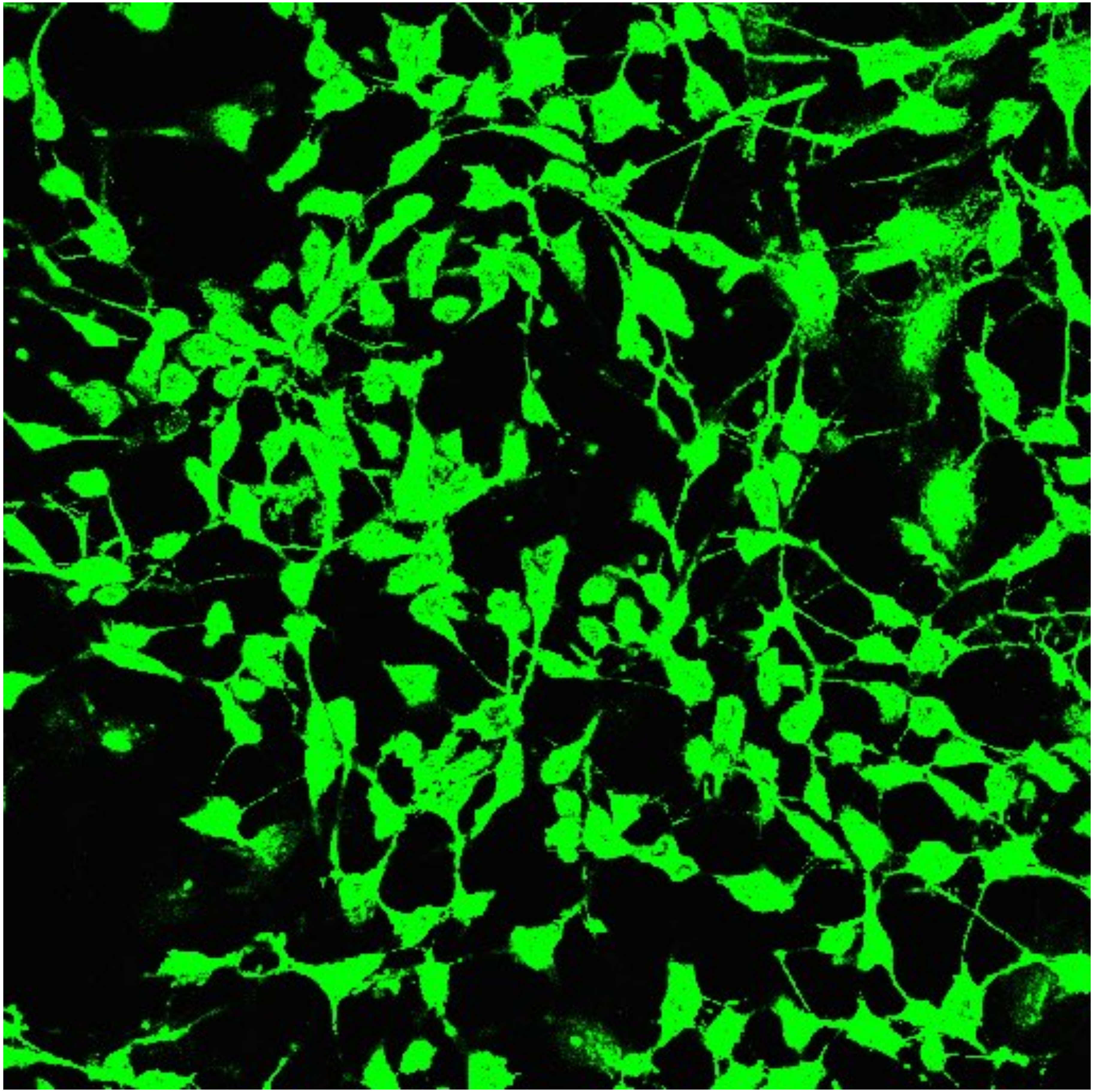

Fig. 3D, DAPI

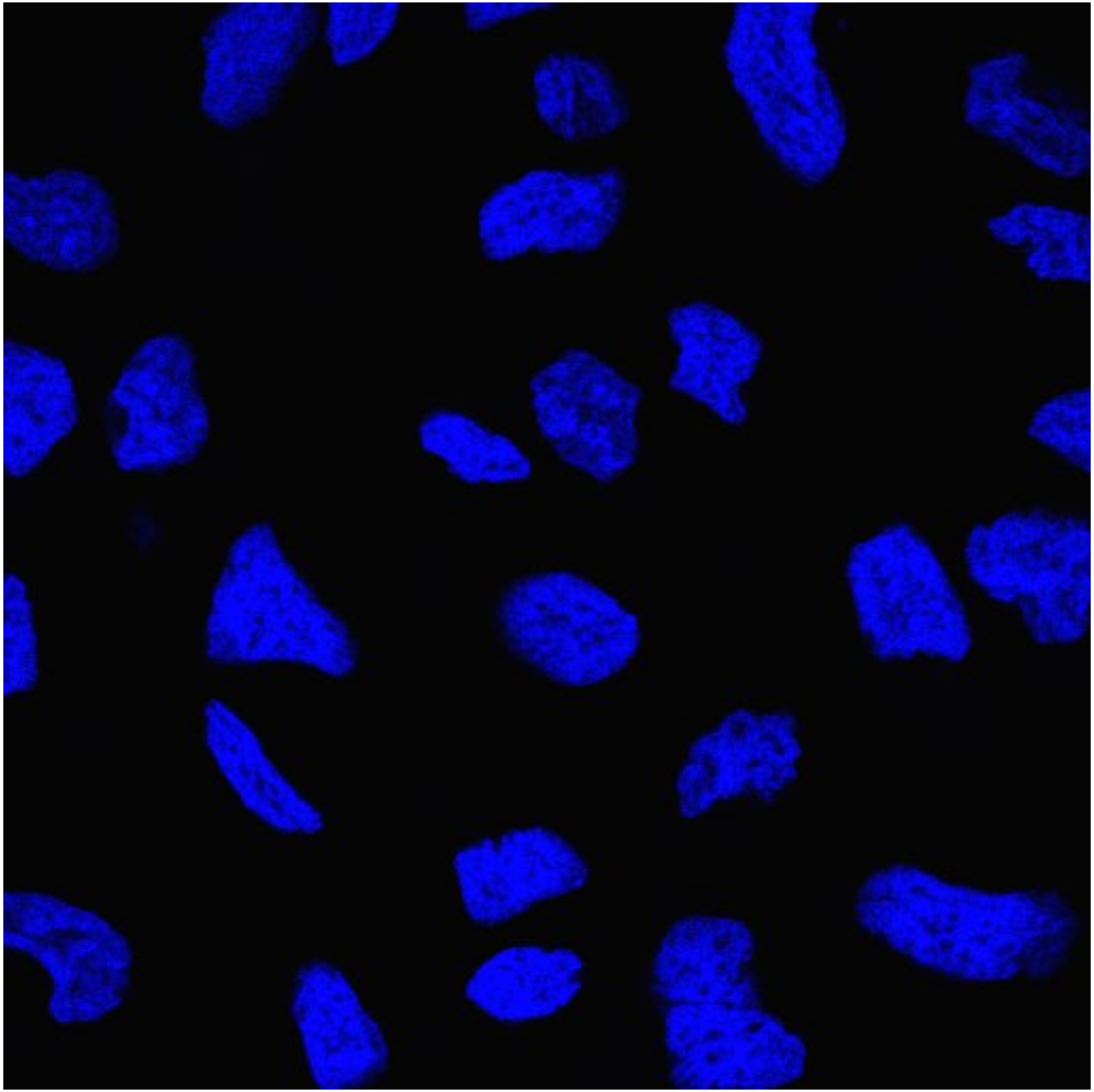

Fig. 3D, PAX6

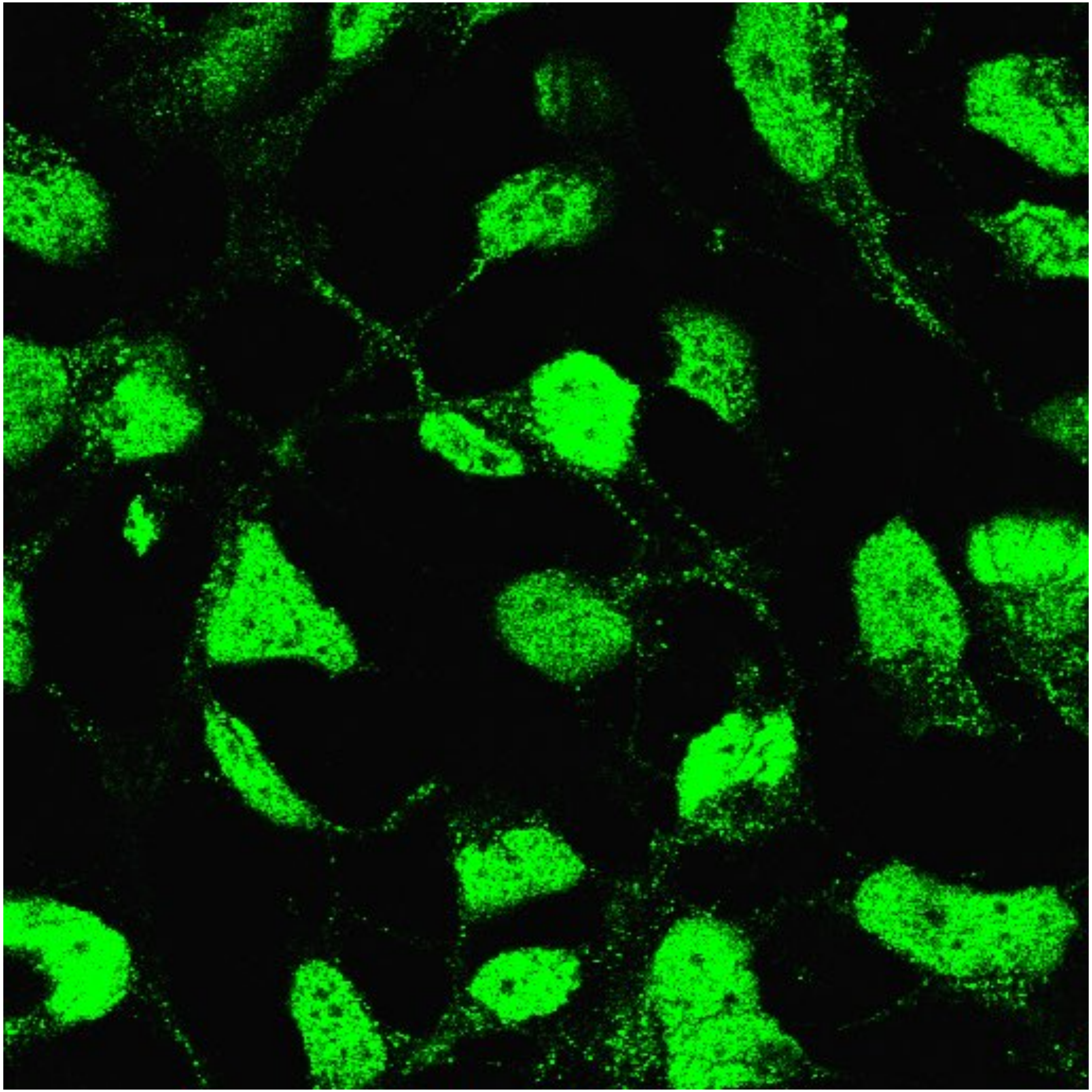

Fig. 3D, NESTIN

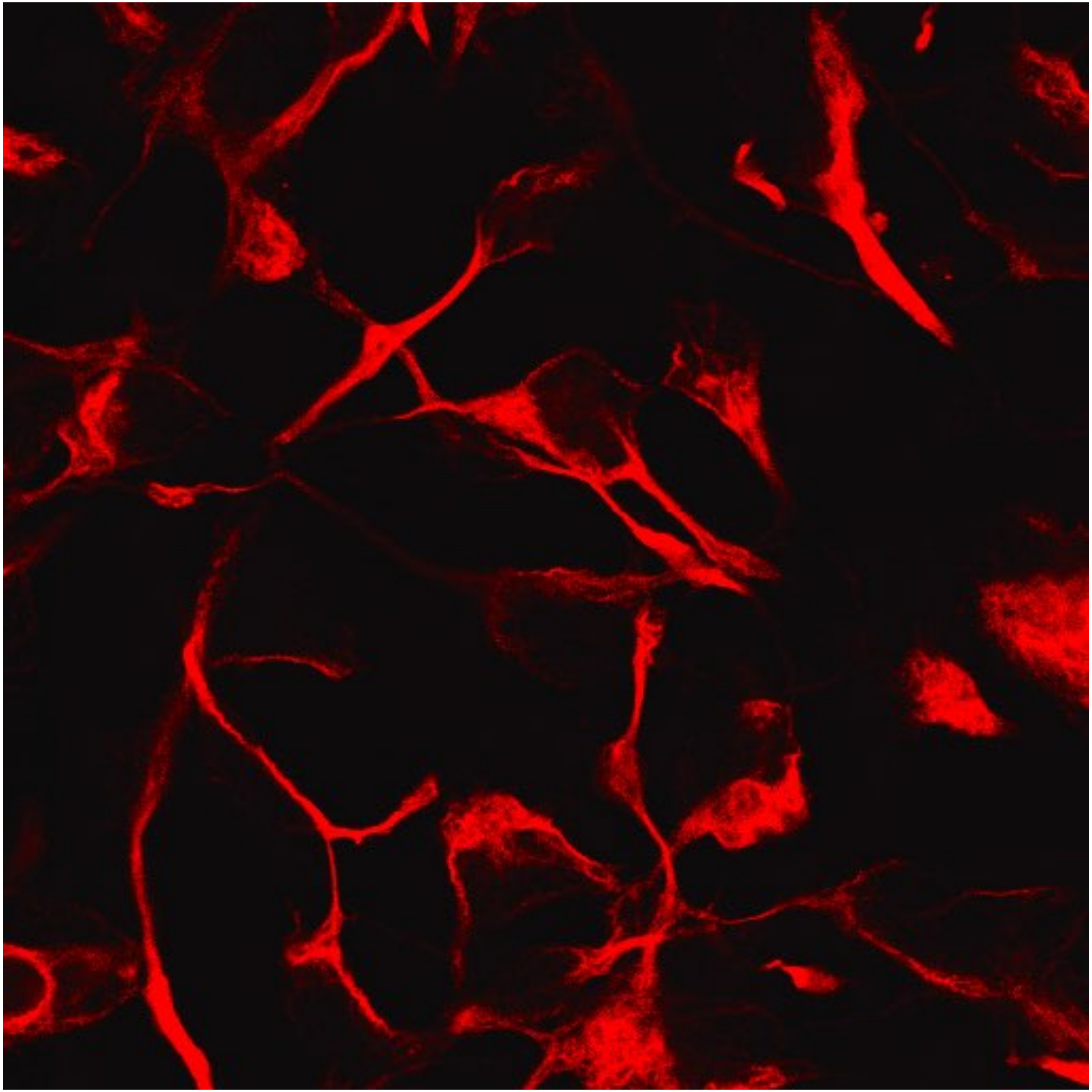

Fig. 3D, MERGE

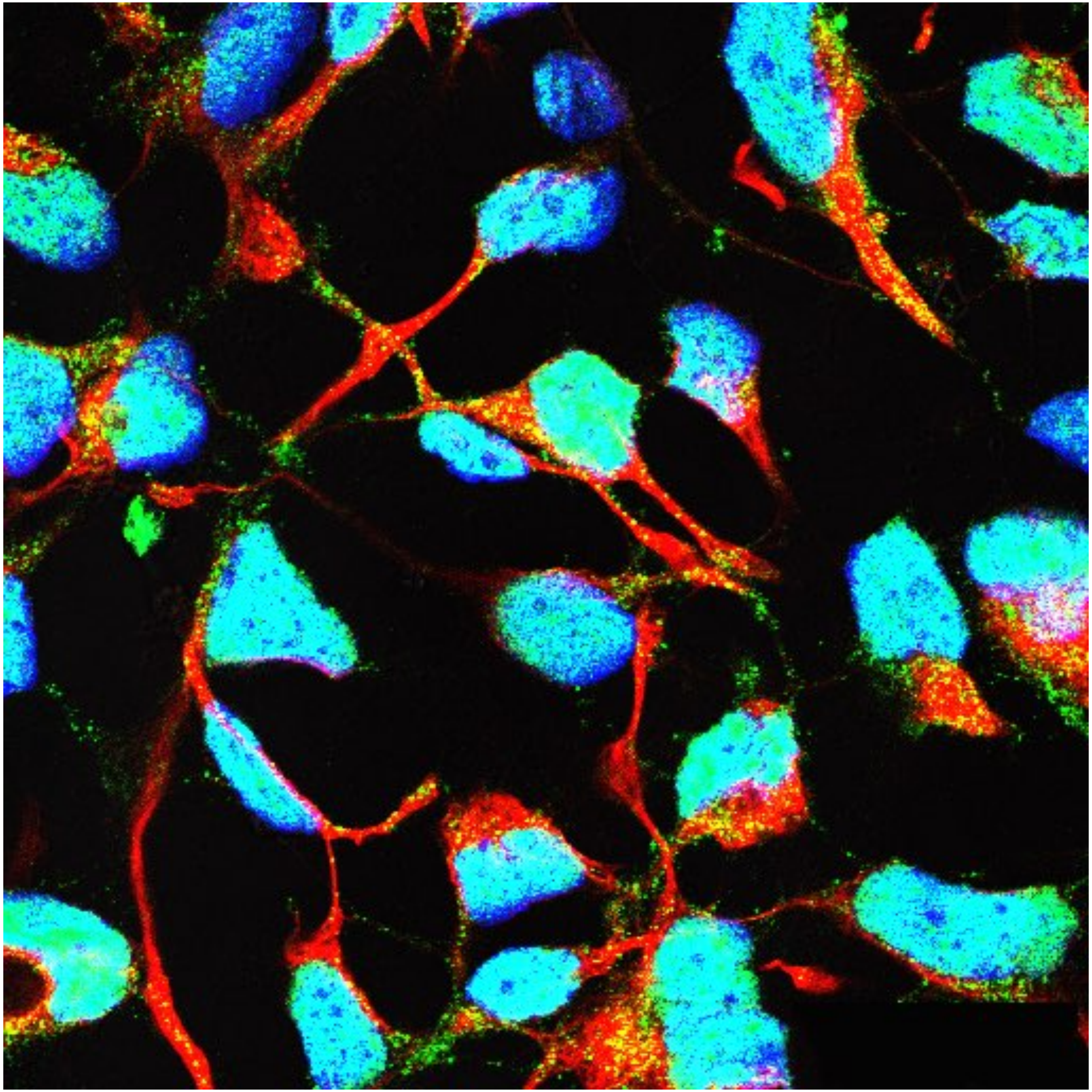

Supplement: Supplementary file 7 — Source Data for Figure 3 [file EMMM-12-e12146-s005.zip › EMM-2020-12146-V5_Source data_Images_Fig. 3.pdf]
