# Supplementary material for: Disease‐specific phenotypes in iPSC‐derived neural stem cells with POLG mutations
Source: EMBO Mol Med. 2020 Aug 25;12(10):e12146. doi: 10.15252/emmm.202012146 (PMC7539330; doi:10.15252/emmm.202012146)

Fig. 7A, a

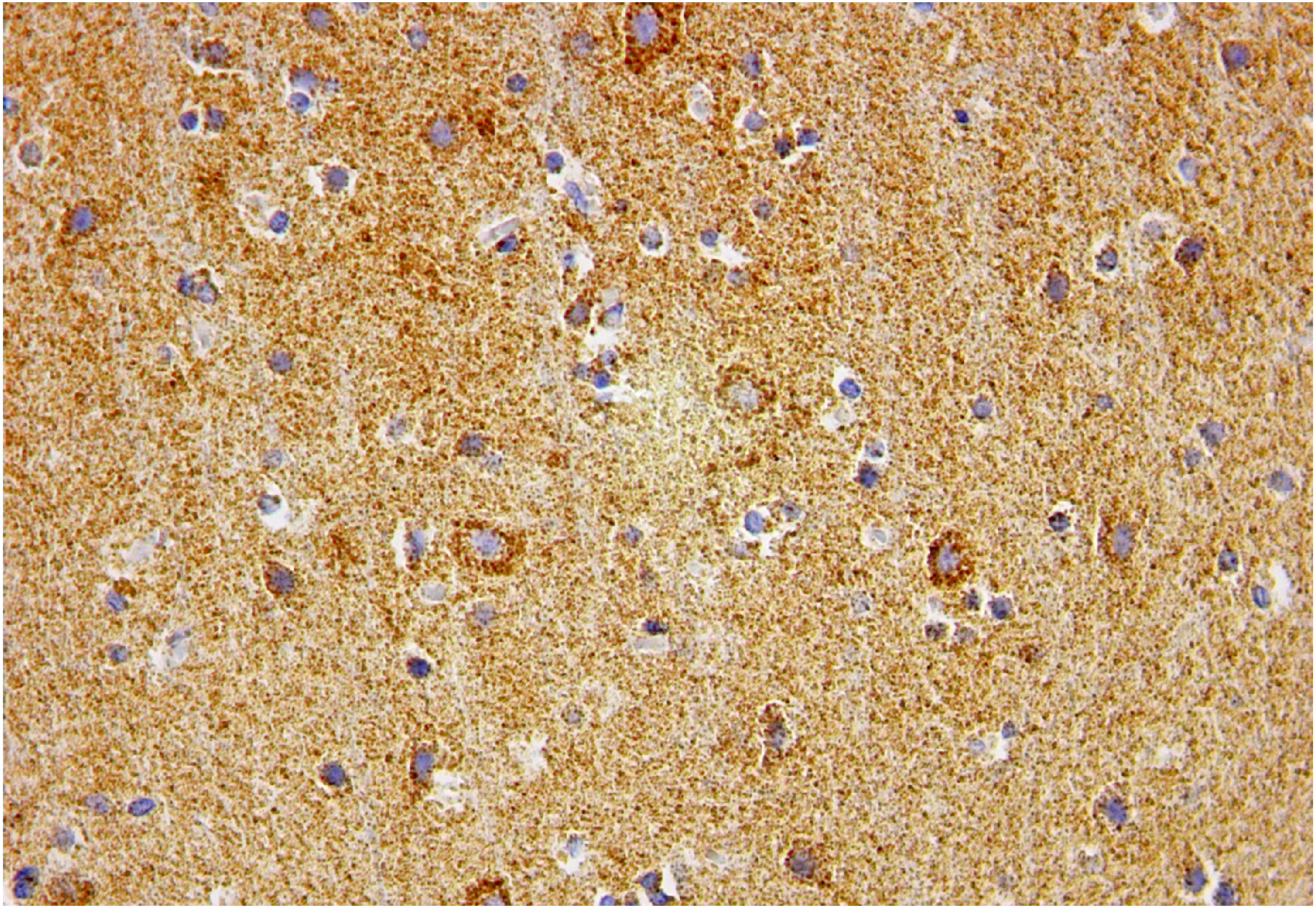

Fig. 7A, b

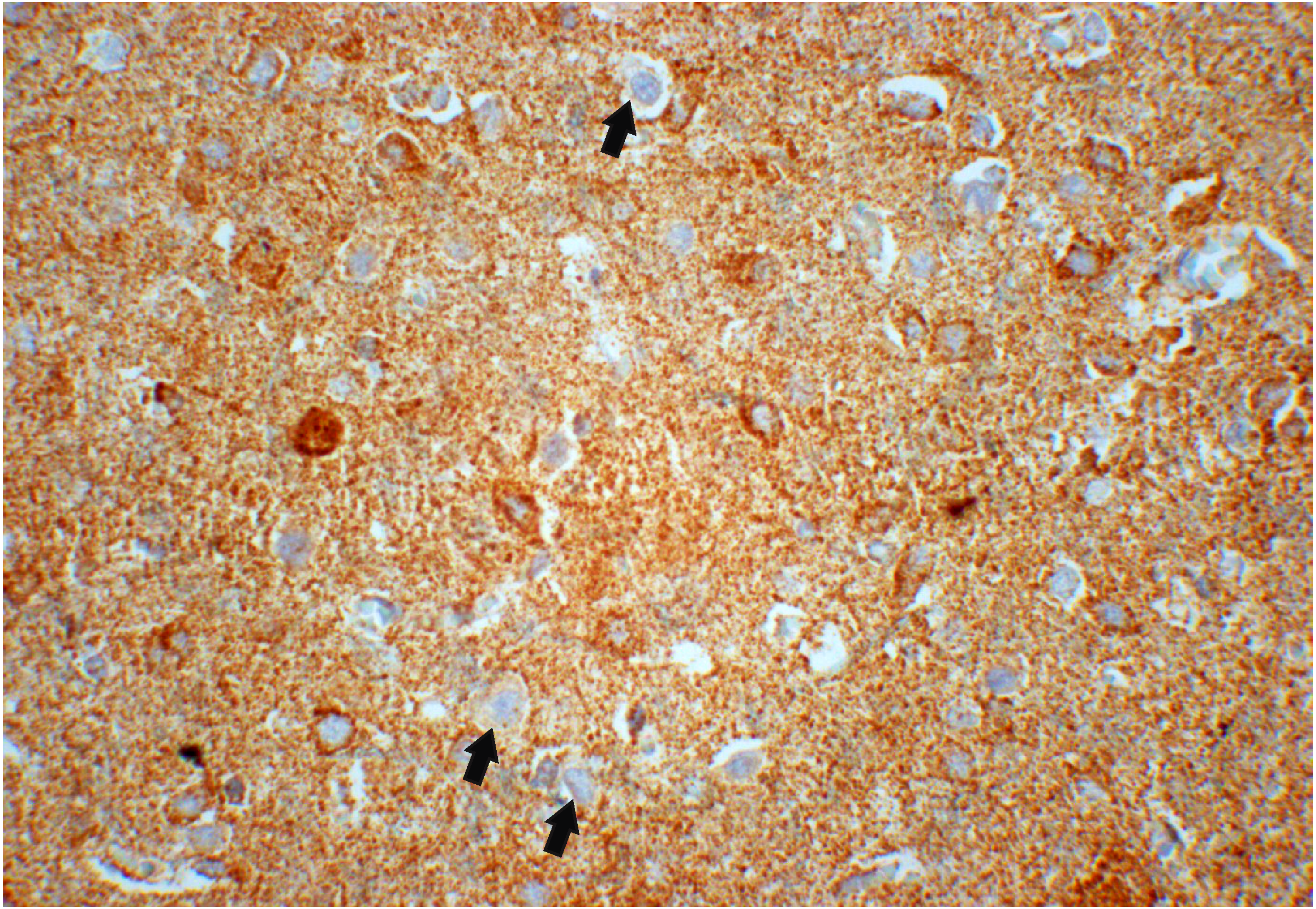

Fig. 7A, c

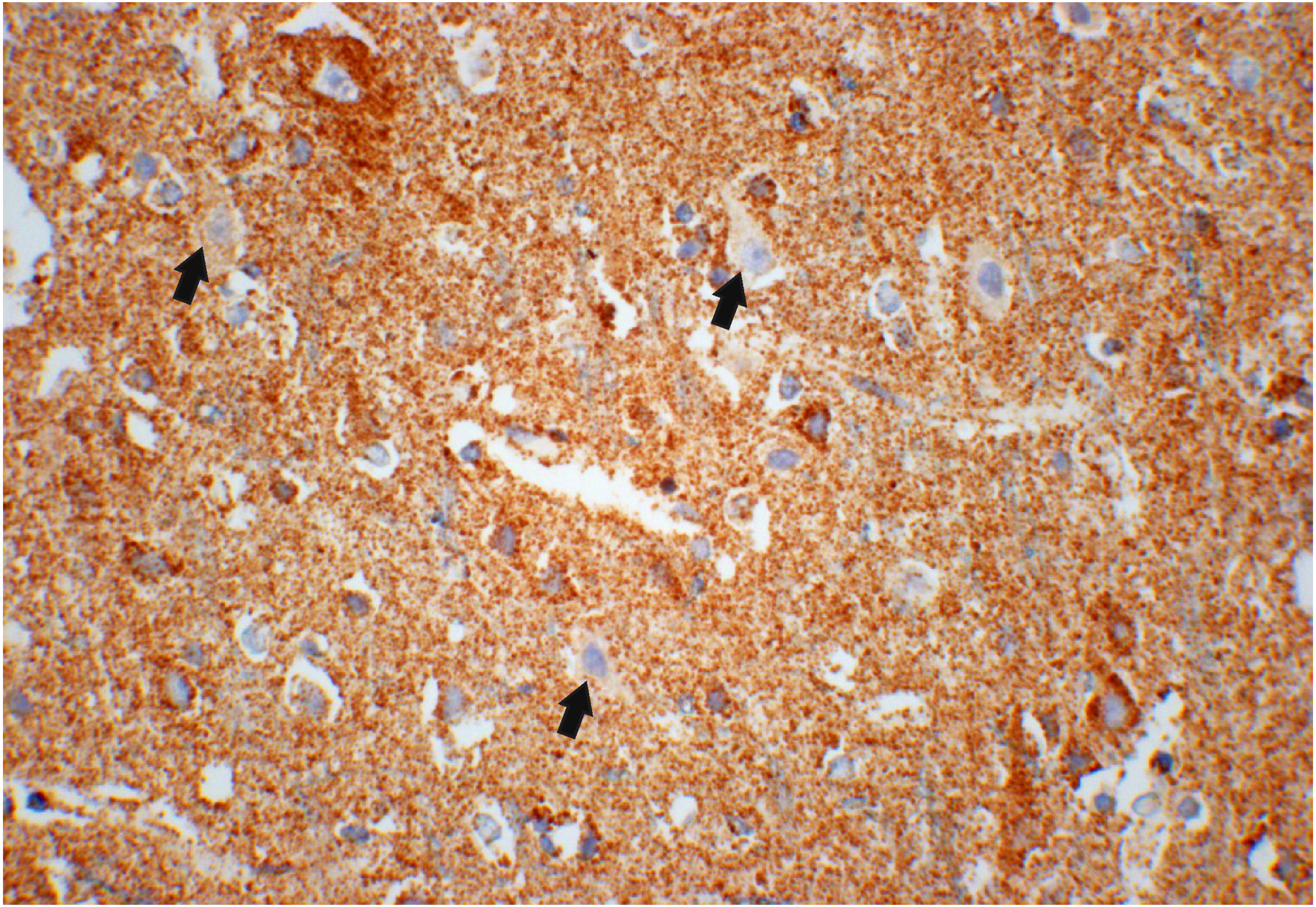

Fig. 7B CTRL NSC NDUFB10

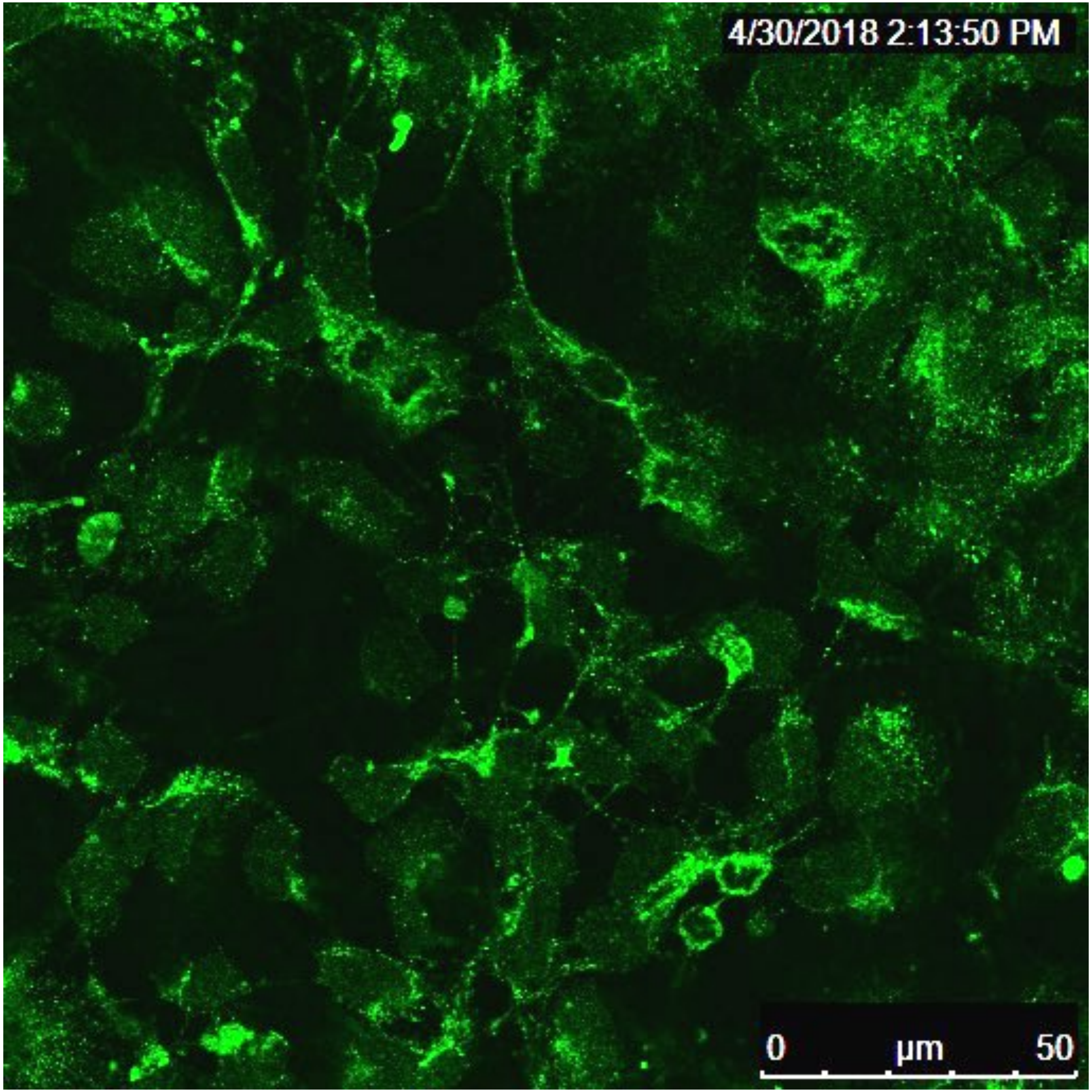

Fig. 7B CTRL NSC TOMM20

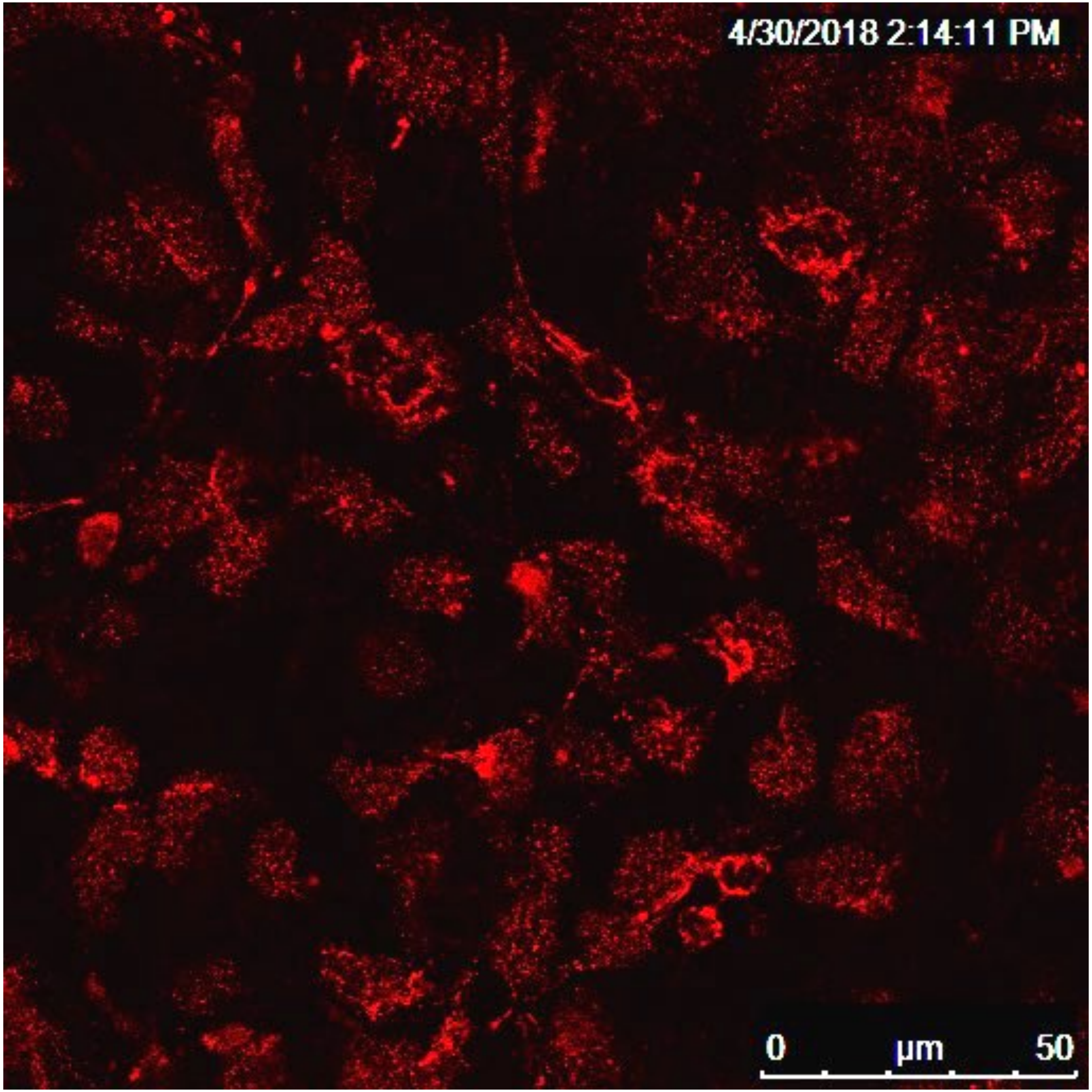

Fig. 7B CTRL NSC DAPI

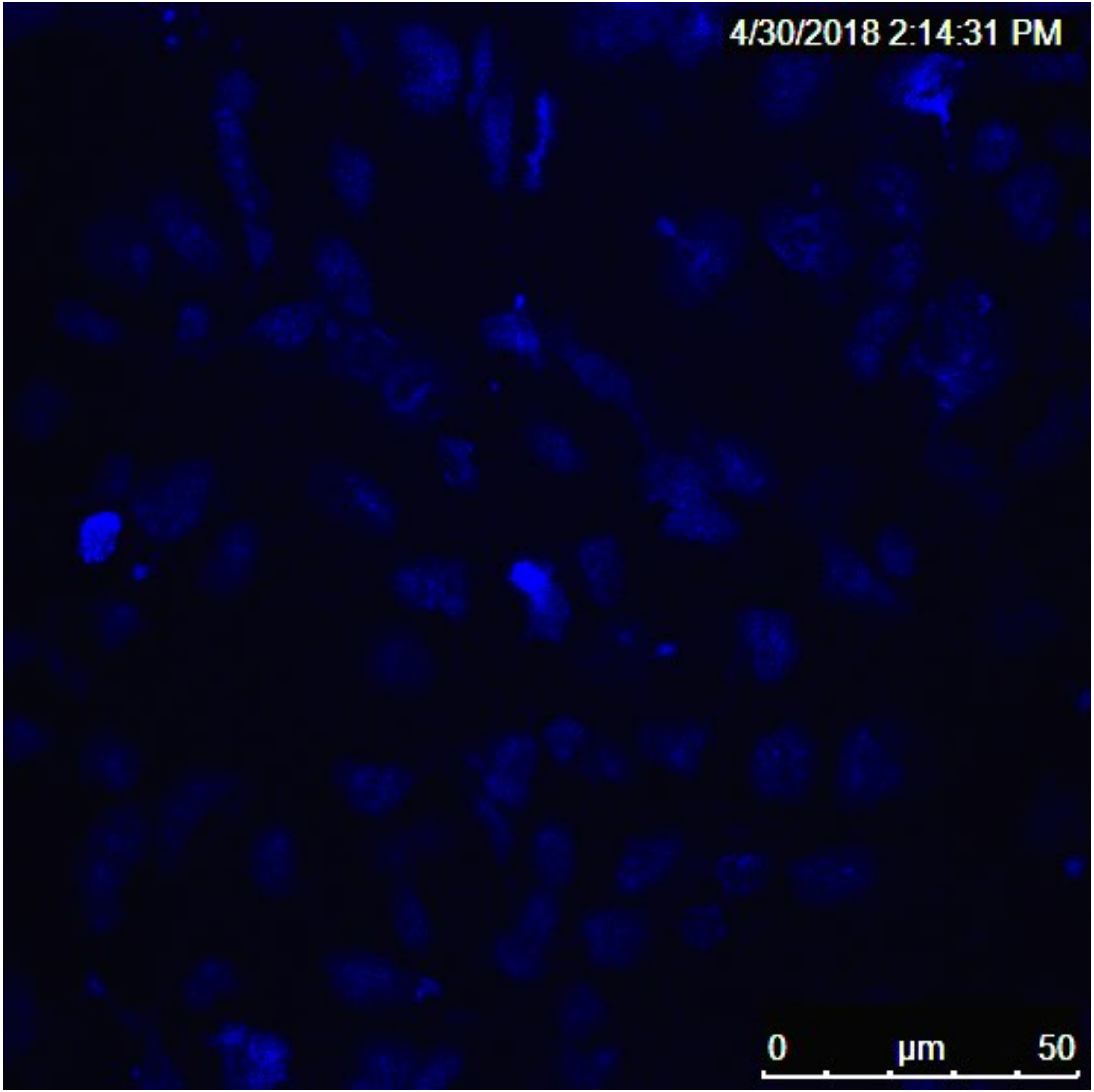

Fig. 7B CTRL NSC MERGE

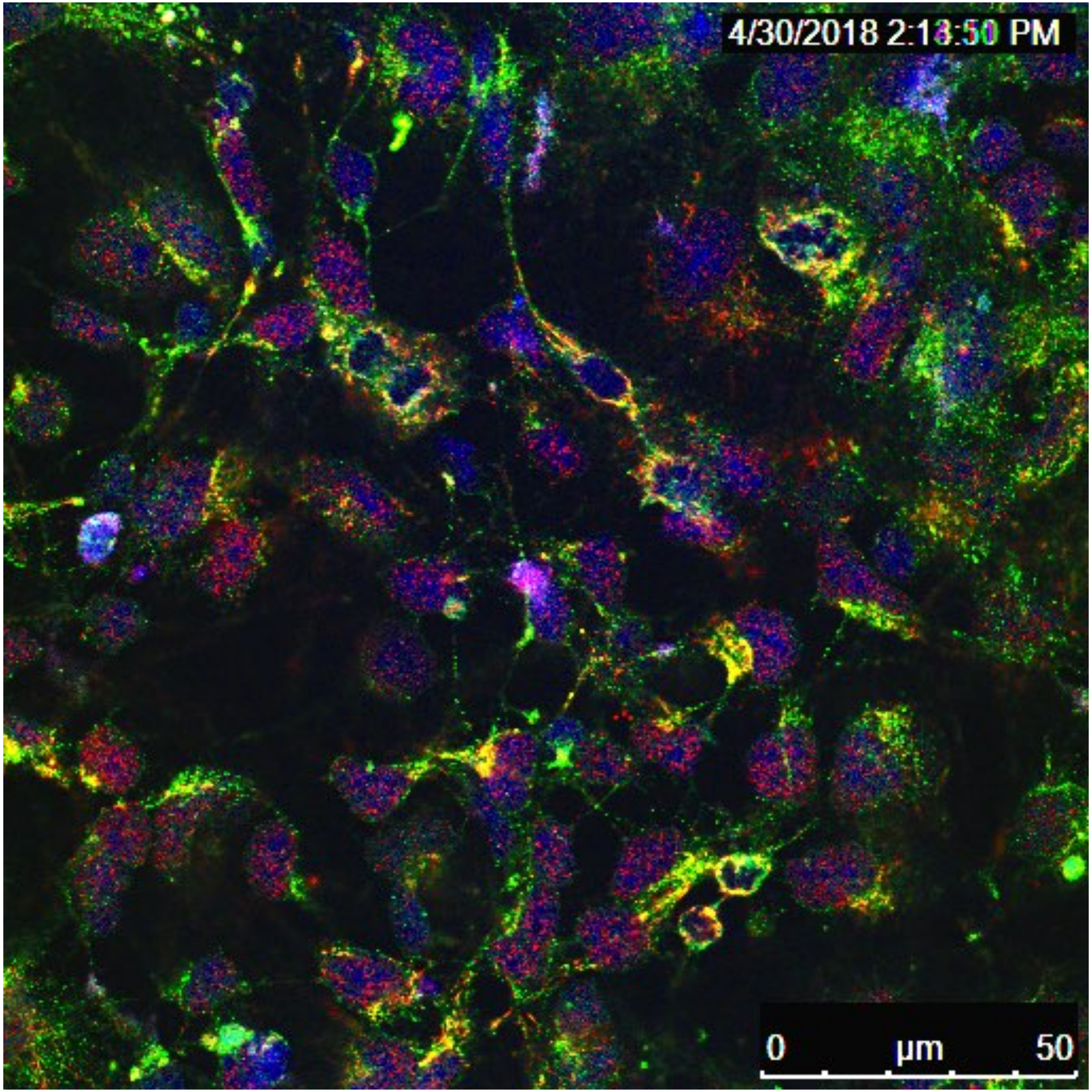

Fig. 7B WS5A NSC NDUFB10

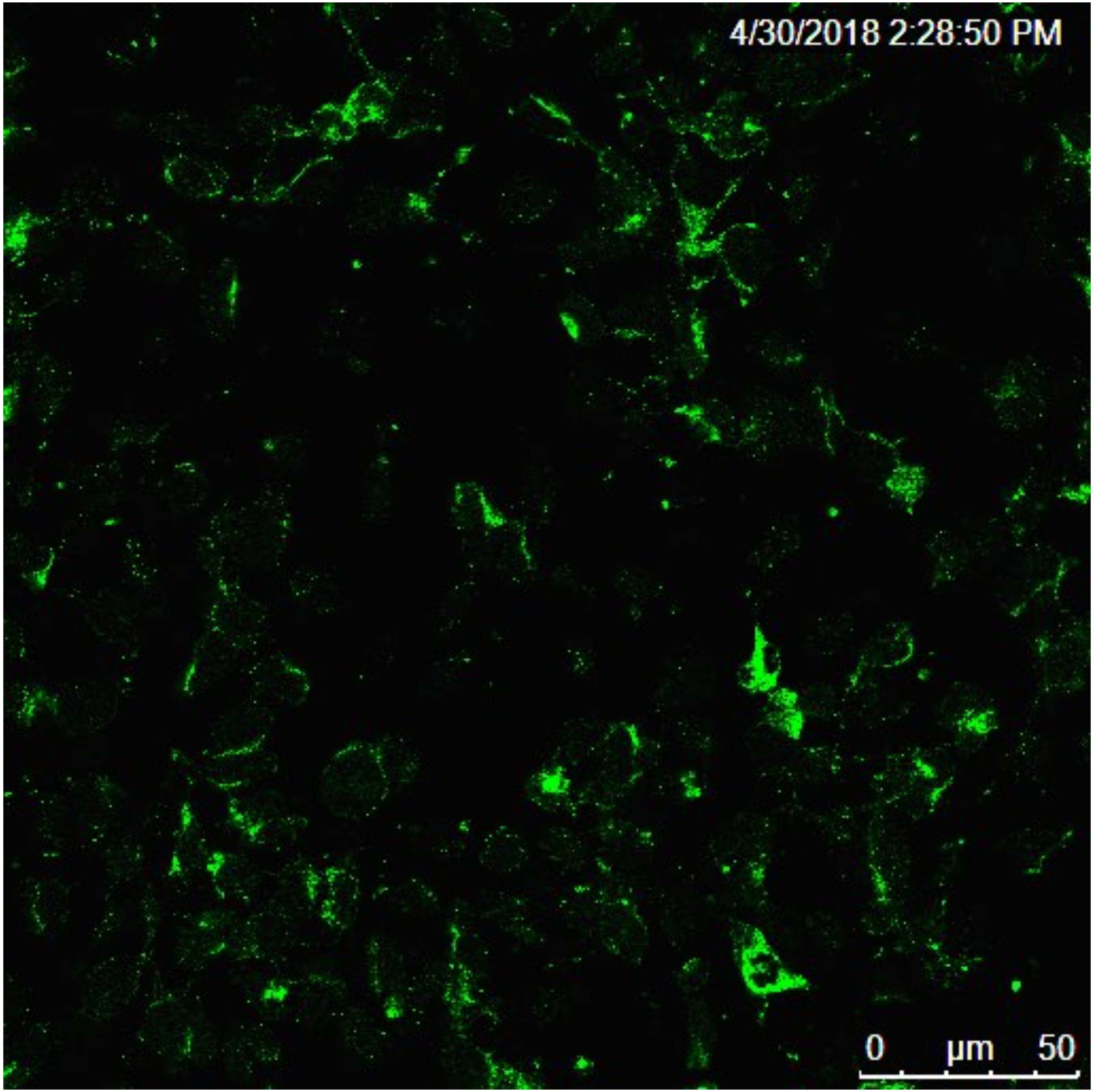

Fig. 7B WS5A NSC TOMM20

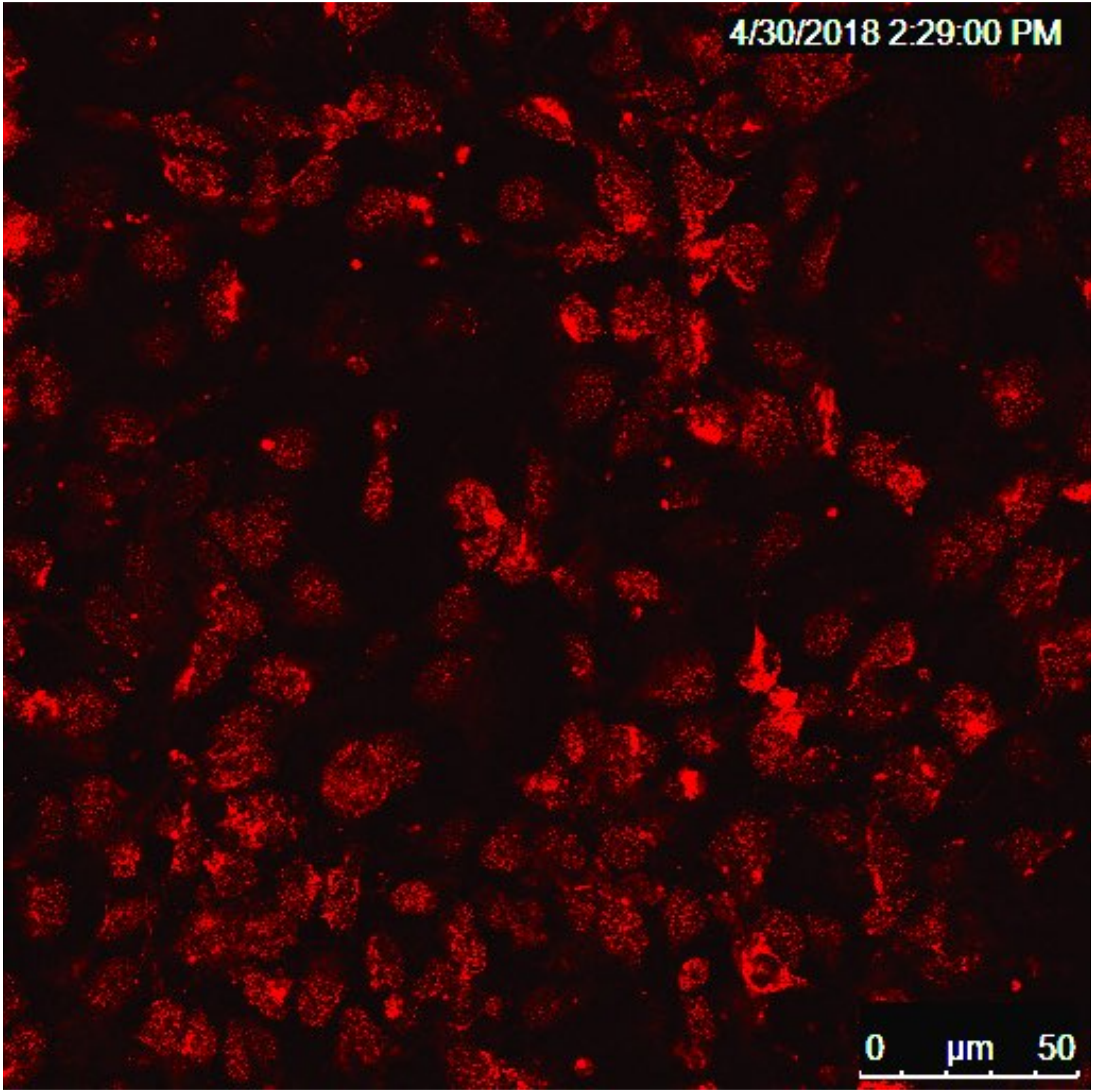

Fig. 7B WS5A NSC DAPI

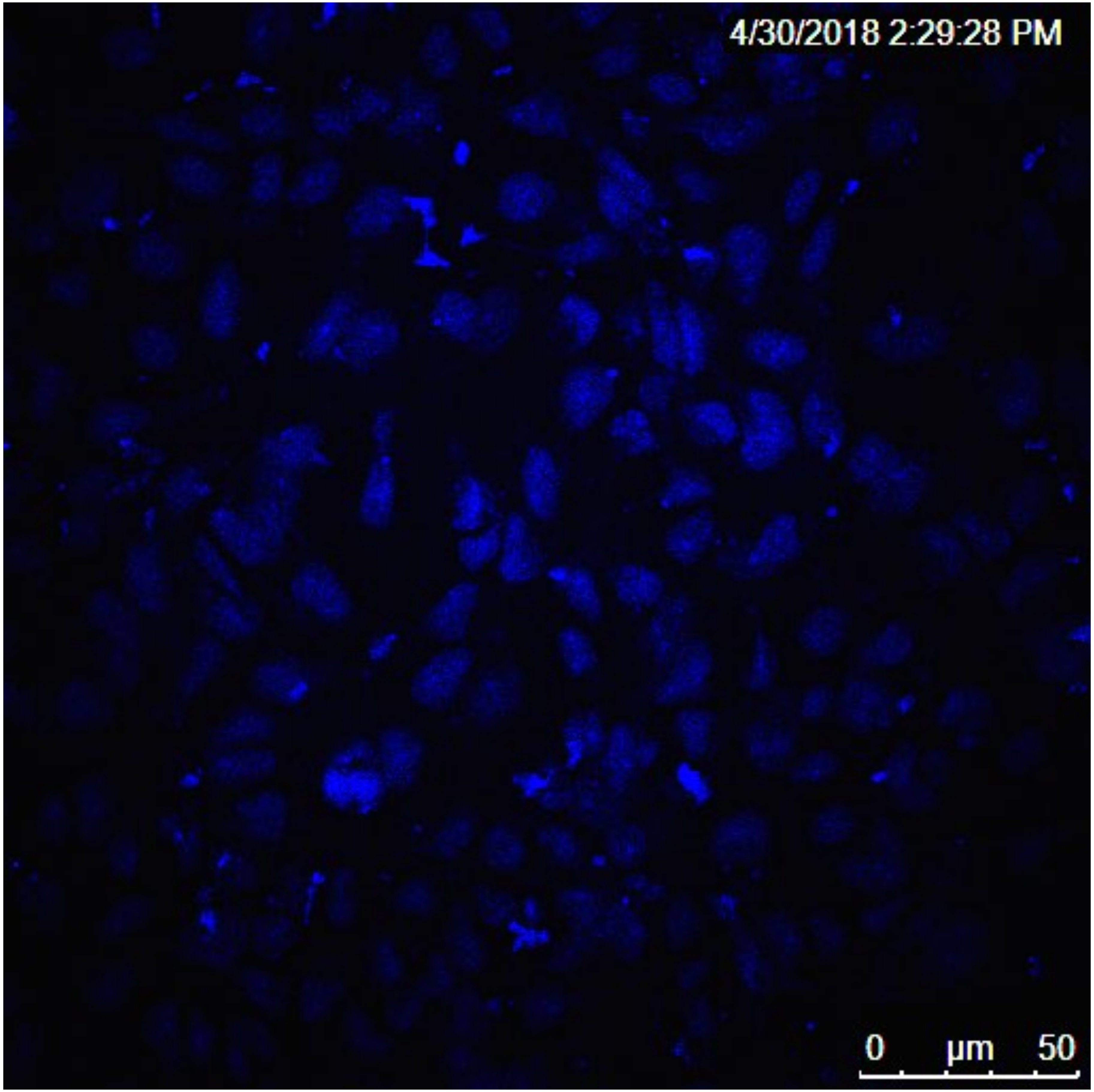

Fig. 7B WS5A NSC MERGE

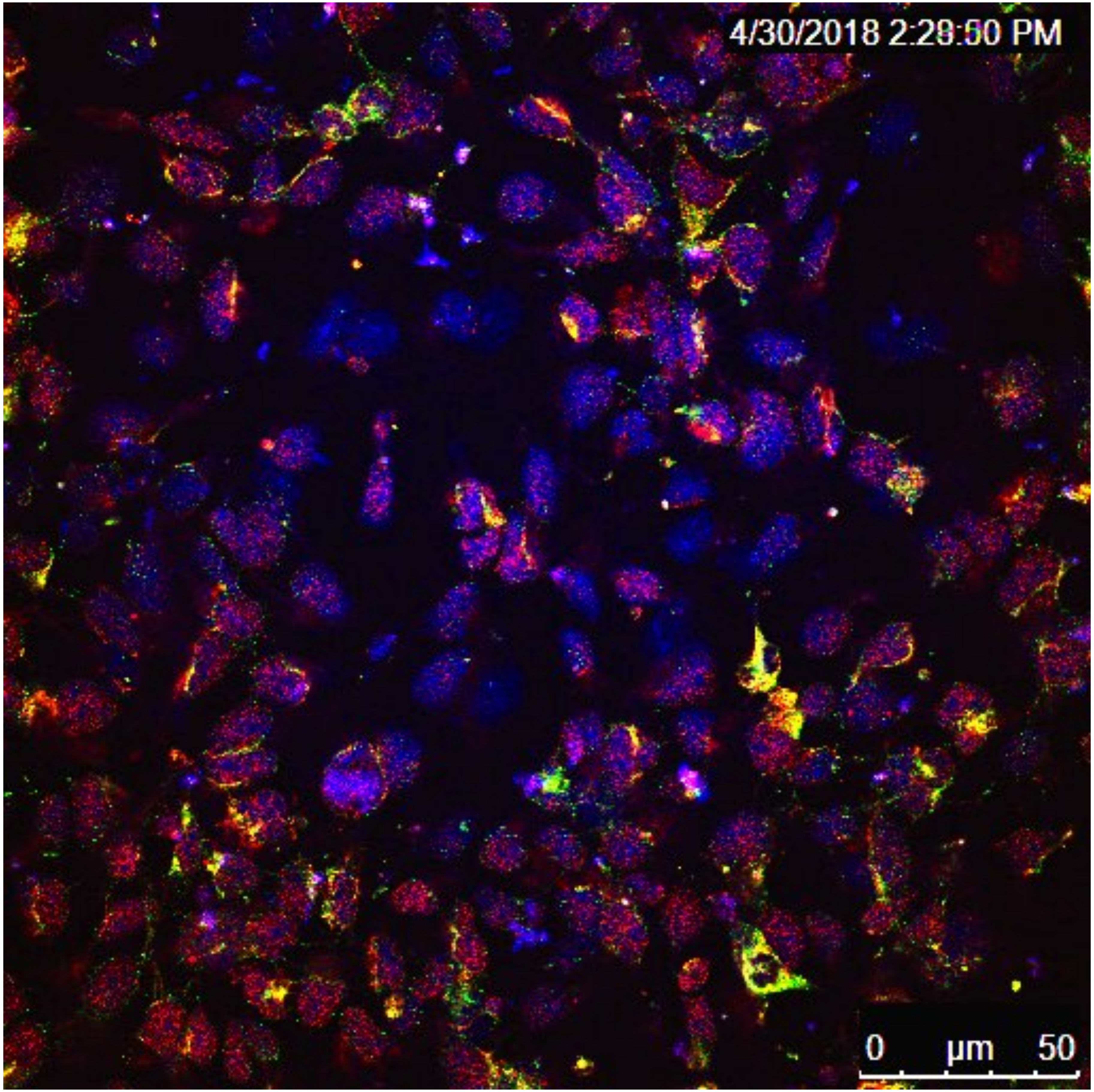

Fig. 7B CP2A NSC NDUFB10

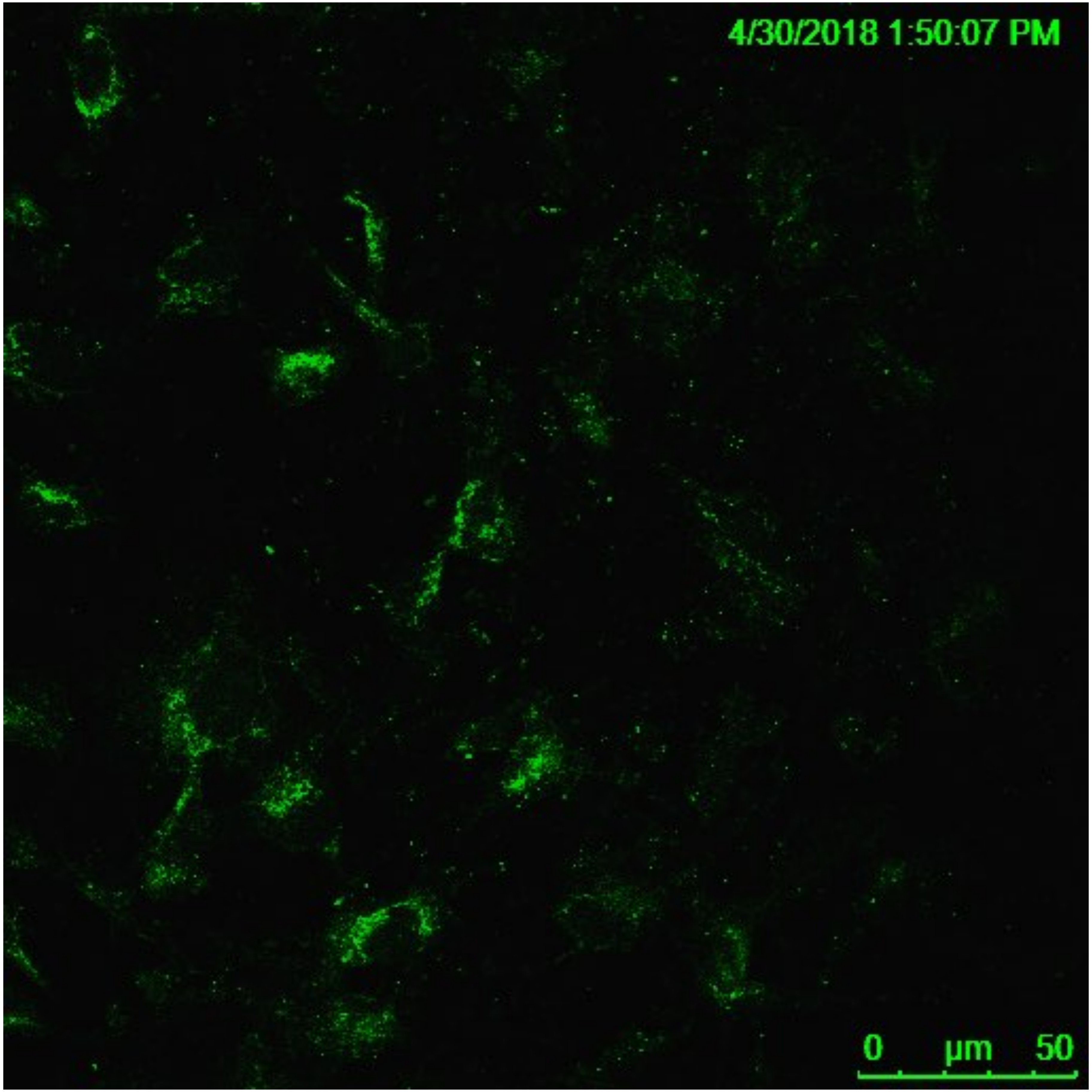

Fig. 7B CP2A NSC TOMM20

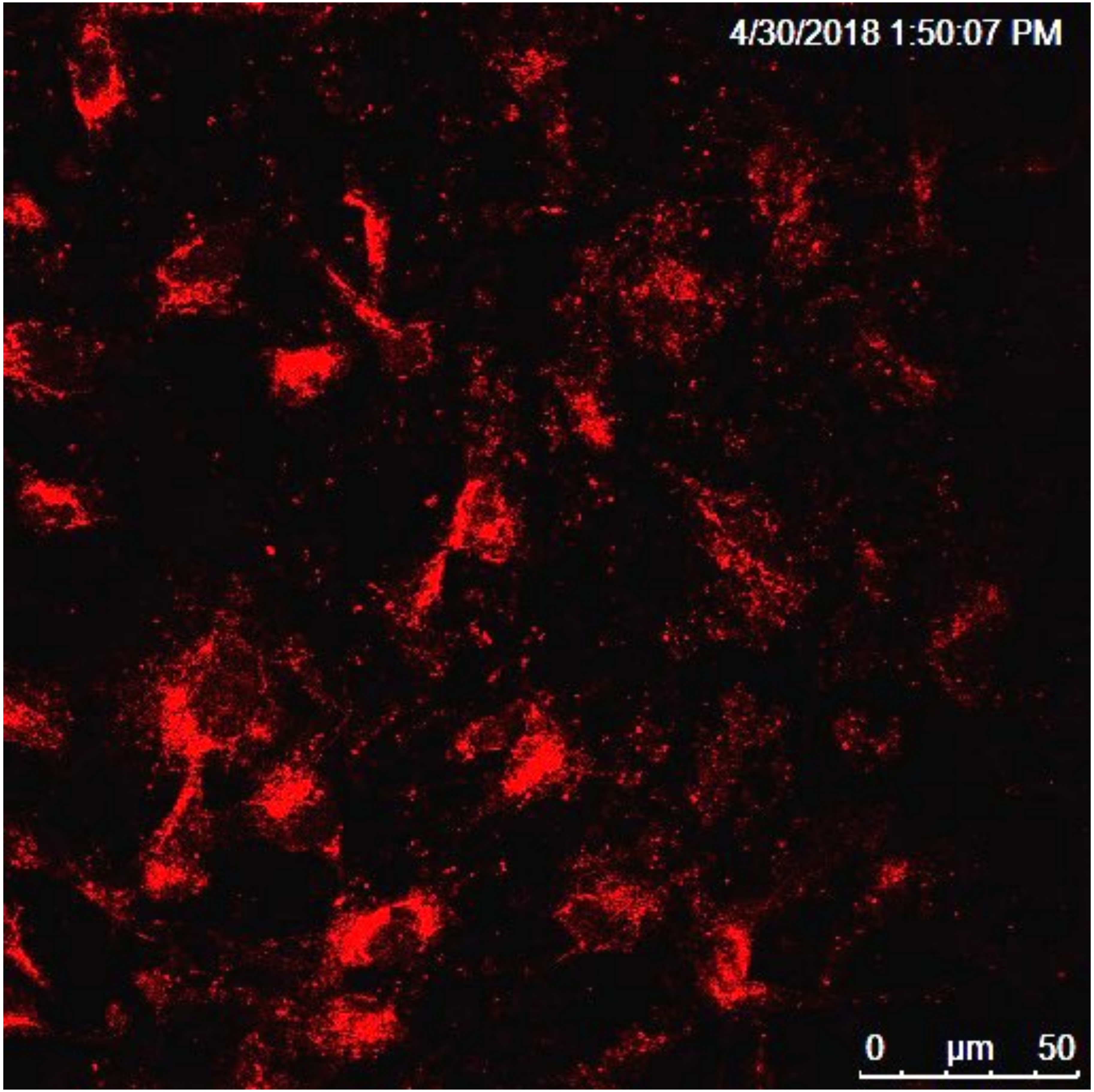

Fig. 7B CP2A NSC DAPI

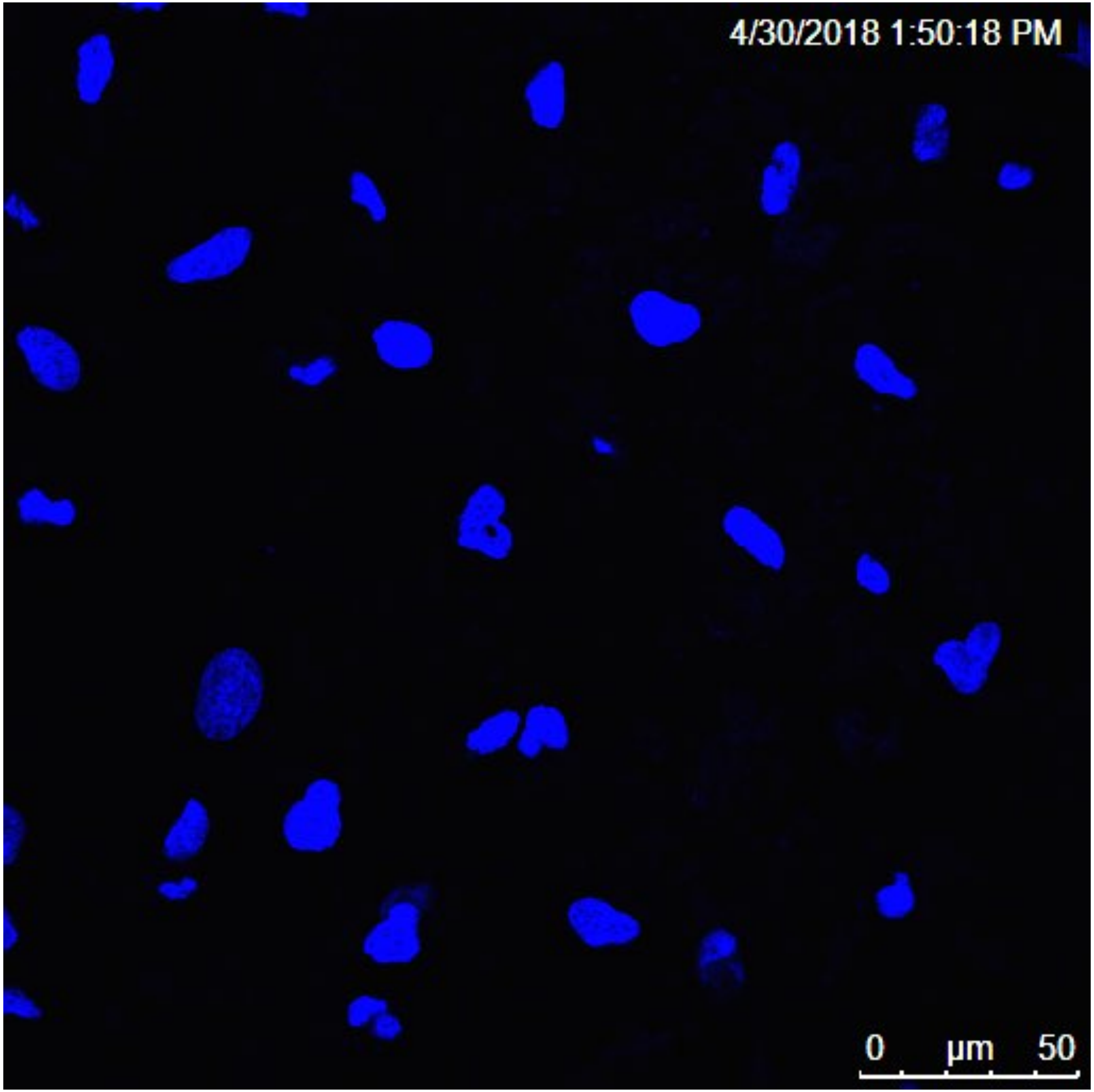

Fig. 7B CP2A NSC MERGE

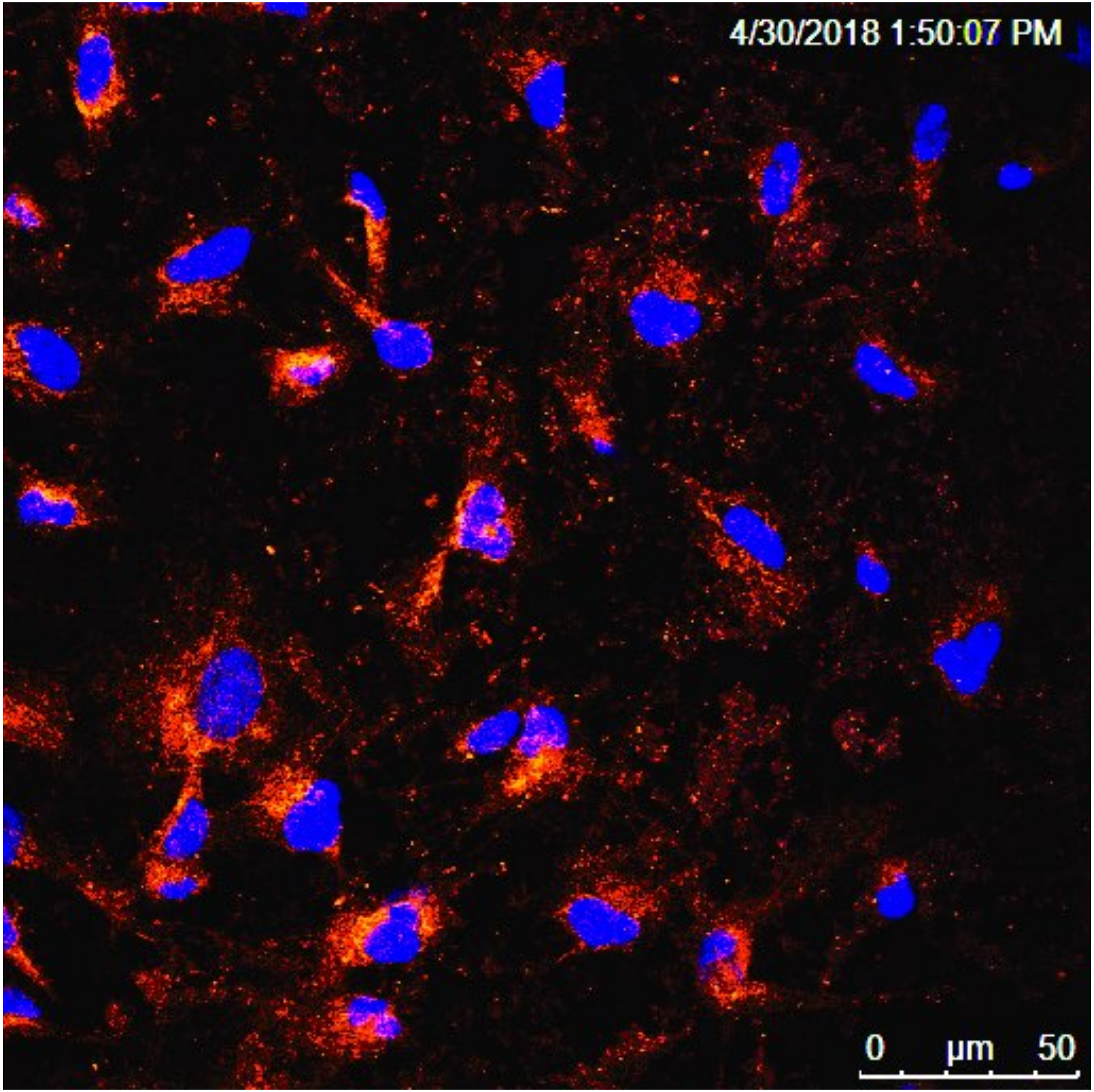

Supplement: Supplementary file 11 — Source Data for Figure 7 [file EMMM-12-e12146-s009.zip › EMM-2020-12146-V5_Source data_Images_Figure 7.pdf]
