# Supplementary material for: Disease‐specific phenotypes in iPSC‐derived neural stem cells with POLG mutations
Source: EMBO Mol Med. 2020 Aug 25;12(10):e12146. doi: 10.15252/emmm.202012146 (PMC7539330; doi:10.15252/emmm.202012146)
Supplement: Supplementary file 12 — Source Data for Figure 8 [file EMMM-12-e12146-s010.zip › EMM-2020-12146-V5_Source data_Images_Fig. 8.pdf]

Fig. 8I CTRL NSC

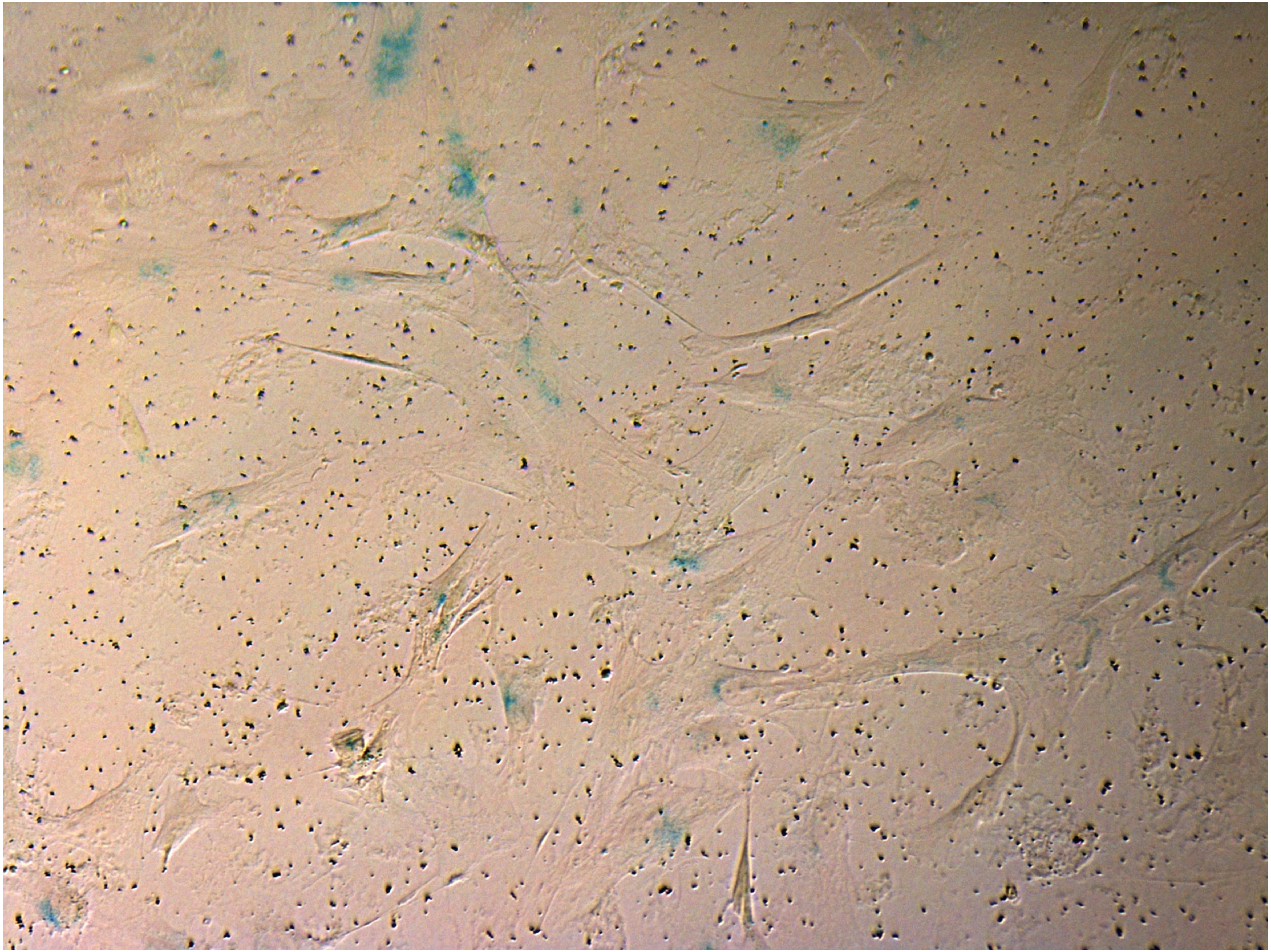

Fig. 8I WS5A NSC

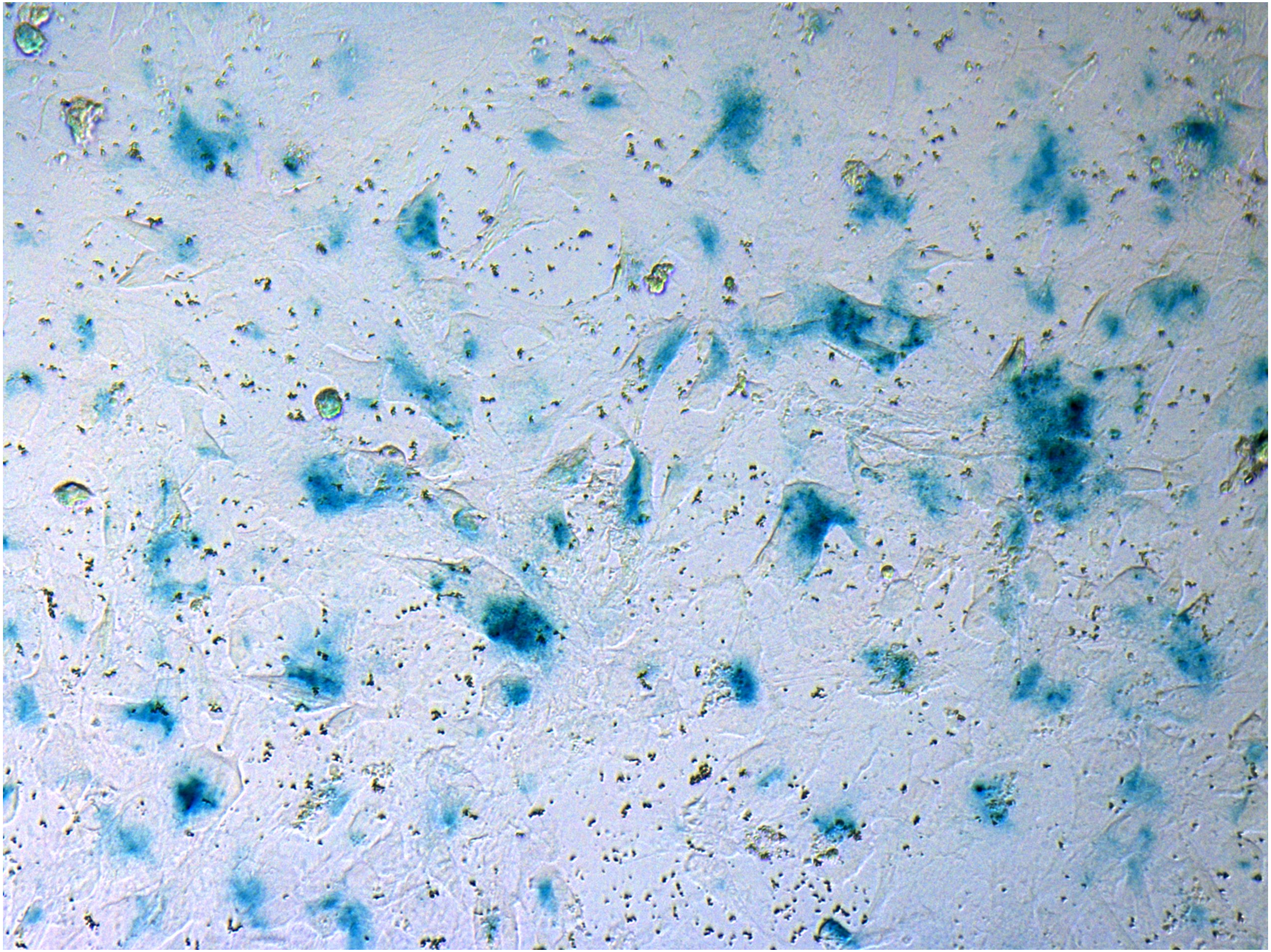

Fig. 8I CP2A NSC

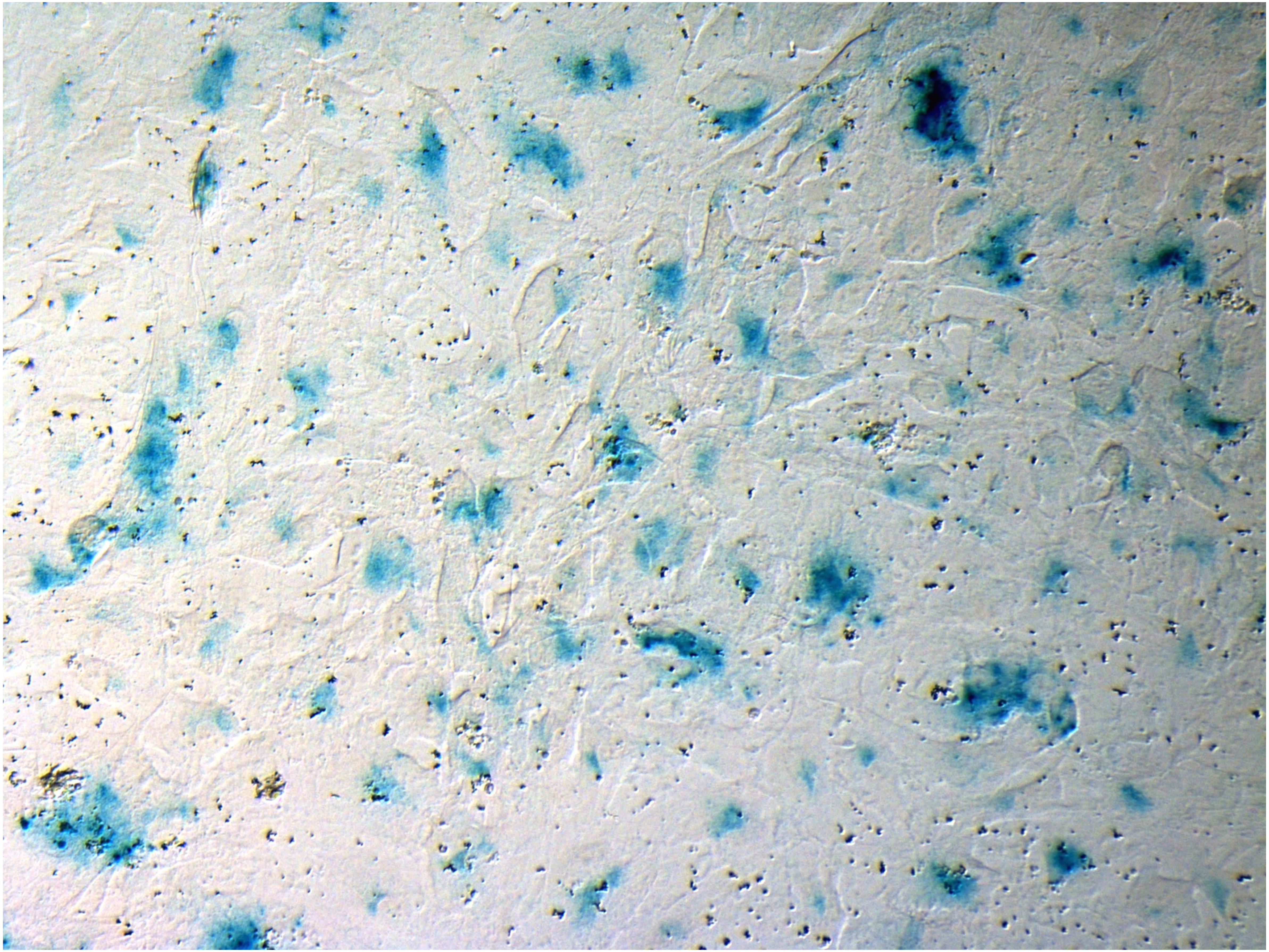

Fig. 8K

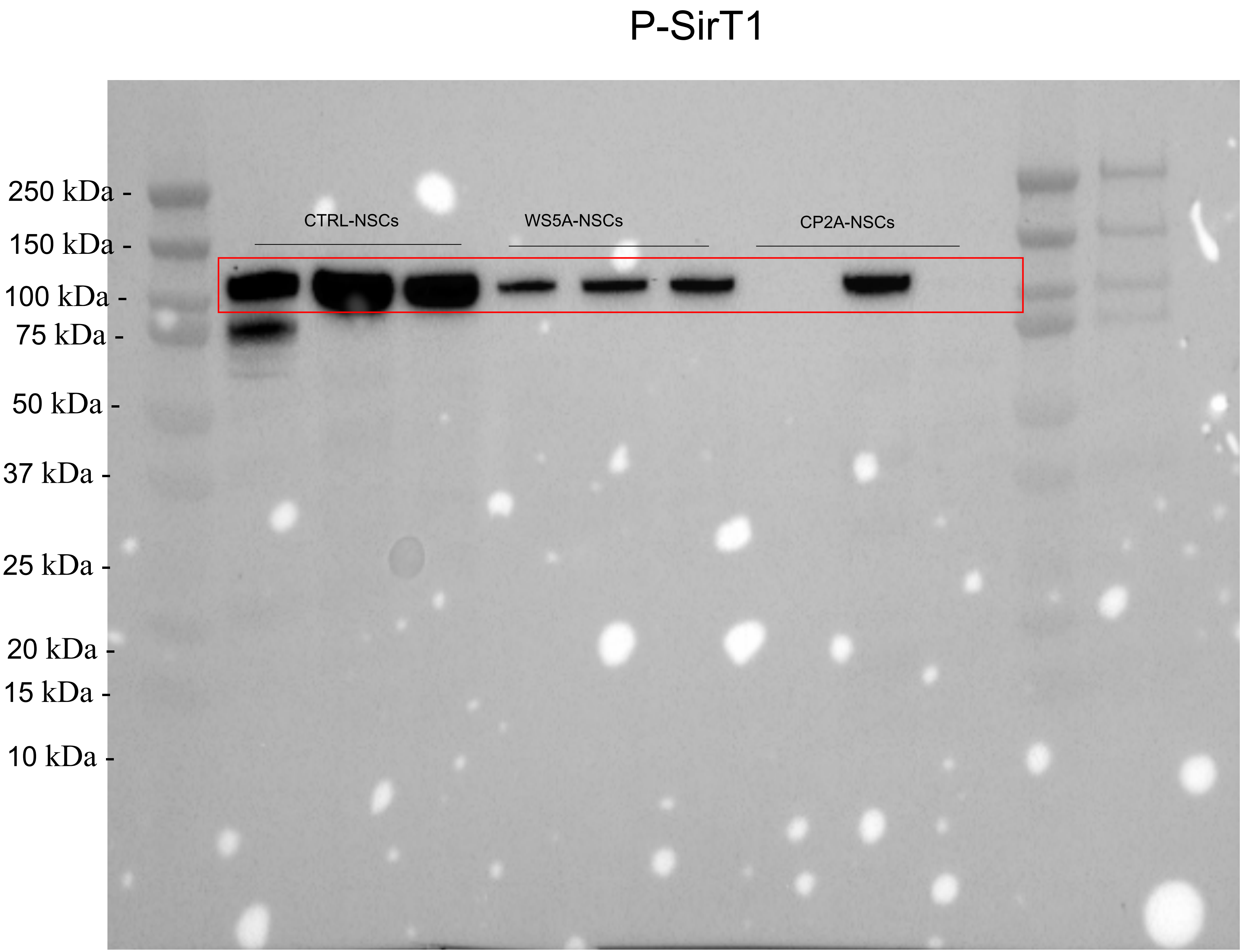
P-SirT1
250 kDa -
150 kDa -
100 kDa -
75 kDa -
50 kDa -
37 kDa -
25 kDa -
20 kDa -
15 kDa -
10 kDa -
CTRL-NSCs
WS5A-NSCs
CP2A-NSCs

Fig. 8K

LC3

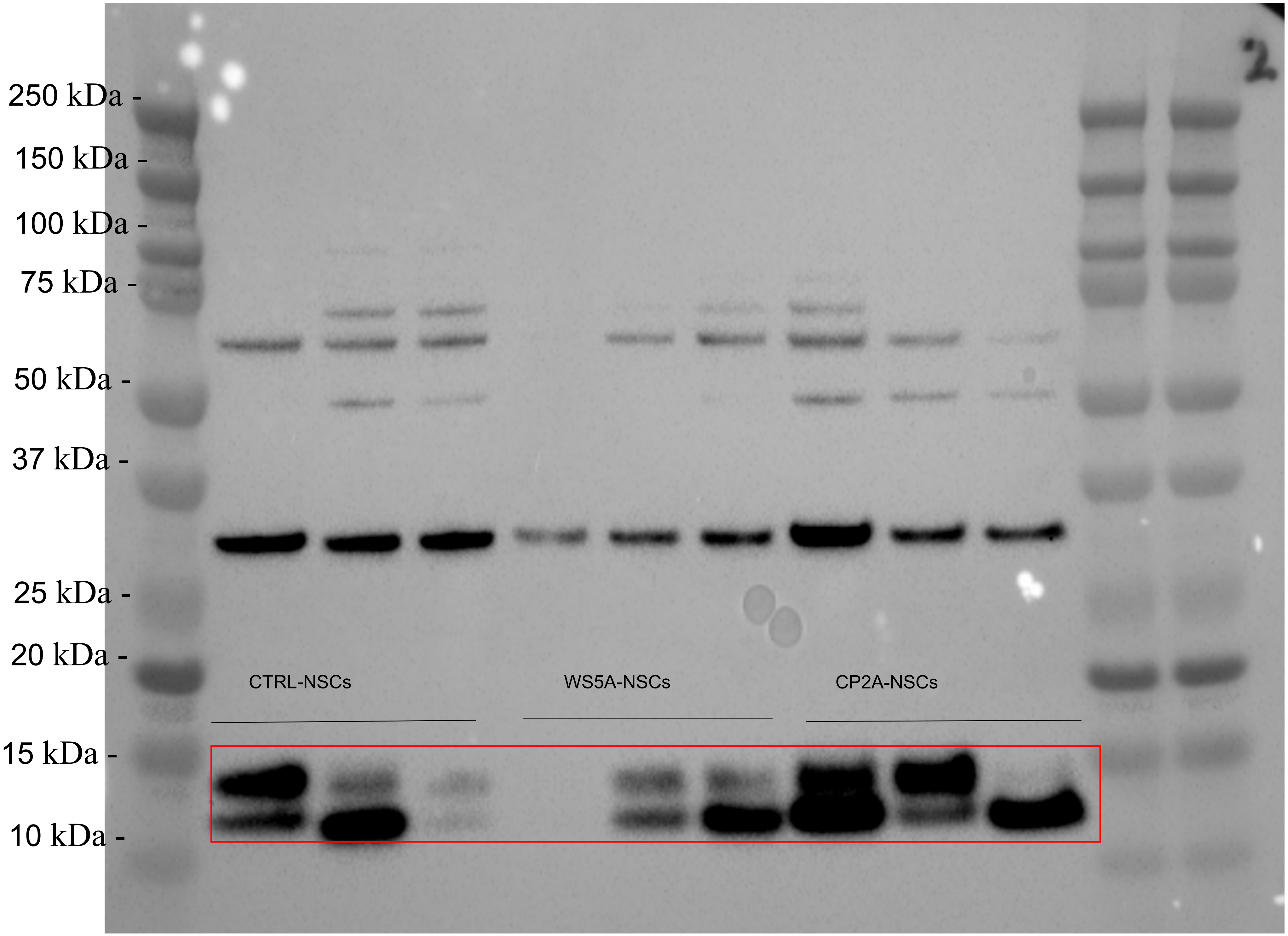
250 kDa -
150 kDa -
100 kDa -
75 kDa -
50 kDa -
37 kDa -
25 kDa -
20 kDa -
15 kDa -
10 kDa -
CTRL-NSCs
WS5A-NSCs
CP2A-NSCs

Fig. 8K

β-ACTIN (P-SirT1, LC3 gel)

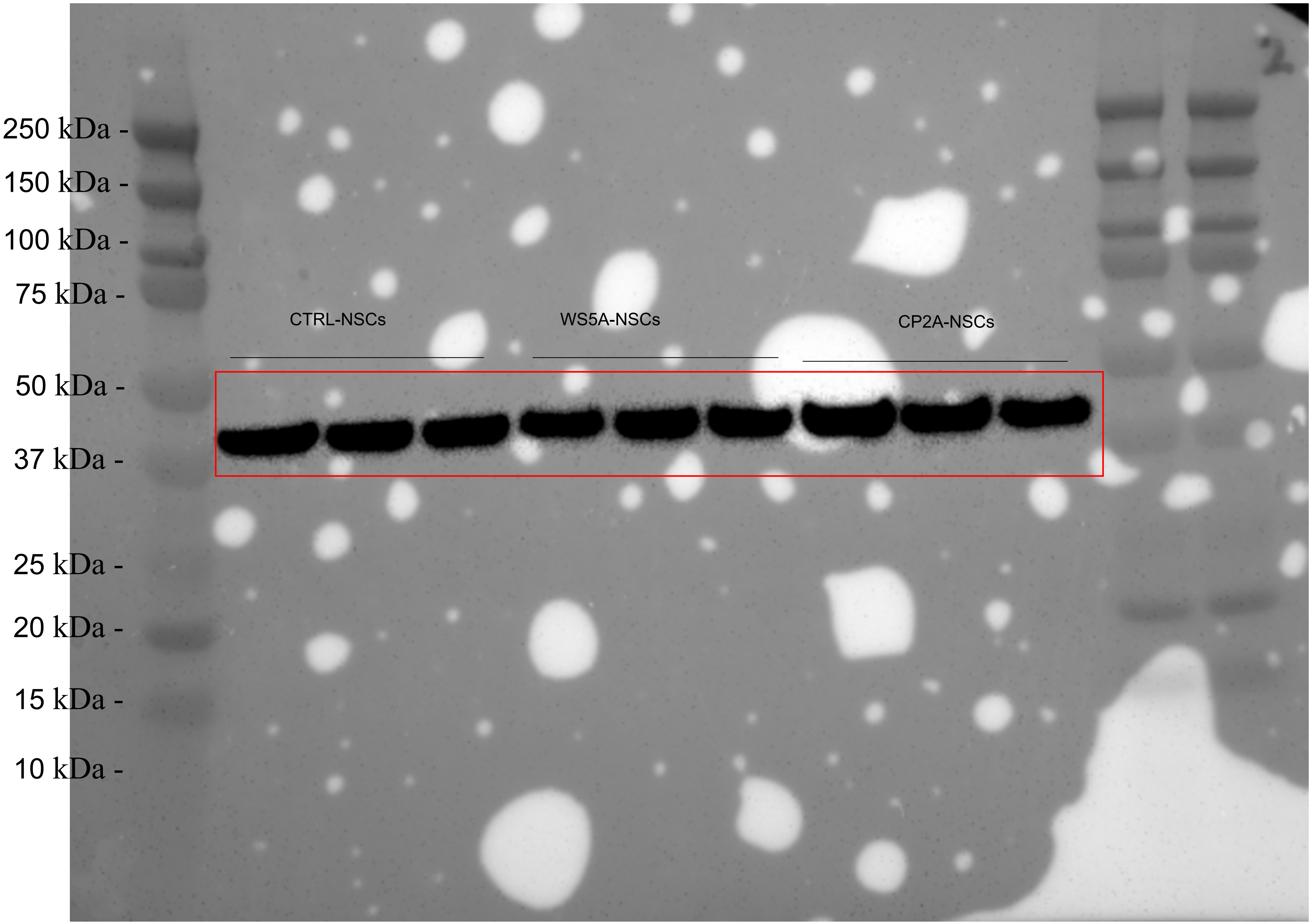

Fig. 8K

PINK1

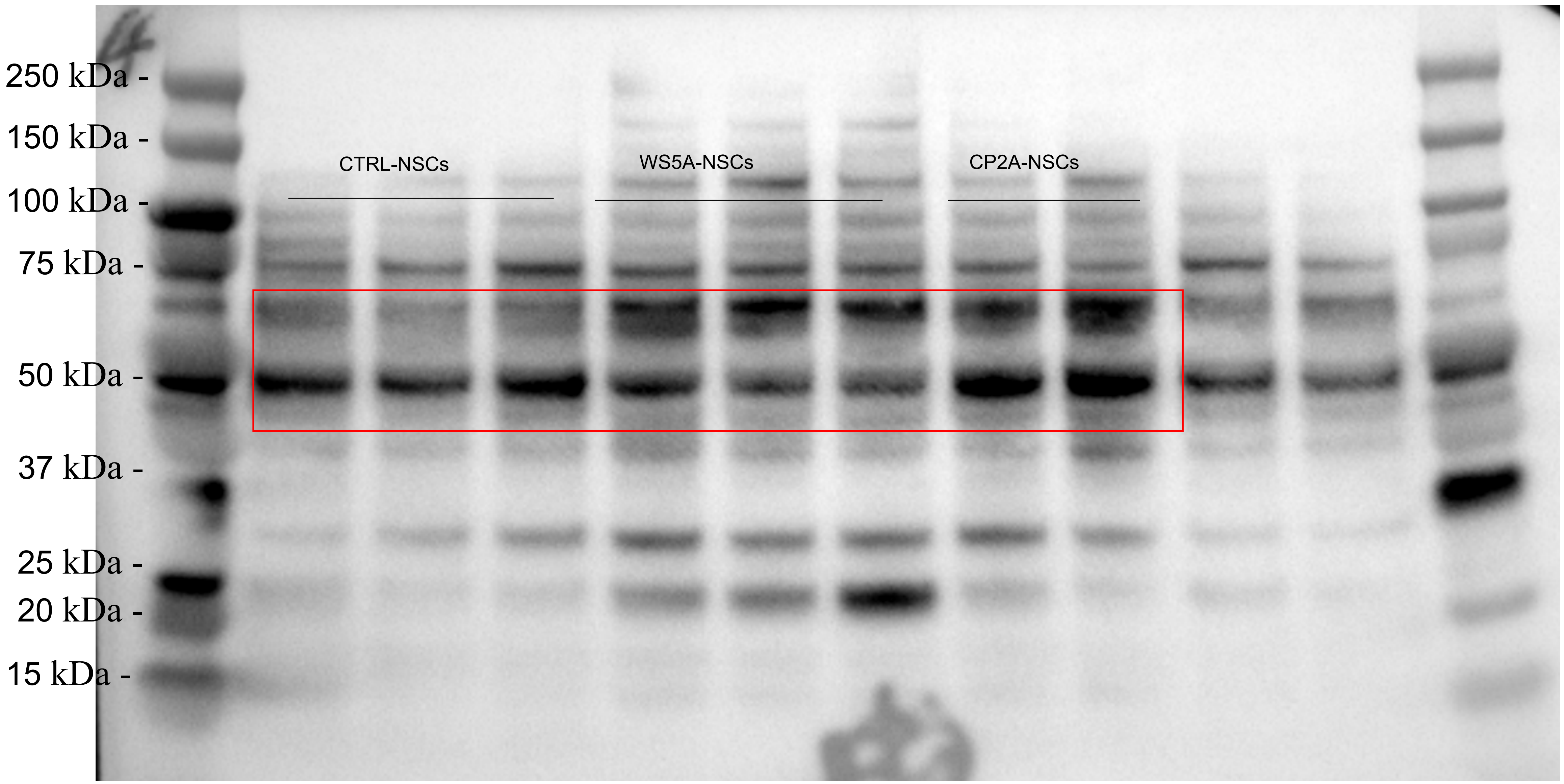

250 kDa -
150 kDa -
100 kDa -
75 kDa -
50 kDa -
37 kDa -
25 kDa -
20 kDa -
15 kDa -
CTRL-NSCs
WS5A-NSCs
CP2A-NSCs

Fig. 8K

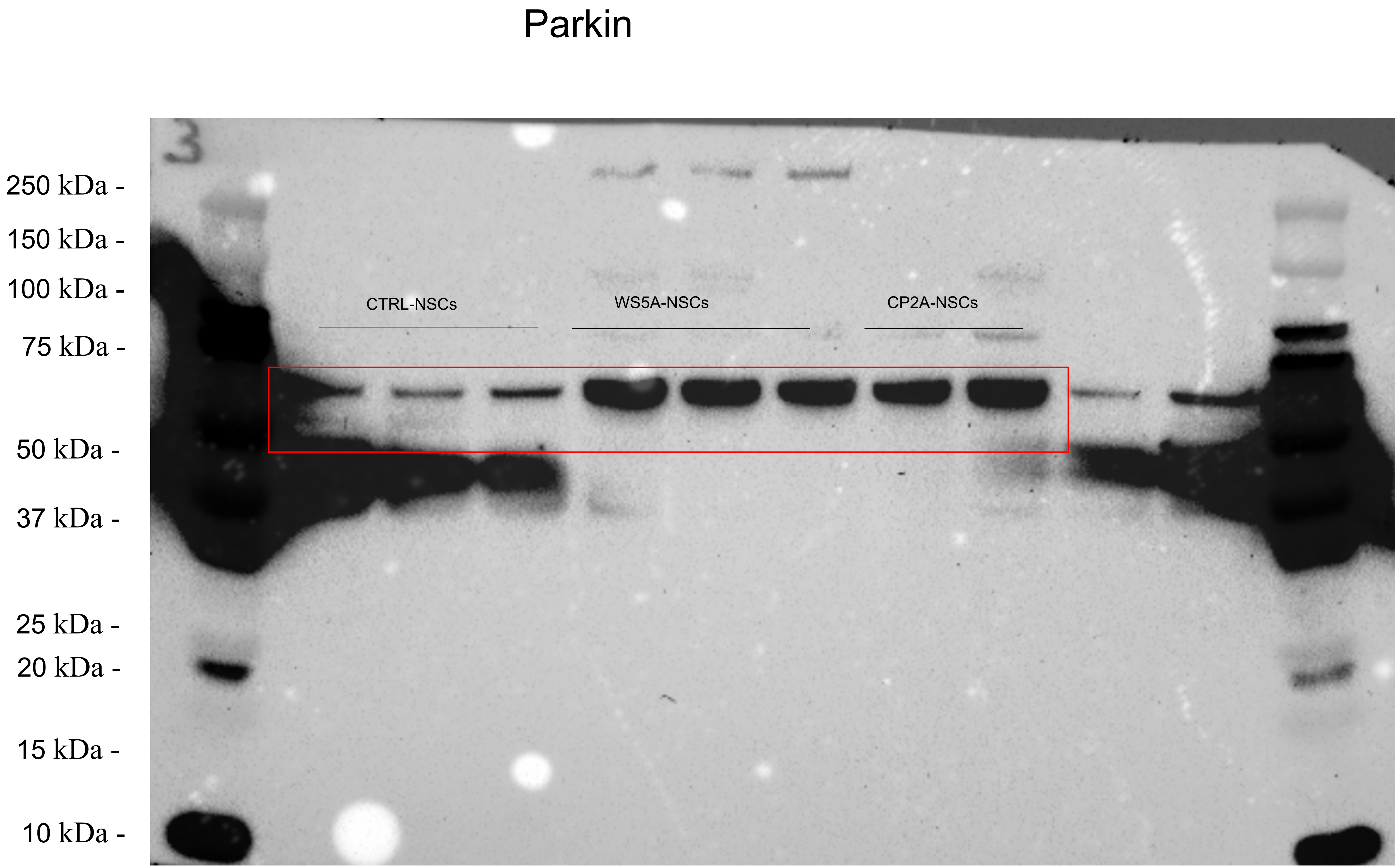
Parkin
250 kDa -
150 kDa -
100 kDa -
75 kDa -
50 kDa -
37 kDa -
25 kDa -
20 kDa -
15 kDa -
10 kDa -
CTRL-NSCs
WS5A-NSCs
CP2A-NSCs

Fig. 8K

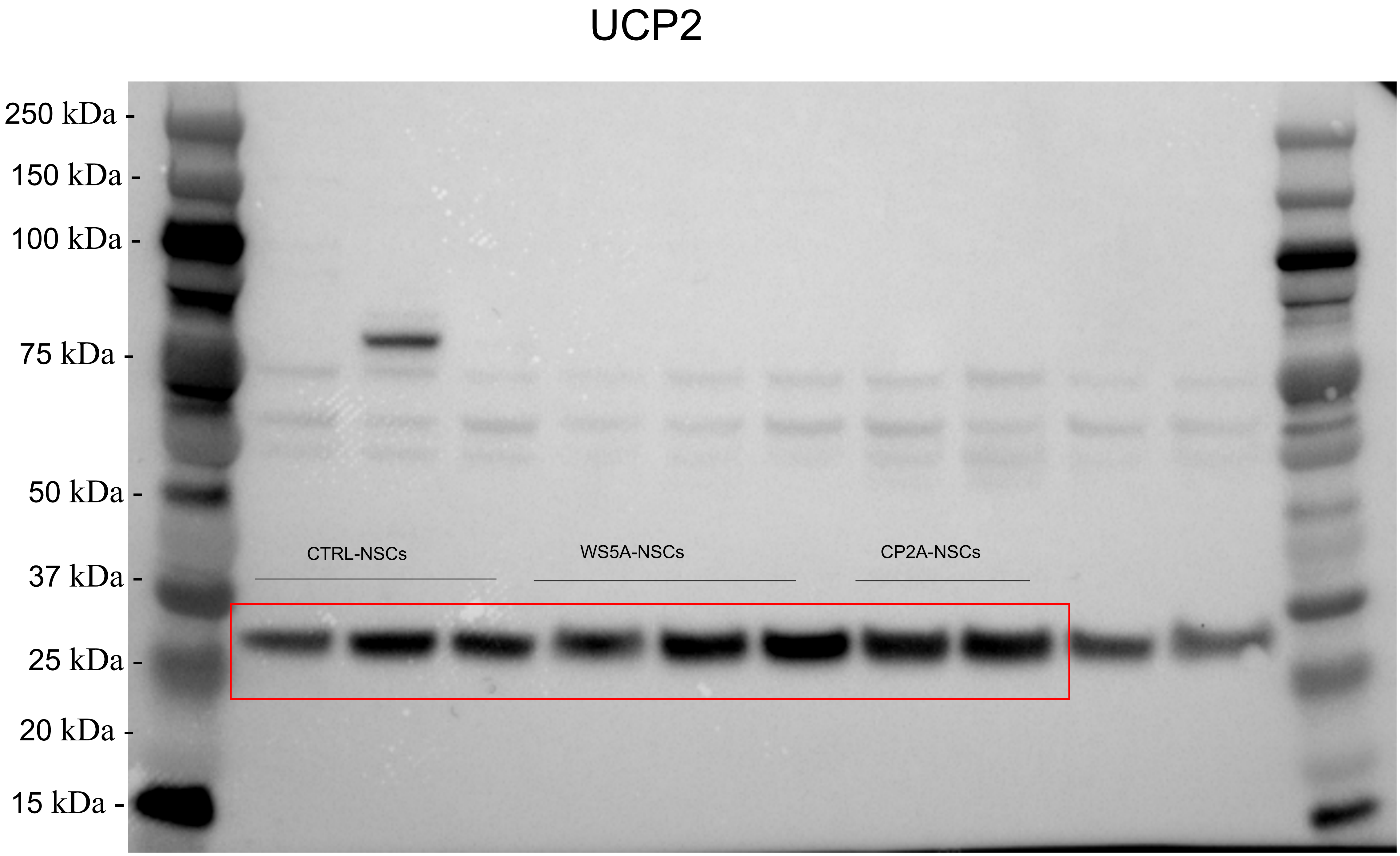
UCP2
250 kDa -
150 kDa -
100 kDa -
75 kDa -
50 kDa -
37 kDa -
25 kDa -
20 kDa -
15 kDa -
CTRL-NSCs
WS5A-NSCs
CP2A-NSCs

Fig. 8K

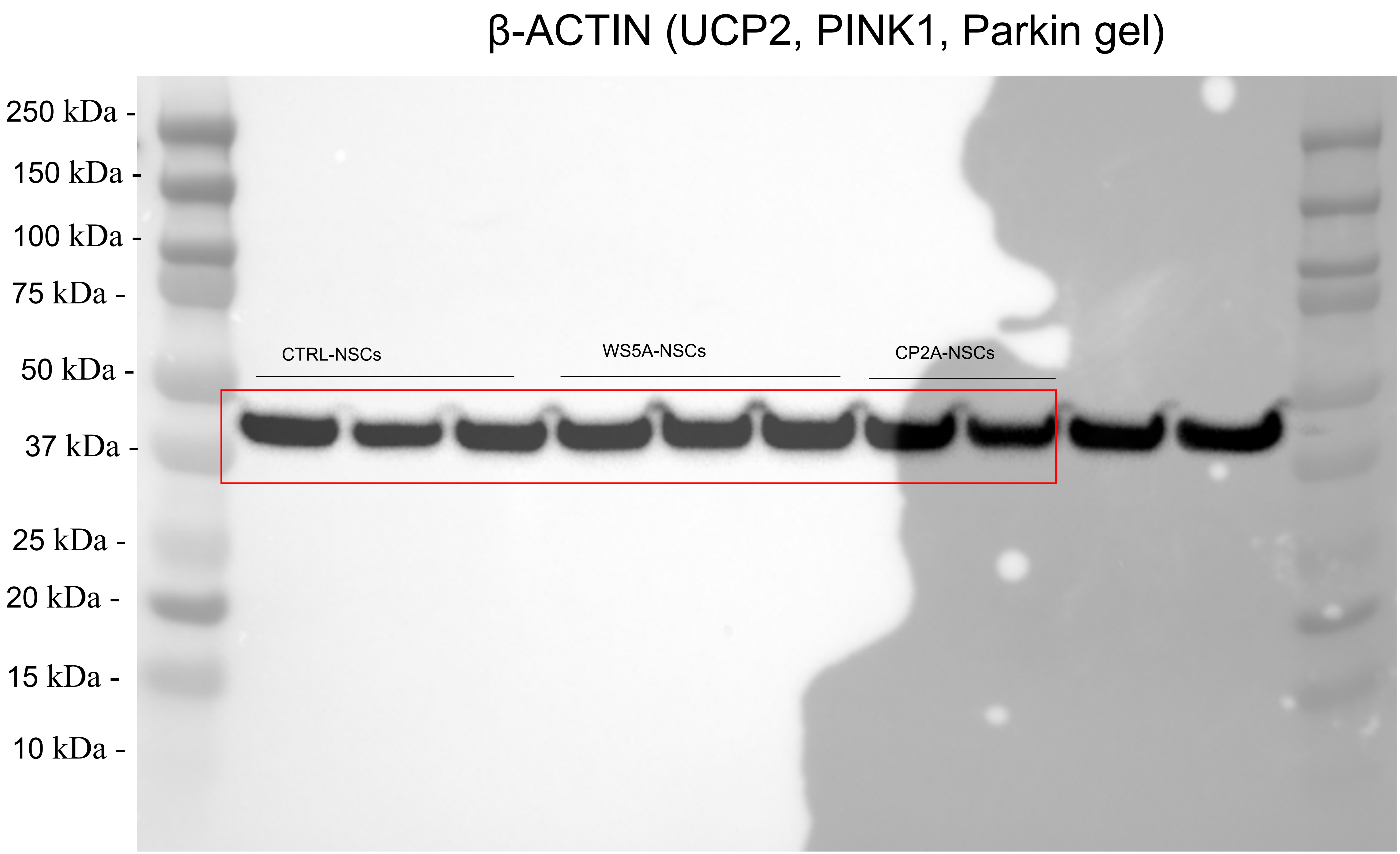
β-ACTIN (UCP2, PINK1, Parkin gel)
250 kDa -
150 kDa -
100 kDa -
75 kDa -
50 kDa -
37 kDa -
25 kDa -
20 kDa -
15 kDa -
10 kDa -
CTRL-NSCs
WS5A-NSCs
CP2A-NSCs

Fig. 8K

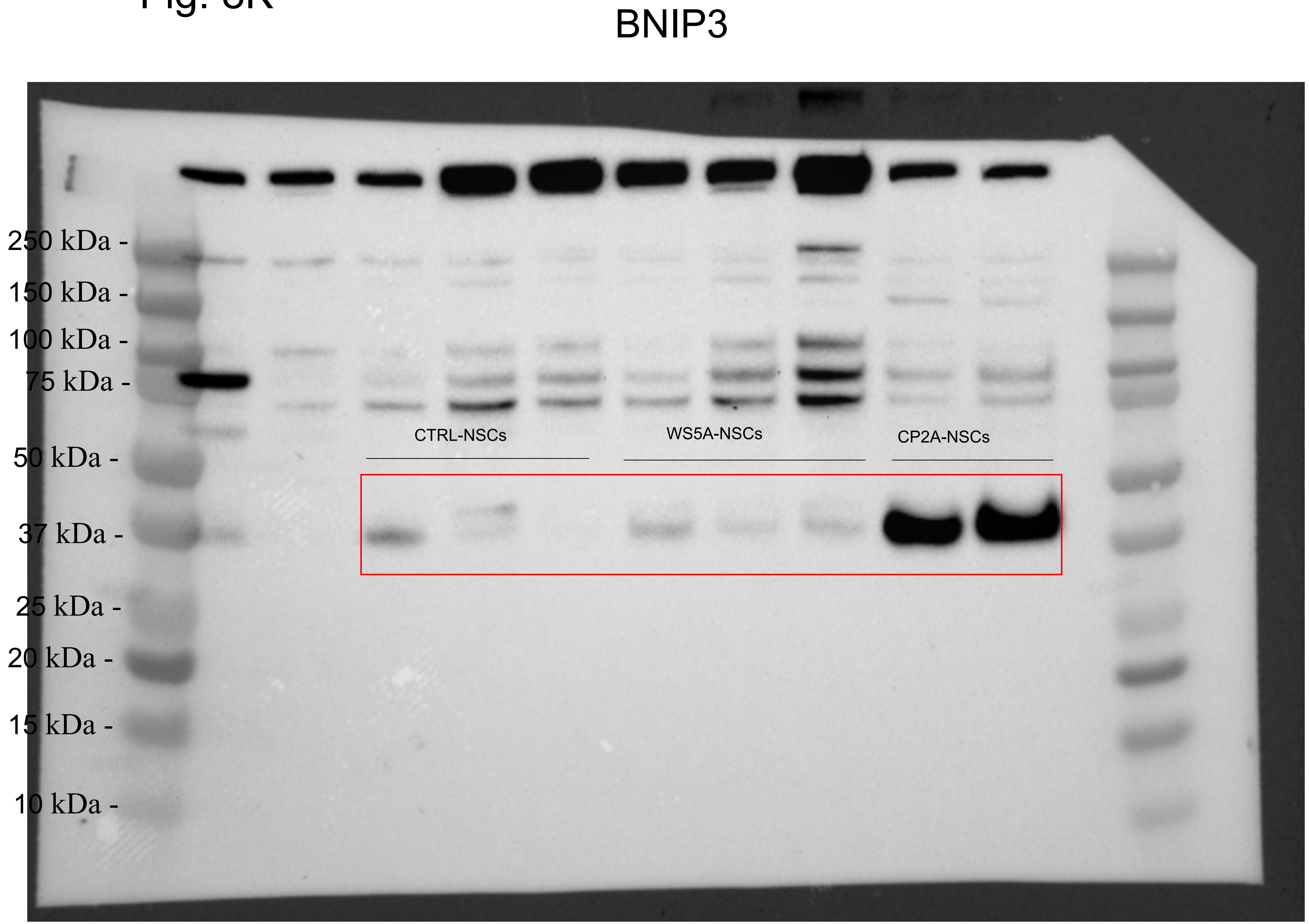

BNIP3
250 kDa -
150 kDa -
100 kDa -
75 kDa -
50 kDa -
37 kDa -
25 kDa -
20 kDa -
15 kDa -
10 kDa -
CTRL-NSCs
WS5A-NSCs
CP2A-NSCs

Fig. 8K

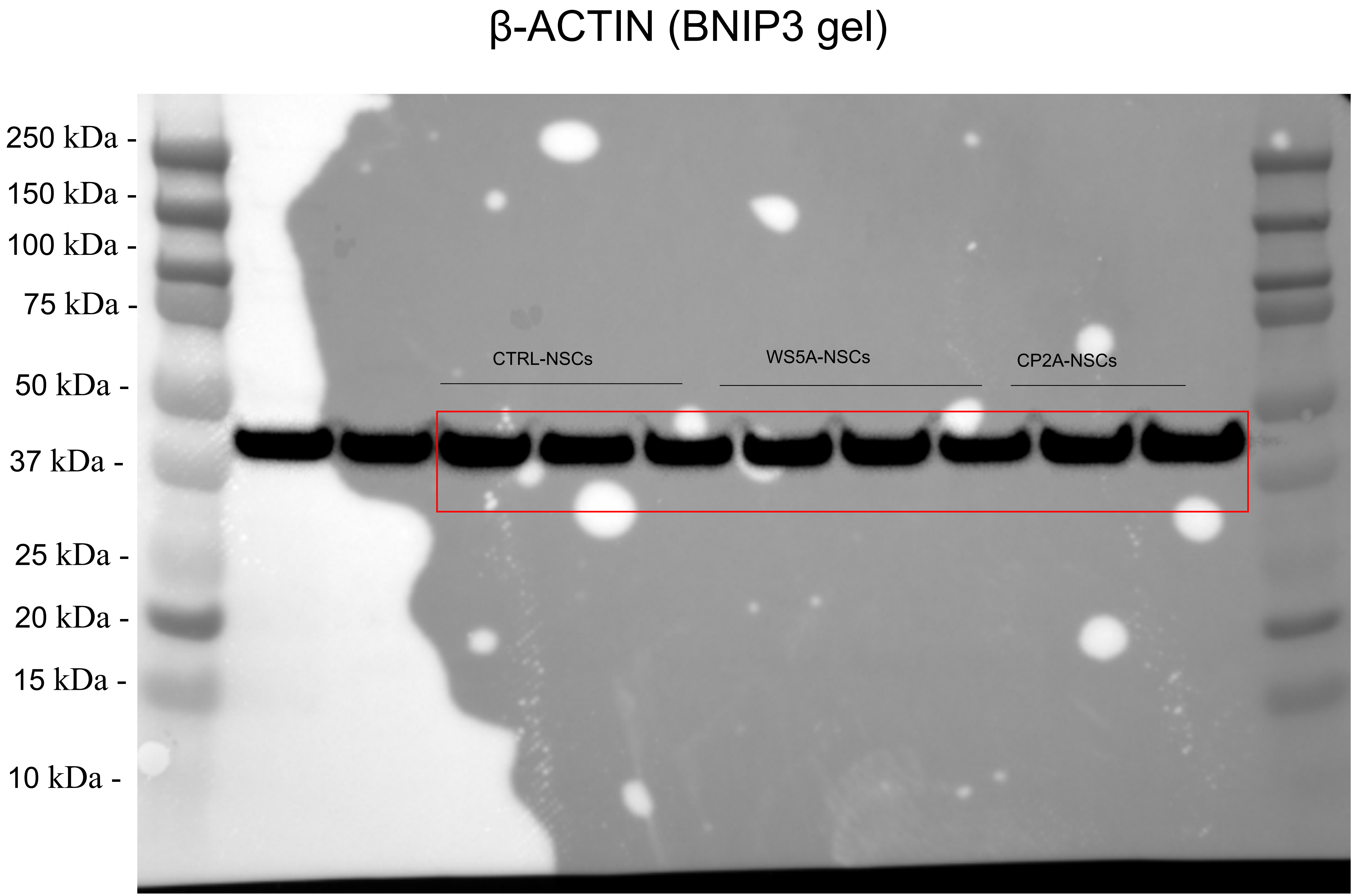
β-ACTIN (BNIP3 gel)
250 kDa -
150 kDa -
100 kDa -
75 kDa -
50 kDa -
37 kDa -
25 kDa -
20 kDa -
15 kDa -
10 kDa -
CTRL-NSCs
WS5A-NSCs
CP2A-NSCs
